# Supplementary material for: Human and animal skin identified by palaeoproteomics in Scythian leather objects from Ukraine
Source: PLoS One. 2023 Dec 13;18(12):e0294129. doi: 10.1371/journal.pone.0294129 (PMC10718408; doi:10.1371/journal.pone.0294129)
Supplement: S1 File — Table S1.1. Detailed descriptions and images of samples used in the study. Table S1.2. Protein summary for sample 21 from Bulhakovo, kurgan 5, burial 2. Table S1.3. Specific peptides from sample 21 from Bulhakovo, kurgan 5, burial 2. Table S1.4. Protein summary for sample 48 from Ilyinka, kurgan 4 burial 3. Table S1.5. Specific peptides from sample 48 from Ilyinka, kurgan 4 burial 3. Table S1.6. Suspected S. vulgaris peptides in sample 48 from Ilyinka, kurgan 4 burial 3 and their Marmotini equivalents. Table S1.7. Specific peptides from sample 49 from Vil’na Ukraina 4, kurgan 22 burial 1. Table S1.8. Protein summary for sample 49 from Vil’na Ukraina 4, kurgan 22 burial 1. Text. Detailed LC-MS/MS method and discussion of results. SI references. (PDF) [file pone.0294129.s001.pdf]

SUPPORTING INFORMATION 1

Human and animal skin identified by palaeoproteomics in Scythian leather objects from Ukraine

Authors

Luise Ø. Brandt[1], Meaghan Mackie[1, 2], Marina Daragan[3], Matthew, J. Collins[1,4], Margarita Gleba[5]

Affiliations

[1] The Globe Institute, University of Copenhagen, Øster Farimagsgade 5, Bygning 7, 1353 Copenhagen K, Denmark

[2] Novo Nordisk Foundation Center for Protein Research, University of Copenhagen, Blegdamsvej 3b, 2200 Copenhagen N, Denmark

[3] Institute of Archaeology of the National Academy of Sciences of Ukraine, Volodymyr Ivasyuk Avenue 12, 04210 Kiev, Ukraine

[4] University of Cambridge, McDonald Institute for Archaeological Research, 2.8 Henry Wellcome Building, Fitzwilliam St, Cambridge CB2 1QH, UK

[5] Dipartimento dei Beni Culturali, Università degli Studi di Padova, Piazza Capitaniato 7, 35139 Padova, Italy

This PDF file includes:

Table S1.1. Detailed descriptions and images of samples used in the study.
Table S1.2. Protein summary for sample 21 from Bulhakovo, kurgan 5, burial 2
Table S1.3. Specific peptides from sample 21 from Bulhakovo, kurgan 5, burial 2
Table S1.4. Protein summary for sample 48 from Ilyinka, kurgan 4 burial 3
Table S1.5. Specific peptides from sample 48 from Ilyinka, kurgan 4 burial 3
Table S1.6. Suspected *S. vulgaris* peptides in sample 48 from Ilyinka, kurgan 4 burial 3 and their Marmotini equivalents
Table S1.7. Specific peptides from sample 49 from Vil'na Ukraina 4, kurgan 22 burial 1
Table S1.8. Protein summary for sample 49 from Vil'na Ukraina 4, kurgan 22 burial 1
Text. Detailed LC-MS/MS method and discussion of results
SI references

Table S1. Detailed descriptions and images of samples used in the study.

Sample no.	Site, kurgan, burial, object, museum	Image of the fragment from which the sample was taken	Sample location description
1	Ilyinka,<br>kurgan 4,<br>burial 3,<br>quiver 2<br><br>Zaporizhzhya Regional Museum, Zaporizhzhya, Ukraine	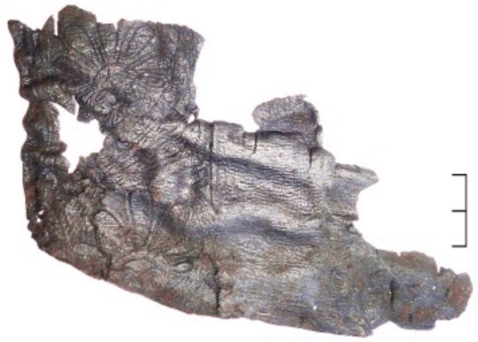	Sample taken from fragments at the top of the quiver (the largest, possibly back fragment illustrated)
2	Ilyinka,<br>kurgan 4,<br>burial 3,<br>quiver 2<br><br>Zaporizhzhya Regional Museum, Zaporizhzhya, Ukraine	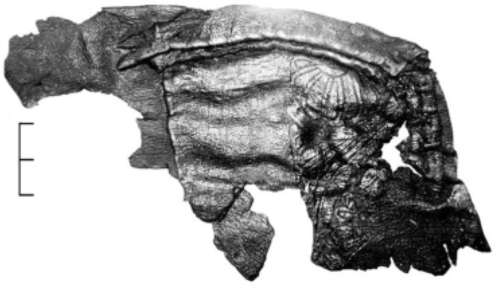	Sample taken from fragments at the top of the quiver (the largest, possibly back fragment illustrated)
3	Ilyinka,<br>kurgan 4,<br>burial 3,<br>quiver 2<br><br>Zaporizhzhya Regional Museum, Zaporizhzhya, Ukraine	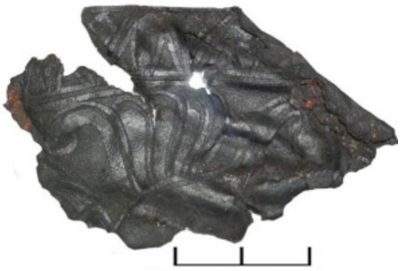	Sample taken from fragments at the middle of the quiver (the largest fragment illustrated)
4	Ilyinka,<br>kurgan 4,<br>burial 3,<br>quiver 2<br><br>Zaporizhzhya Regional, Museum, Zaporizhzhya, Ukraine	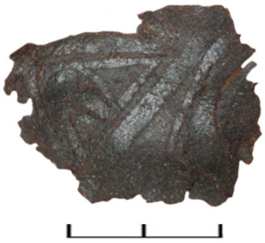	Sample taken from fragments of the lower part of the quiver
5	Ilyinka,<br>kurgan 4,<br>burial 3,<br>quiver 2<br><br>Zaporizhzhya Regional Museum, Zaporizhzhya, Ukraine	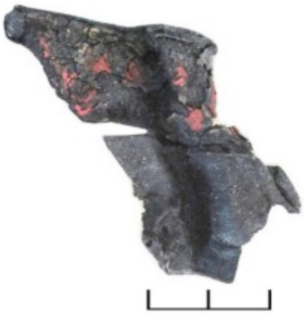	Sample taken from the ribbon in the middle of the quiver (the largest fragment illustrated)

7	Ilyinka,<br>kurgan 4,<br>burial 2,<br>quiver 5<br><br>Zaporizhzhya Regional Museum, Zaporizhzhya, Ukraine	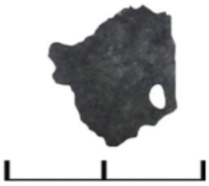	Sample taken from an indeterminate part of the quiver.
8	Ilyinka,<br>kurgan 4,<br>burial 2,<br>quiver 7<br><br>Zaporizhzhya Regional Museum, Zaporizhzhya, Ukraine	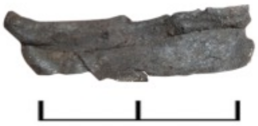	Sample taken from an indeterminate part of the quiver.
9	Ilyinka,<br>kurgan 4,<br>burial 6<br>quiver<br><br>Zaporizhzhya Regional Museum, Zaporizhzhya, Ukraine	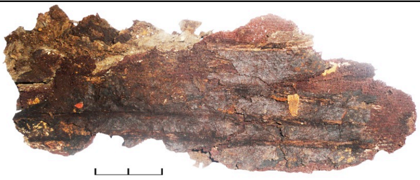	Sample taken from an indeterminate part of the quiver.
10	Ilyinka,<br>kurgan 4,<br>burial 2,<br>quiver 5<br><br>Zaporizhzhya Regional Museum, Zaporizhzhya, Ukraine	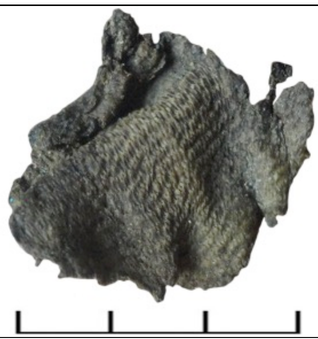	Sample taken from an indeterminate part of the quiver.
11	Ilyinka,<br>kurgan 4,<br>burial 2,<br>quiver number unknown<br><br>Zaporizhzhya Regional Museum, Zaporizhzhya, Ukraine	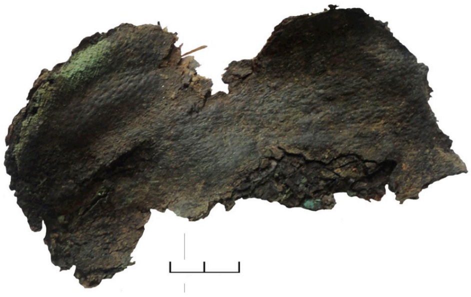	Sample taken from the only surviving fragment of an indeterminate part of a quiver
12	Ilyinka,<br>kurgan 4,<br>burial 2,<br>quiver 5<br><br>Zaporizhzhya Regional Museum, Zaporizhzhya, Ukraine	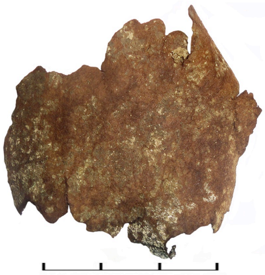	Sample taken from an indeterminate part of the quiver.
14	Ilyinka,<br>kurgan 4,<br>burial 3,<br>quiver 2	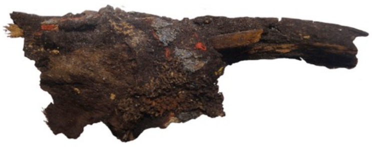	The sample was taken from the side, painted part of the quiver.

	Zaporizhzhya Regional Museum, Zaporizhzhya, Ukraine		
15	Sadove,<br>kurgan 4,<br>burial 1,<br>quiver<br><br>Institute of Archaeology of the National Academy of Science of Ukraine, Kyiv, Ukraine	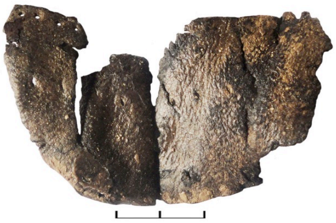	Sample taken from an indeterminate part of the quiver.
16	Bulhakovo,<br>kurgan 5,<br>burial 2,<br>quiver<br><br>Institute of Archaeology of the National Academy of Science of Ukraine, Kyiv, Ukraine	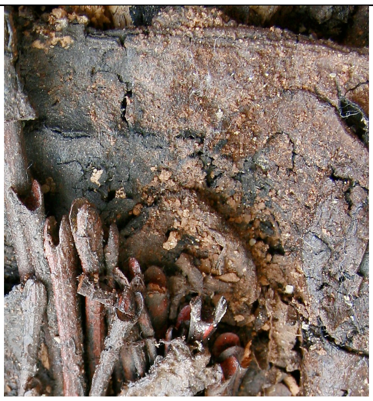	The sample was taken from the external upper back part of the quiver.
17	Bulhakovo,<br>kurgan 5,<br>burial 2,<br>quiver<br><br>Institute of Archaeology of the National Academy of Science of Ukraine, Kyiv, Ukraine	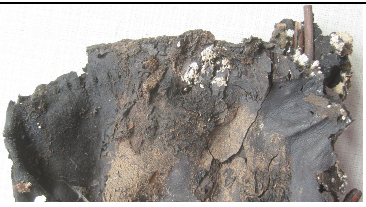	Sample taken from the top of the back of the quiver.
18	Bulhakovo,<br>kurgan 5,<br>burial 2,<br>quiver<br><br>Institute of Archaeology of the National Academy of Science of Ukraine, Kyiv, Ukraine	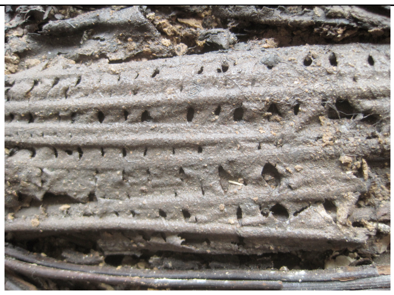	Sample taken from the middle, perforated part of the front wall of the quiver.
19	Vodoslavka,<br>kurgan 8,<br>burial 4,<br>quiver<br><br>Institute of Archaeology of the National Academy of Science of Ukraine, Kyiv, Ukraine	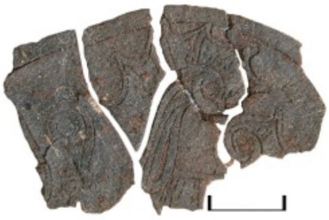	Preserved in separate fragments; sample taken from the front decorated part of the quiver.

20	Zolota Balka,<br>kurgan 13,<br>burial 7,<br>vessel<br><br>Institute of Archaeology of the National Academy of Science of Ukraine, Kyiv, Ukraine	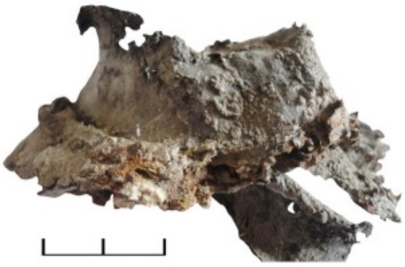	Sample taken from the top of the vessel.
21	Bulhakovo,<br>kurgan 5,<br>burial 2,<br>quiver<br><br>Institute of Archaeology of the National Academy of Science of Ukraine, Kyiv, Ukraine	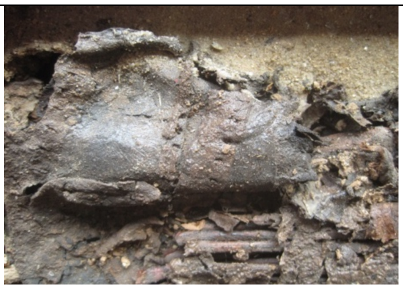	The sample was taken from the upper part of the quiver, from the separate decorative ribbon.
22	Bulhakovo,<br>kurgan 5,<br>burial 2,<br>quiver<br><br>Institute of Archaeology of the National Academy of Science of Ukraine, Kyiv, Ukraine	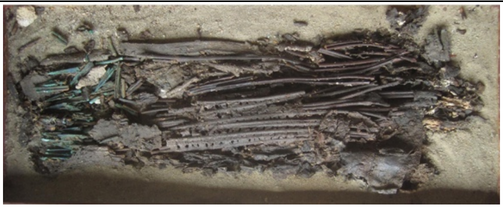	The sample was taken from the front part of the quiver (possibly same part as sample no. 18).
23	Kairy,<br>kurgan group V,<br>kurgan 1,<br>burial 1,<br>quiver<br><br>Institute of Archaeology of the National Academy of Science of Ukraine, Kyiv, Ukraine	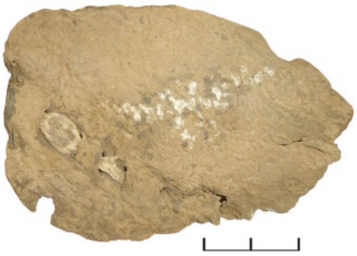	Sample taken from the bottom of the quiver.
24	Ol'hyne,<br>kurgan 2,<br>burial 1,<br>quiver<br><br>Institute of Archaeology of the National Academy of Science of Ukraine, Kyiv, Ukraine	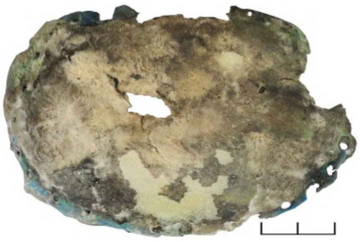	Sample taken from the bottom of the quiver.

25	Kairy,<br>kurgan group V,<br>kurgan 1,<br>burial 1,<br>possible trousers<br><br>Institute of Archaeology of the National Academy of Science of Ukraine, Kyiv, Ukraine	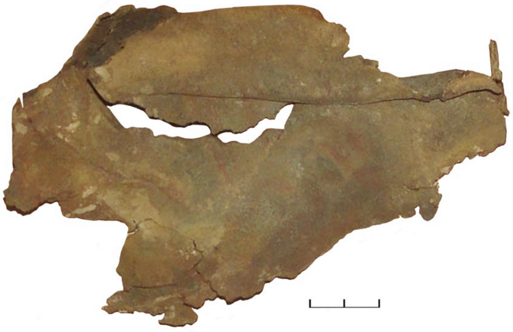	Sample taken from pieces of leather described in the excavation report as trousers.
26	Bulhakovo,<br>kurgan 5,<br>burial 2,<br>quiver<br><br>Institute of Archaeology of the National Academy of Science of Ukraine, Kyiv, Ukraine	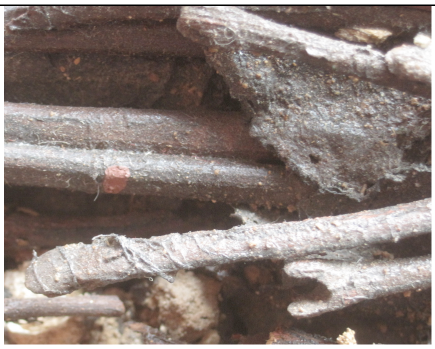	The sample was taken from the inner partition wall of the quiver.
27	Vodoslavka,<br>kurgan 8,<br>burial 4,<br>quiver<br><br>Institute of Archaeology of the National Academy of Science of Ukraine, Kyiv, Ukraine	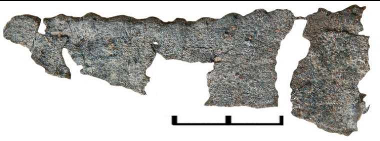	Sample taken from an indeterminate ecternal part of the quiver.
28	Vodoslavka,<br>kurgan 8,<br>burial 4,<br>quiver<br><br>Institute of Archaeology of the National Academy of Science of Ukraine, Kyiv, Ukraine	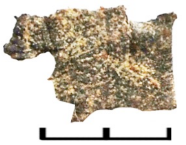	Sample taken from an indeterminate part of the quiver.
30	Bulhakovo,<br>kurgan 5,<br>burial 2,<br>quiver<br><br>Institute of Archaeology of the National Academy of Science of Ukraine, Kyiv, Ukraine	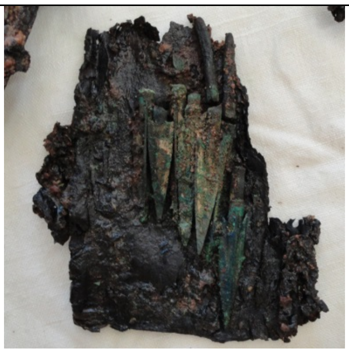	Sample taken from the lower back wall of the quiver.

30a	Orikhove,<br>kurgan 3,<br>burial 2,<br>quiver 3<br><br>Museum of Archaeology of Oles Honchar, Dnipro National University, Dnipro, Ukraine	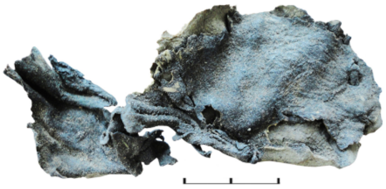	Sample taken from the bottom of the quiver.
31	Orikhove,<br>kurgan 3,<br>burial 2,<br>quiver 1<br><br>Museum of Archaeology of Oles Honchar, Dnipro National University, Dnipro, Ukraine	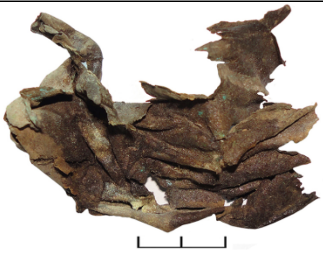	Sample taken from the side fragment of the quiver.
32	Orikhove,<br>kurgan 3,<br>burial 2,<br>quiver 2<br><br>Museum of Archaeology of Oles Honchar, Dnipro National University, Dnipro, Ukraine	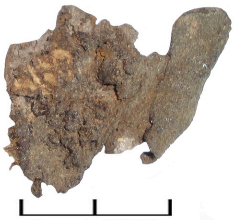	Sample taken from an indeterminate part of the quiver.
33	Orikhove,<br>kurgan 3,<br>burial 2,<br>quiver 2<br><br>Museum of Archaeology of Oles Honchar, Dnipro National University, Dnipro, Ukraine	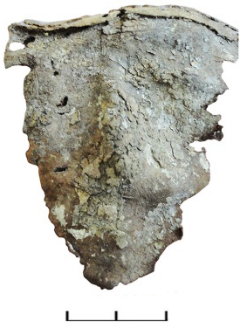	Sample taken from the likely decorative part of the quiver
34	Orikhove,<br>kurgan 3,<br>burial 2,<br>quiver 2<br><br>Museum of Archaeology of Oles Honchar, Dnipro National University, Dnipro, Ukraine	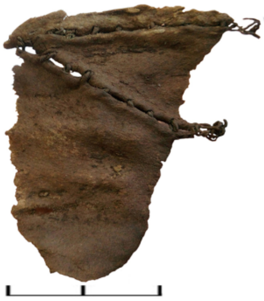	Sample taken from the likely decorative part of the quiver
35	Orikhove,<br>kurgan 3,<br>burial 2,<br>quiver 2<br><br>Museum of Archaeology of Oles Honchar, Dnipro National University, Dnipro, Ukraine	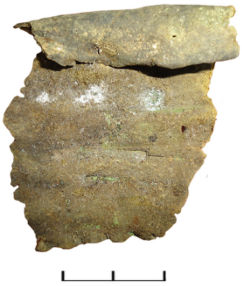	Sample taken from an indeterminate part of the quiver.

36	Zelene,<br>kurgan group I,<br>kurgan 2,<br>burial 3,<br>chamber 2,<br>quiver<br><br>Kherson Regional Museum, Kherson, Ukraine	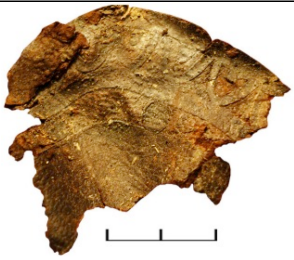	Sample taken from an indeterminate external, decorated part of the quiver.
37	Zelene,<br>kurgan group I,<br>kurgan 2,<br>burial 3,<br>chamber 1,<br>quiver 1<br><br>Kherson Regional Museum, Kherson, Ukraine	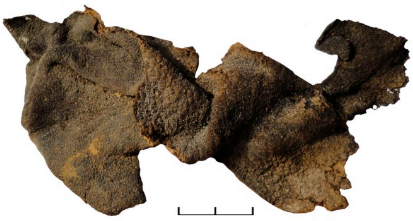	Sample taken from an indeterminate part of the quiver.
38	Zelene,<br>kurgan group I,<br>kurgan 2,<br>burial 3,<br>chamber 1,<br>quiver 1<br><br>Kherson Regional Museum, Kherson, Ukraine	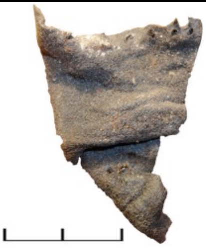	Sample taken from an indeterminate part of the quiver.
39	Kislychevate,<br>kurgan group 2,<br>kurgan 7,<br>burial 1,<br>quiver<br><br>Museum of Archaeology of Oles Honchar, Dnipro National University, Dnipro, Ukraine		Sample taken from an indeterminate part of the quiver.
40	Ilyinka,<br>kurgan 4,<br>burial 3,<br>quiver<br><br>Zaporizhzhya Regional Museum, Zaporizhzhya, Ukraine	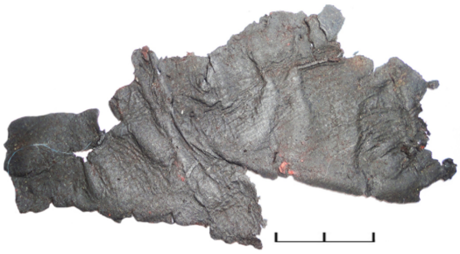	Sample taken from an indeterminate, external part of the quiver.
41	Vodoslavka,<br>kurgan 8,<br>burial 4,<br>quiver<br><br>Institute of Archaeology of the National Academy of Science of Ukraine, Kyiv, Ukraine	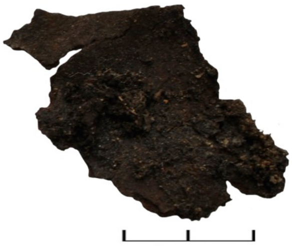	Sample taken from an indeterminate, external part of the quiver.

42	Otradne,<br>kurgan 3,<br>burial 2,<br>possible quiver<br><br>Institute of Archaeology of the National Academy of Science of Ukraine, Kyiv, Ukraine	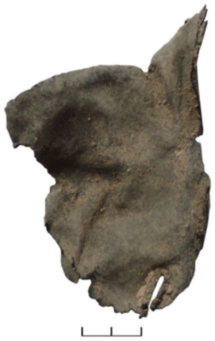	Sample taken from an indeterminate part of the quiver.
43	Otradne,<br>kurgan 3,<br>burial 1,<br>possible quiver<br><br>Institute of Archaeology of the National Academy of Science of Ukraine, Kyiv, Ukraine	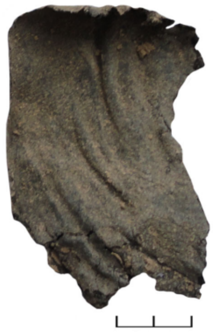	Sample taken from an indeterminate part of the quiver.
44	Tyahinka,<br>kurgan 8,<br>burial 3,<br>quiver<br><br>Institute of Archaeology of the National Academy of Science of Ukraine, Kyiv, Ukraine	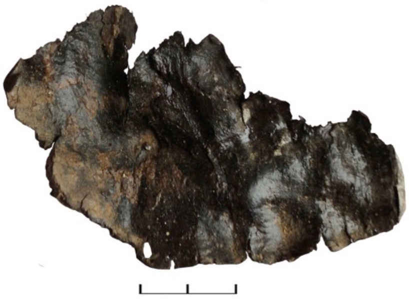	Sample taken from the only surviving indeterminate part of the quiver.
45	Vysup'sk,<br>kurgan 6,<br>burial 1,<br>mirror case<br><br>Institute of Archaeology of the National Academy of Science of Ukraine, Kyiv, Ukraine	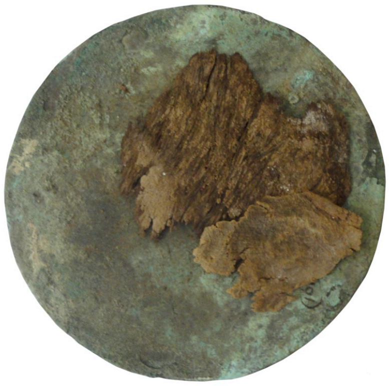	Sample taken from the surviving fragment of a mirror case.
46	Vodoslavka,<br>kurgan 8,<br>burial 4,<br>quiver<br><br>Institute of Archaeology of the National Academy of Science of Ukraine, Kyiv, Ukraine	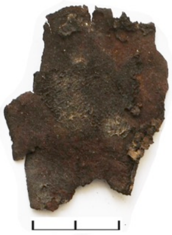	Sample taken from an indeterminate part of the quiver.

47	Kairy,<br>kurgan group V,<br>kurgan 1,<br>burial 1<br><br>Institute of Archaeology of the National Academy of Science of Ukraine, Kyiv, Ukraine	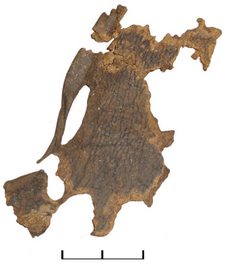	Sample taken from an indeterminate part of the quiver or garment
48	Ilyinka,<br>kurgan 4,<br>burial 3<br><br>Zaporizhzhya Regional Museum, Zaporizhzhya, Ukraine	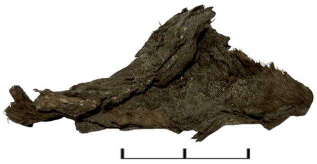	Sample from fur remains found in association with textile.
49	Vil'na Ukraina 3,<br>kurgan 22,<br>burial 1,<br>mirror case or garment<br><br>Institute of Archaeology of the National Academy of Science of Ukraine, Kyiv, Ukraine	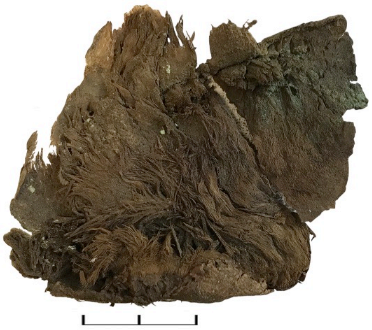	Sample taken from a fragment preserving only pellage.

Table S2. Protein summary for sample 21 from Bulhakovo, kurgan 5, burial 2 (Raw file ID: MG21)

Protein no.	Leading Razor Protein	Species Specificity	No. of Identified Razor+ Unique Peptides	% Coverage (not razor)	No. of MS2 spectra (razor)
**1**	sp|P02452|Collagen alpha-1(I) chain	Homininae	59	41.5	99
**2**	tr|A0A087WTA8| Collagen alpha-2(I) chain	Catarrhini	36	35.1	50
**3**	sp|P12883| Myosin-7	Euteleostomi + *Aequorea victoria*	35	7.3	51
**4**	tr|A0A0A0MTS7| Titin	Hominidae	23	0.6	24
**5**	sp|P02461|Collagen alpha-1(III) chain	Simiiformes	14	13.5	24
**6**	sp|P16615-2|Sarcoplasmic/endoplasmic reticulum calcium ATPase 2	Amniota	14	10	14
**7**	tr|F8VZE0|Myosin-binding protein C, slow-type	*aspecific*	13	7.8	17
**8**	sp|Q9UKX2|Myosin-2	*Homo sapiens, Ailuropoda melanoleuca*	13	8.3	16
**9**	sp|P06576|synthase subunit beta, mitochondrial	*aspecific*	11	16.1	12
**10**	tr|F2Z2F1|Myoglobin	Boreoeutheria	10	35.8	13
**11**	tr|A0A494C1A0| Alpha-actinin-2	*aspecific*	9	8.5	12
**12**	sp|P12235|ADP/ATP translocase 1	Euteleostomi	9	26.,5	9
**13**	tr|A0A087WYX9|Collagen alpha-2(V) chain	Primates, Artiodactyla, *Equus* sp., *Galeopterus variegatus, Buceros rhinoceros silvestris, Bucorvus abyssinicus*	7	9.7	8
**14**	sp|P63267-2|Actin, gamma-enteric smooth muscle	*aspecific*	7	17.4	7
**15**	tr|E9PCT5|Caveolin	Catarrhini	6	22.8	7
**16**	tr|A0A3B3ITQ8|Collagen alpha-2(IV) chain	*Homo sapiens, Sturnira hondurensis*	5	13.6	8
**17**	tr|I3L1P8|Mitochondrial 2-oxoglutarate/malate carrier protein	Euteleostomi	5	21.6	5
**18**	sp|P20908-2|Collagen alpha-1(V) chain	*aspecific*	5	3.8	5
**19**	sp|P62805|Histone H4	*aspecific*	5	39,8	8
**20**	tr|E9PP49|Collagen alpha-2(VIII) chain	Eutheria	4	6.3	4
**21**	sp|P02748|Complement component C9	Homininae	4	9.1	6
**22**	sp|Q86TD4-3|Sarcalumenin	Boreoeutheria	4	7.2	4
**23**	tr|A0A0C4DGV8|Semaphorin-3B	Catarrhini + *Tupaia chinensis*	3	4.8	3

**24**	tr|C9JKR2|Albumin	Catarrhini + *Sturnira hondurensis* + *Ailuropoda melanoleuca*	3	5.0	3
**25**	tr|F5H0C7|ADP-ribosylation factor 3	*aspecific*	3	23.5	3
**26**	tr|G3V3A0|Alpha-1-antichymotrypsin	Homininae + *Sturnira hondurensis*	3	20	4
**27**	sp|O14983-2|Sarcoplasmic/endoplasmic reticulum calcium ATPase 1	Theria	3	3.6	3
**28**	sp|O75746-2|Calcium-binding mitochondrial carrier protein Aralar1	*aspecific*	3	5.6	3
**29**	sp|Q14011|Cold-inducible RNA-binding protein	Tetrapoda	3	25	4
**30**	sp|Q14315-2|Filamin-C	*aspecific*	3	0.9	3
**31**	sp|Q9HCP6|Protein-cysteine N-palmitoyltransferase HHAT-like protein	Simiiformes	3	4.6	3
**32**	tr|B1ALM3|Voltage-dependent L-type calcium channel subunit alpha	*Homo sapiens*	2	1.2	2
**33**	tr|C9JI87|Voltage-dependent anion-selective channel protein 1	Amniota	2	12	2
**34**	tr|D6RAA6|Transmembrane protein 33	*aspecific*	2	9.5	2
**35**	tr|J3QRJ8|Very long-chain-specific acyl-CoA dehydrogenase, mitochondrial	*aspecific*	2	11.3	2
**36**	sp|P78539-3|Sushi repeat-containing protein SRPX	*aspecific*	2	5.9	2
**37**	sp|Q5VXT5-2|Synaptophysin-like protein 2	Amniota	2	9.7	2

Table S3. Specific peptides from sample 21 from Bulhakovo, kurgan 5, burial 2 (Raw file ID: MG21)

Protein no.	Peptide	Species	Start position	End position	Score	No. of MS2
1	GEPGPVGVQGPPGPAGEEGK	Mammalia but not *Mus* sp.	449	468	208.66	2
	GESGPSGPAGPTGAR	Amniota but not *Mus* sp. (only Hominoidea is Homininae)	782	796	262.07	1
	AGPPGPAGPAGPPGPIGNVGAPGAK	Hominoidea + S*teptomyces* sp. + *Mus* sp.	838	862	155.93	1
2	TGEVGAVGPPGFAGEK	Catarrhini	829	844	158.25	1
3	LLSTLFANYAGAD	Euteleostomi + *Aequorea victoria*	616	628	163.32	1
4	IAVPITVITL	Hominidae	30213	30222	116.11	1
5	DGPPGPAGNTGAPGSPGVSGPK	Simiiformes	902	923	237.40	1
6	NYLEPAILE	Amniota	989	997	122.74	1
7	*aspecific*					
8	TLAQLFSGAQTA	*Homo sapiens, Ailuropoda melanoleuca*	619	630	118.91	1
9	*aspecific*					
10	GATVLTALGGILK	Boreoeutheria	66	78	144.65	1
11	*aspecific*					
12	DFLAGGVAAAVSK	Euteleostomi	11	23	81.92	1
13	VGPPGPAGAPGPAGPLGEPGK	Primates, Artiodactyla, *Equus* sp., *Galeopterus variegatus, Buceros rhinoceros silvestris, Bucorvus abyssinicus*	530	550	171.26	1
14	*aspecific*					
15	VYSIYVHTV	Catarrhini	136	144	155.51	1
16	PGVTGPKGDVGAR	*Homo sapiens, Sturnira hondurensis*	96	108	150.49	1
17	AVIGMTAGATGAFVGTPAEVALIR	Euteleostomi	112	135	171.47	1
18	*aspecific*					
19	*aspecific*					
20	GPAGVPGLLGDR	Eutheria	263	274	109.66	1
21	TSNFNAAISLK	Homininae	232	242	133.23	3
	AVNITSENLIDDVVSLIR	Homininae	413	430	156.6	1
22	AITQELPGLLG	Boreoeutheria	392	402	116.25	1
23	ETAVEAAPALGR	Catarrhini + *Tupaia chinensis*	248	259	131.5	1
24	DVFLGMFLYEYAR	Catarrhini + *Sturnira hondurensis* + *Ailuropoda melanoleuca*	156	168	122.51	1
25	*aspecific*					
26	EIGELYLPK	Homininae + *Sturnira hondurensis*	89	97	106.04	1
27	ALDLTQWLMVLK	Theria	961	972	160.75	1

**28**	*aspecific*					
**29**	DSYDSYATHNE	Tetrapoda	162	172	192.26	1
**30**	*aspecific*					
**31**	IEASLSVQMSR	Simiiformes	415	425	127.76	1
**32**	VLLSLFTTEMLMK	*Homo sapiens*	470	482	117.02	1
**33**	TEITVEDQLAR	Amniota	83	93	111.06	1
**34**	*aspecific*					
**35**	*aspecific*					
**36**	*aspecific*					
**37**	*DVSSIIVAFGYPFR*	Amniota	72	85	157.24	1

Table S4. Protein summary for sample 48 from Ilyinka, kurgan 4 burial 3 (Raw file ID: F10)

Protein no.	Leading Razor Protein	Species Specificity	No. of Identified Razor+ Unique Peptides	% Coverage (not razor)	No. of MS2 spectra (razor)
**1**	MBZ3879049.1 Keratin, type II cuticular Hb1	*Neosciurus carolinensis*	111	46.0	518
**2**	tr|A0A5E4CA63| IF rod domain-containing protein	*Marmota* sp. and *Ictidomys tridecemlineatus*	92	55.2	441
**3**	XP_026254549.1 keratin, type II cuticular Hb5 isoform X2	Boreoeutheria	26	46.8	64
**4**	XP_015351223.1 PREDICTED: keratin, type I cuticular Ha4	*Marmota marmota marmota* and *Ictidomys tridecemlineatus*	16	56.5	96
**5**	XP_040124725.1 keratin, type I cuticular Ha6	Rodentia	12	38.1	30
**6**	MBZ3879004.1 Keratin, type II cuticular Hb1	*Neosciurus carolinensis*	9	47.1	56
**7**	MBZ3886840.1 Keratin, type I cytoskeletal 39	*Neosciurus carolinensis*	6	13.4	6
**8**	MBZ3872749.1 Keratin, type II cuticular Hb5	Eutheria	3	18.9	5
**9**	XP_015348878.1 PREDICTED: keratin, type II cuticular Hb3	Marmotini, *Equus* sp., *Erinaceus europaeus, Odobenus rosmarus divergens,* and *Trichechus manatus latirostris*	2	45.9	20
**10**	XP_005321900.1 keratin, type I cuticular Ha2	Marmotini, *Lemur catta,* and *Microcebus murinus*	2	20.1	2

Table S5. Specific peptides from sample 48 from Ilyinka, kurgan 4 burial 3 (Raw file ID: F10)

Protein no.	Peptide	Species	Start position	End position	Score	No. of MS2
**1**	LETAVTQSEQQGEAALSDAR	Sciuridae and Feliformia	346	365	447.43	25
	TREEINELNR	Rodentia + *Ovis aries, Globicephala melas,* and *Loxodonta africana*	318	327	300.81	19
	VLHSHISDTSVIVK	*Neosciurus carolinensis, Dasypus novemcinctus, Choloepus didactylus, Chrysochloris asiatica, Elephantulus edwardii, Echinops telfairi,* and *Trichechus manatus latirostris*	249	262	282.61	50
**2**	IQERNQQQDPLV	Rodentia	88	99	143.96	1
	RTVNALEIELQAQHNLR	various but not *Ornithorhynchus anatinus*	277	293	282.59	18
	SEKETMQFLNDR	various but not Cricetinae nor *Ornithorhynchus anatinus*	55	66	210.15	2
	VRQLERENAELEAR	Marmotini, Cricetinae, *Ornithorhynchus anatinus*	74	87	218.98	4
	YSSQLSQVQCMITNVESQLAEIR	Eutheria (only rodents are *Marmota* sp. and *Ictidomys tridecemlineatus*; not *Ornithorhynchus anatinus*)	307	329	156.36	1
**3**	KSDLEANVEALVEESSFLK	Boreoeutheria	239	257	128.94	3
	SDLEANVEALVEESSFLKR	Boreoeutheria	240	258	242.10	2
**4**	PGYQSYFR	*Marmota marmota marmota, Ictidomys tridecemlineatus, Phycisphaerae bacterium* (marine water bacteria, not included in final species ID)	101	108	129.40	1
**5**	TVNSLEIELQAQQSMR	Rodentia + *Pipistrellus kuhlii*	310	325	142.10	1
	DALDSTLAETEAR	Rodentia + *Loxodonta africana*	326	338	167.12	3
**6**	VLHSHISDTSVVVK	*Neosciurus carolinensis, Erinaceus europaeus, Pteropus giganteus, Chrysochloris asiatica,* and *Trichechus manatus latirostris*	253	266	250.7	13
	TKEEMNELNR	Theria (but NOT *Erinaceus europaeus, Pteropus giganteus, Chrysochloris asiatica, Trichechus manatus latirostris*)	322	331	237.52	12
	LLEGEEQRLCEGI	Theria (but NOT *Erinaceus europaeus, Pteropus giganteus, Chrysochloris asiatica, Trichechus manatus latirostris*)	413	425	139.32	1
**7**	QLVEADANSLRK	*Neosciurus carolinensis* + Marmotini	166	177	110.08	1
	TLNALEVDLQAQHR	*Neosciurus carolinensis*	291	304	137.46	1
**8**	LTAEVENAKQQR	Eutheria	350	361	148.52	3
**9**	AQYDDIANR	*Marmotini, Equus sp., Erinaceus europaeus, Odobenus rosmarus divergens,* and *Trichechus manatus latirostris*	285	293	207.6	16
**10**	GSLENTLAETEAR	Marmotini, *Lemur catta,* and *Microcebus murinus*	339	351	248.05	1

Table S6. Suspected *S. vulgaris* peptides in sample 48 from Ilyinka, kurgan 4 burial 3 (Raw file ID: F10) and their Marmotini equivalents

Equivalent to	Suspected *S. vulgaris* Peptide	Blast results	Score	No. of MS2	Original Marmotini Protein Identifier	Original Marmotini Version Peptides	Original Marmotini version also found?	Notes
keratin, type I cuticular Ha5, keratin, type I cuticular Ha4, IF rod domain-containing protein	/	/	/	/	XP_026242668, XP_015351223, A0A5E4CA63	YSSQLSQMQCMISNVESQL**A**EIR	Yes, 1 PSM, poor spectra	
	MISNVESQL**S**EIR	*Dipodomys spectabilis*	199.19	4		MISNVESQL**A**EIR	No	
	ISNVESQL**S**EIR	*Dipodomys spectabilis*	286.03	3		ISNVESQL**A**EIR	No	
	SNVESQL**S**EIR	*Dipodomys spectabilis*	169.65	1		SNVESQL**A**EIR	No	
keratin, type I cuticular Ha4	SDLEAQVESL**R**EELLSLK	Carnivora + some rodents and rabbits	401.16	5	XP_015351223	SDLEAQVESL**K**EELLSLK	No	
	SDLEAQVESL**R**EELLSLKR	Canidae + *Dipodomys spectabilis* + *Mirounga angustirostris*	195.97	5		SDLEAQVESL**K**EELLSLKR	No	
	EAQVESL**R**EELLSLK	Carnivora + some rodents and rabbits	109.1	1		EAQVESL**K**EELLSLK	No	
	EAQVESL**R**EELLSLKR	Canidae + *Dipodomys spectabilis* + *Mirounga angustirostris*	108.49	1		EAQVESL**K**EELLSLKR	No	
	QVESL**R**EELLSLK	Carnivora + some rodents and rabbits	112.95	1		QVESL**K**EELLSLK	No	
	QVESL**R**EELLSLKR	Canidae + *Dipodomys spectabilis* + *Mirounga angustirostris*	109.07	1		QVESL**K**EELLSLKR	No	
keratin, type I cuticular Ha1, keratin, type I cuticular Ha4	IQER**S**QQQ	several, including human and bacteria	122.19	1	XP_015351222, XP_015351223	IQER**N**QQQ	Yes, 2 PSMs, spectra ok but lower score (106.91)	Both versions might be present due to sequence similarity in two different keratins
	IQER**S**QQQD	Rodentia + *Lipotes vexillifer* + *Ranitomeya imitator*	127.94	1		IQER**N**QQQD	Yes, 2 PSMs, spectra ok, score 139.02	
	**S**QQQDPLVCPSYQ**A**YFR	No match	161.76	1		**N**QQQDPLVCPSYQ**S**YFR	No	
	PSYQ**A**YFR	No match	168.45	1		PSYQ**S**YFR	No	
	QLER**D**NAELEAR	*Orycteropus afer afer*, *Mesocricetus auratus*	381.56	9		QLER**E**NAELEAR	Yes, 19 PSMs, spectra ok, score 351.94	
	**D**NAELEAR	Bacteria + various	208.22	3		**E**NAELEAR	Yes, 12 PSMs, spectra ok, score 205.77	

Table S7. Protein summary for sample 49 from Vil'na Ukraina 4, kurgan 22 burial 1 (Raw file ID: F11)

Protein no.	Leading Razor Protein	Species Specificity	No. of Identified Razor + Unique Peptides	% Coverage (not razor)	No. of MS2 spectra (razor)
**1**	XP_029784577.1 collagen alpha-1(I) chain	Feliformia	373	64.8	863
**2**	XP_040328585.1 collagen alpha-2(I) chain	Felidae*	324	65.5	628
**3**	sp|P02465|Collagen alpha-2(I) chain	*Bos* sp. or *Bison bison bison***	76	62.1	144
**4**	XP_040343968.1 keratin, type II cuticular Hb1-like	Boreoeutheria***	63	43.6	64
**5**	tr|A0A485MV79| keratin, type I curticular Ha3-I	Felidae	59	63.4	173
**6**	JACGTZ010000853_7a hypothetical collagen alpha-1(III) chain from Carcal Carcal	Felidae (not *Panthera* sp.)	30	28.4	53
**7**	sp|P02453| Collagen alpha-1(I) chain	Laurasiatheria (not Feliformia, probably Bovinae)	20	62.4	63
**8**	XP_040343979.1 keratin, type II cuticular Hb5	Laurasiatheria	15	42.4	34
**9**	XP_046922113.1 keratin, type II cuticular Hb6	Felidae†	13	50.9	48
**10**	XP_026904859.1 keratin, type I cuticular Ha3-I	*Acinonyx jubatus, Loxodonta africana*	8	60.9	18
**11**	tr|A0A6J1XHR0| keratin, type II cuticular Hb1-like	*Acinonyx jubatus*	6	42.8	42
**12**	XP_039112760.1 keratin, type I cuticular Ha5	Laurasiatheria	3	18.9	5
**13**	tr|A0A6I9ZLE5| keratin, type I cuticular Ha3-II-like	*Acinonyx jubatus*	2	52.7	5

* - Felidae can be narrowed down to the only matching "Old World" felids with sequences available: *Felis catus, Lynx pardinus, Acinonyx jubatus*. Also found matches in DNA sequences of *Felis chau/nigripes* but not Caracal *caracal*

** - *Bison bison bison* is from North America, and can therefore be disregarded

*** - Only "Old World" Feliforma: *Prionailurus viverrinus/bengalensis, Lynx pardinus, Panthera* sp., *Suricata suricatta*

† - Felidae can be narrowed down to the only matching "Old World" felids with sequences available: *Lynx pardinus, Felis catus, Prionailurus bengalensis/viverrinus*

Table S8. Specific peptides from sample 49 from Vil'na Ukraina 4, kurgan 22 burial 1 (Raw file ID: F11)

Protein No.	Peptide	Species	Start position	End position	Score	No. of MS2
**1**	GPPGAAGSPGK	Feliformia	1138	1148	131.50	2
	GPPGAAGSPGKD	Feliformia	1138	1149	117.28	1
**2**	GLPGVSGSVGEPGPLGIS	Laurasiatheria ("Old World" felids: *Felis catus, Lynx pardinus, Acinonyx jubatus*)	884	901	166.29	1
	GLPGVSGSVGEPGPLGISG	Laurasiatheria ("Old World" felids: *Felis catus, Lynx pardinus, Acinonyx jubatus*)	884	902	205.56	1
	GLPGVSGSVGEPGPLGISGP	Laurasiatheria ("Old World" felids: *Felis catus, Lynx pardinus, Acinonyx jubatus*)	884	903	224.66	1
	GLPGVSGSVGEPGPLGISGPPG	Laurasiatheria ("Old World" felids: *Felis catus, Lynx pardinus, Acinonyx jubatus*)	884	905	283.44	1
	GLPGVSGSVGEPGPLGISGPPGA	Laurasiatheria ("Old World" felids: *Felis catus, Lynx pardinus, Acinonyx jubatus*)	884	906	169.55	1
	GLPGVSGSVGEPGPLGISGPPGAR	Laurasiatheria ("Old World" felids: *Felis catus, Lynx pardinus, Acinonyx jubatus*)	884	907	412.21	15
	LPGVSGSVGEPGPLGISGPPGAR	Laurasiatheria ("Old World" felids: *Felis catus, Lynx pardinus, Acinonyx jubatus*)	885	907	231.71	1
	PGVSGSVGEPGPLGISGPPGAR	Laurasiatheria ("Old World" felids: *Felis catus, Lynx pardinus, Acinonyx jubatus*)	886	907	275.83	3
	PGVSGSVGEPGPLGISGPPGARG	Laurasiatheria ("Old World" felids: *Felis catus, Lynx pardinus, Acinonyx jubatus*)	886	908	136.38	1
	GVSGSVGEPGPLGISGPPGAR	Laurasiatheria ("Old World" felids: *Felis catus, Lynx pardinus, Acinonyx jubatus*)	887	907	178.22	1
	SGSVGEPGPLGISGPPGAR	Laurasiatheria ("Old World" felids: *Felis catus, Lynx pardinus, Acinonyx jubatus*)	889	907	245.03	2
	GPSGAVGAPGVN	Felidae ("Old World": *Felis catus, Prionailurus bengalensis/viverrinus, Lynx pardinus, Acinonyx jubatus, Panthera tigris/leo/pardus*)	908	919	144.90	3
	GPSGAVGAPGVNGAPG	Felidae ("Old World": *Felis catus, Prionailurus bengalensis/viverrinus, Lynx pardinus, Acinonyx jubatus, Panthera tigris/leo/pardus*)	908	923	134.57	1
	GPSGAVGAPGVNGAPGEA	Felidae ("Old World": *Felis catus, Prionailurus bengalensis/viverrinus, Lynx pardinus, Acinonyx jubatus, Panthera tigris/leo/pardus*)	908	925	208.51	2
	GPSGAVGAPGVNGAPGEAG	Felidae ("Old World": *Felis catus, Prionailurus bengalensis/viverrinus, Lynx pardinus, Acinonyx jubatus, Panthera tigris/leo/pardus*)	908	926	203.69	3
	GPSGAVGAPGVNGAPGEAGR	Felidae ("Old World": *Felis catus, Prionailurus bengalensis/viverrinus, Lynx pardinus, Acinonyx jubatus, Panthera tigris/leo/pardus*)	908	927	365.19	3
	GPSGAVGAPGVNGAPGEAGRD	Felidae ("Old World": *Felis catus, Prionailurus bengalensis/viverrinus, Lynx pardinus, Acinonyx jubatus, Panthera tigris/leo/pardus*)	908	928	335.91	5
	GPSGAVGAPGVNGAPGEAGRDG	Felidae ("Old World": *Felis catus, Prionailurus bengalensis/viverrinus, Lynx pardinus, Acinonyx jubatus, Panthera tigris/leo/pardus*)	908	929	130.05	2
	GPSGAVGAPGVNGAPGEAGRDGN	Felidae ("Old World": *Felis catus, Prionailurus bengalensis/viverrinus, Lynx pardinus, Acinonyx jubatus, Panthera tigris/leo/pardus*)	908	930	338.50	2
	SGAVGAPGVNGAPGEAGR	Felidae ("Old World": *Felis catus, Prionailurus bengalensis/viverrinus, Lynx pardinus, Acinonyx jubatus, Panthera tigris/leo/pardus*)	910	927	208.35	3

	GPAGPSGPMGK	Felidae ("Old World": *Felis catus, Prionailurus bengalensis/viverrinus, Lynx pardinus, Acinonyx jubatus, Panthera tigris/leo/pardus*)	1055	1065	130.66	3
	GPAGPSGPMGKDGR	Felidae ("Old World": *Felis catus, Prionailurus bengalensis/viverrinus, Lynx pardinus, Acinonyx jubatus, Panthera tigris/leo/pardus*)	1055	1068	124.12	3
**3**	GAPGAIGAPGPAGAN	*Bos* sp., *Bison bison bison, Toxodon* sp.	674	688	132.84	1
	GAPGAIGAPGPAGANGDR	*Bos* sp., *Bison bison bison, Toxodon* sp.	674	691	190.1	2
	SGETGASGPPGFVGEK	*Bos* sp., *Bison bison bison, Balaenoptera acutorostrata scammoni, Mus musculus*	829	844	241.35	1
	IGQPGAVGPAGIR	Bovinae	1066	1078	192.91	4
**4**	KSDLEANAEALTEEI**N**FLR	Feliformia + some marsupials, **but** deamidated N is also several Boreoeutheria	284	302	405.66	16
	KSDLEANAEALTEEI**N**FLRR	Felidae + *Suricata suricatta* (Only "Old World" Felidae: *Prionailurus viverrinus/bengalensis, Lynx pardinus, Panthera* sp.) **but** deamidated N is also several Boreoeutheria	284	303	300.64	38
	SDLEANAEALTEEI**N**FLR	Feliformia + some marsupials, **but** deamidated N is also several Boreoeutheria	285	302	436.90	5
	SDLEANAEALTEEI**N**FLRR	Felidae + *Suricata suricatta* (Only "Old World" Felidae: *Prionailurus viverrinus/bengalensis, Lynx pardinus, Panthera* sp.) **but** deamidated N is also several Boreoeutheria	285	303	288.16	4
	NAEALTEEI**N**FLRR	Felidae + *Suricata suricatta* (Only "Old World" Felidae: *Prionailurus viverrinus/bengalensis, Lynx pardinus, Panthera* sp.) **but** deamidated N is also several Boreoeutheria	290	303	97.27	1
**5**	VRQLERENAELETR	Felidae + *Molossus molossus*	74	87	196.18	4
	QLERENAELETR	Felidae + *Molossus molossus*	76	87	289.71	6
	SDLEAQVESLREELLSLKK	Feliformia + *Chinchilla lanigera, Ochotona curzoniae, Oryctolagus cuniculus, Octodon degus*	174	192	85.50	1
**6**	GAPGEPGRDGVPGGPGIR	Feliformia	519	536	138.87	4
	PGEPGRDGVPGGPGIR	Feliformia	521	536	163.8	1
	GPPGALGPAGPR	Feliformia (not *Panthera* sp.)	684	695	153.04	1
	GAPGPQGLPGLAGAAGEPGR	Felidae (not *Panthera* sp.)	993	1012	111.06	1
**7**	GAPGADGPAGAPGTPGPQGIAGQR	Laurasiatheria	934	957	390.77	19
**8**	LYDEELQVLH	Laurasiatheria	259	268	126.52	2
	LYDEELQVLHA	Laurasiatheria	259	269	151.5	2
	LYDEELQVLHAHIS	Laurasiatheria	259	272	94.728	1
	LYDEELQVLHAHISD	Laurasiatheria	259	273	117.48	1
	LYDEELQVLHAHISDTSVIVK	Laurasiatheria	259	279	143.15	1
**9**	KSDLEANAEALIEEINFLR	Felidae ("Old World": *Lynx pardinus, Felis catus, Prionailurus bengalensis/viverrinus, Panthera tigris*) + *Gracilinanus agilis*	222	240	370.86	16
	KSDLEANAEALIEEINFLRR	Felidae ("Old World": *Lynx pardinus, Felis catus, Prionailurus*	222	241	85.808	1

		bengalensis/viverrinus, Panthera tigris)				
	SDLEANAEALIEEINFLR	Felidae ("Old World": *Lynx pardinus, Felis catus, Prionailurus bengalensis/viverrinus, Panthera tigris*) + *Gracilinanus agilis*	223	240	444.59	5
	AEALIEEINFLR	Felidae ("Old World": *Lynx pardinus, Felis catus, Prionailurus bengalensis/viverrinus, Panthera tigris*) + *Gracilinanus agilis*	229	240	188.04	1
	CQNTKLETAVTQSEQQGEAALSDAR	Feliformia ("Old World": *Lynx pardinus, Felis catus, Prionailurus bengalensis/viverrinus, Acinonyx jubatus, Hyaena hyaena*) + Sciuridae	341	365	186.65	1
	LETAVTQSEQQGEAALSDAR	Feliformia ("Old World": *Lynx pardinus, Felis catus, Prionailurus bengalensis/viverrinus, Acinonyx jubatus, Hyaena hyaena*) + Sciuridae	346	365	329.4	8
**10**	YSAQLSQVQYMITNVESQLAEIR	*Acinonyx jubatus, Loxodonta africana*	307	329	358.31	3
**11**	KSDLEANTEALTEEINFLR	*Acinonyx jubatus*	283	301	165.17	1
	SDLEANTEALTEEINFLR	*Acinonyx jubatus*	284	301	356.88	4
**12**	TKYETEVSMR	Laurasiatheria	184	193	131.83	2
**13**	TVNALEIELQAQHK	*Acinonyx jubatus, Trichechus manatus latirostris, Ornithorhynchus anatinus, Tachyglossus aculeatus*	166	179	194.39	4
	YSAQLGQVQCMITNVEAQLAEIR	*Acinonyx jubatus*	195	217	138.81	1

Text. Detailed LC-MS/MS method and discussion of results

Mass spectrometry analysis (LC-MS/MS)

For mass spectrometry analysis, ZooMS eluates were provided at approximately 0.4 µg/µL in 50% acetonitrile (ACN). Of this, 3 µL of each sample were put into separate wells of a 96-well MS plate. Samples were then vacuum-centrifuged to remove the ACN. Samples were then resuspended in 12 µL of 5% ACN 0.1 % trifluoroacetic acid (TFA).

Samples were then analysed by liquid chromatography-tandem mass spectrometry (LC-MS/MS) based on previously published protocols for palaeoproteomic samples [1,2]. Samples were separated on a 15 cm column (75 µm inner diameter) in-house laser pulled and packed with 1.9 µm C18 beads (Dr. Maisch, Germany) on an EASY-nLC 1200 (Proxeon, Odense, Denmark) connected to an Orbitrap Exploris 480 (Thermo Scientific, Bremen, Germany). 5 µL of sample was injected. Buffer A was milliQ water and the peptides were separated with increasing buffer B (80% ACN and 0.1% formic acid) with a 77 min gradient, going from 5% to 30% in 50 min, 30% to 45% in 10 min, 45% to 80% in 2 min, held at 80% for 5 min before dropping back down to 5% in 5 min and held for 5 min. Flow rate was 250 nL/min. The column temperature was maintained at 40°C using an integrated column oven.

Full scan mass spectra (MS1) were recorded by the Orbitrap Exploris 480 at a resolution of 120,000 at m/z 200 over the m/z range 350–1400 with a target value of 3e6 and a maximum injection time of 25 ms. Spray voltage was set to 2 kV, S-lens RF level was at 40 %, and the heated capillary at 275°C. HCD-generated product ions (MS2) were recorded in data dependent top 10 mode with a maximum ion injection time set to 118 ms and a target value set to 2e5 and recorded at a resolution of 60,000. Normalised collision energy was set at 30% and the isolation window was 1.2 m/z with the dynamic exclusion set to 20 s.

Additionally, to hinder cross-contamination, a wash-blank method using 5% ACN 0.1 % TFA was run in between each sample.

Data analysis

Thermo .raw files generated by the mass spectrometer were then searched using MaxQuant [v.1.6.3.4; 3] for interpretation of spectra. Files were first run against the Swissprot Database (downloaded from Uniprot 09/06/2020). From this initial search, following iterative searches were made to narrow down the database to the relevant proteins from the relevant species. The databases used to narrow down identifications after indications from Swissprot varied between samples. For sample MG21 (21 from Bulhakovo, kurgan 5, burial 2), after the determination of hominin origin from the Swissprot search, the human proteome from Uniprot (downloaded 25/11/20) was searched. In an attempt to improve identifications, a database was made solely of the protein hits from this search (downloaded 05/01/2022) and searched again for final identifications.

For the apparent non-hominin samples (F10/F11), a database of mammalian collagens 1-3 and keratins was made from both Uniprot and NCBI (downloaded 31/05/21) after the Swissprot searches. The hits from this database led to further narrowing of the databases. For

sample F10 (48 from Ilyinka, kurgan 4 burial 3), a database was composed first of Rodentia collagen 1-3 and keratin sequences from both Uniprot and NCBI (downloaded 09/08/2021), which indicated Sciuridae origin. A database of Sciuridae collagen 1-3 and keratin sequences from both Uniprot and NCBI (downloaded 09/11/2021), with additional predicted keratin sequence fragments from S. vulgaris (Brandt et al. 2022) was then used for final identification. For sample F11 (Vil'na Ukraina 4, kurgan 22 burial 1), a database was composed first of Carnivora collagen 1-3 and keratin sequences from both Uniprot and NCBI (downloaded 07/02/2022), which indicated Felidae origin. A database of Felidae collagen 1-3 and keratin sequences from both Uniprot and NCBI (downloaded 12/07/2022), with additional hypothetical keratin and collagen sequences created from tBLASTn searching of the genomes from Felis chaus (jungle cat), Felis nigripes (Black-footed cat) and Caracal caracal (caracal) based on the proteins recovered, was used for final identification.

The initial Swissprot search was performed with tryptic digestion with a minimum length of 7 amino acids. Default settings of 20 ppm for the first search and 4.5 ppm for the final search were used, a fragment mass tolerance of 20 ppm, and the peptide spectrum match (PSM) and protein false-discovery rate was set to 1%, with a minimum required Andromeda score of 60 for all peptides. carbamidomethylation of cysteine was set as a fixed post-translational modification (PTM), and glutamine and asparagine deamidation, oxidation of methionine, proline hydroxylation, and pyroglutamic acid from glutamic and aspartic acids were all considered as possible variable modifications. Proteins were considered present if at least two (non completely overlapping) peptides were recovered. To improve recovery of peptides damaged by degradation processes, subsequent searches were set to semi-tryptic digestion, with a minimum peptide length of 8 amino acids and a maximum of 25. In addition, sample F11 was searched with carbamidomethylation of cysteine as a variable modification, as well as allowing mono-, di-, and tri-oxidation of cysteine. As oxidation of cysteine can occur during keratin decay [4], this was done to try and improve peptide identifications.

Common laboratory contaminants (for example, proteinaceous laboratory reagents and human skin keratins) were identified and removed from further examination from the database provided by MaxQuant.

Identified peptides were also searched against all known protein sequences by pBLAST [5] to determine their species specificity. Peptides matching to soil bacteria were discounted as possible contamination. All spectra of peptides indicative of species were also examined manually as a form of quality control to make sure identifications were as secure as possible. Spectra were graphically visualised using the Interactive Peptide Spectral Annotator [6] (Figures S2).

Supplementary discussion of LC-MS/MS Results

Species Identification and Current Databases

It has recently been emphasised that current publicly available sequences of collagens, but especially keratins, useful for species identification of archaeological furs, leather, and textiles are lacking [1]. Sequences important for biological research are often prioritised, which often means many wild species have few, or no, protein sequences available to

compare against our samples. Therefore, for samples 48 and 49, the exact species cannot be determined with 100% certainty as they are probably not available in current databases.

Sample 48 was determined to be Sciuridae, as it matched to many species in this family, depending on the protein. Several proteins were specific to the grey squirrel (*Neosciurus carolinensis*), native to North America—and therefore unlikely to be the actual species. Official European red squirrel (*Sciurus vulgaris*) sequences are not available, but possible variants have been published by Brandt et al. [1]. Five of these variants were detected in this sample, indicating that red squirrel is a very probable identification.

Unfortunately, sample 49 has the same problem, with the proteins in the sample matching several species in the Felidae family, with no species matching all proteins. Therefore, it is likely that this sample is a currently unsequenced Euroasian felid that combines elements of all of these protein sequences. DNA is available for the species *Felis chaus* (jungle cat), *Felis nigripes* (Black-footed cat) and *Caracal caracal* (caracal), which could be searched by tBLASTn for the proteins/peptides recovered which created hypothetical variants. However, this did not help secure an identification except to hypothetically eliminate the identification of the caracal for collagen 1 alpha 2 (Table S7). There are several felid species that could not be examined, such as the Sand cat (*Felis margarita*) or Pallas's cat/manul (*Otocolobus manul*) that are native to the lands of ancient Scythia which could be likely source species.

Bos Contamination in sample 49

Besides the collagens and keratins related to felids, sample 49 also provided evidence of collagen 1 alpha 1 (COL1A1) and 2 (COL1A2) from non-felid species. Peptides from COL1A1 allowed species identification of *Bos* sp. (cattle species) and *Bison bison bison* (bison). Since bison are native to North America, they are not likely to be identified in this context. Therefore, it is most likely from a *Bos* sp. Peptides of COL1A2 not matching felids were also identified, that also match bovids and are likely also from the same source. Since it is impossible that the fur and skin come from different species, this is likely some form of contamination. Bovine collagens could come from a glue made from cattle bones, however, there is no indication that this is present. It could be from an original application during the fur's use, although there is no visual evidence of this. There is no record of its use in a conservation process either, and any conservational glue would likely be from a synthetic source in this context. Therefore, the most likely source of these collagens is from another textile from the grave context. These textiles were found on top of each other and were hard to tease apart in some instances. Therefore, it is possible that a miniscule part of leather from another piece of clothing could have contaminated this sample.

Human identification of sample 21

The species identification of sample 21 is Homininae (probably *Homo sapiens*). It was already suspected to be human (*Homo sapiens*) based on the identification of 'Primate?' by ZooMS. In the search of Swissprot (which contains all the manually reviewed proteins, not those that have been only predicted), this sample matched best with human proteins. A search of all known collagens from Uniprot and NCBI also identified best with human sequences. Therefore, a semi tryptic search of the human proteome was conducted. pBLAST

investigation of the peptides did detect some human specific proteins, however, the proteins with the most peptides and MS/MS spectra could at best be narrowed down to Homininae (Great Apes). Since human keratin is a ubiquitous contaminant from dust and human handling, it cannot be used for species identification and these matches were disregarded. Collagens and muscle proteins such as myosins are less likely to be caused by contamination. These were the best represented proteins and therefore could provide the best evidence for species identification. The two *Homo sapiens* specific peptides were only found in less well-represented proteins, which are not necessarily specific to skin. These proteins must be viewed with caution, as the mass spectrometry facility also contains laboratories that work extensively with human cell lines for biological research and therefore could be contamination from the analysis environment. It is difficult to discard this contamination possibility without a blank sample extraction (as the ZooMS extractions did not include a laboratory blank), however, it should be noted that these proteins did not appear to be recovered in sample 48 and 49 (processed at the same time) by the Swissprot run, nor a Panthera proteome run for F11 (data not shown).

In regards to sample 21 potentially being another great ape species, this does not seem likely given the context, although technically still possible. To investigate this, a search was performed by PEAKS SPIDER [v7; 7](data not shown) that allows for the detection of single amino acid polymorphisms (SAPs). No amino acid variants from the human sequences were confidently detected, which is indicative of genuinely human proteins. Although, it could mean that those peptides were just not recovered. Therefore, this sample is conservatively identified as Homininae, and only probably as *Homo sapiens*.

SI References

1. Brandt LØ, Taurozzi AJ, Mackie M, Sinding M-HS, Vieira FG, Schmidt AL, et al. Palaeoproteomics identifies beaver fur in Danish high-status Viking Age burials - direct evidence of fur trade. PLoS One. 2022;17: e0270040.

2. Mackie M, Rüther P, Samodova D, Di Gianvincenzo F, Granzotto C, Lyon D, et al. Palaeoproteomic Profiling of Conservation Layers on a 14th Century Italian Wall Painting. Angew Chem Int Ed Engl. 2018;57: 7369–7374.

3. Cox J, Mann M. MaxQuant enables high peptide identification rates, individualized p.p.b.-range mass accuracies and proteome-wide protein quantification. Nature Biotechnology. 2008. pp. 1367–1372. doi:10.1038/nbt.1511

4. Solazzo C, Dyer JM, Clerens S, Plowman J, Peacock EE, Collins MJ. Proteomic evaluation of the biodegradation of wool fabrics in experimental burials. Int Biodeterior Biodegradation. 2013;80: 48–59.

5. Altschul SF, Gish W, Miller W, Myers EW, Lipman DJ. Basic local alignment search tool. J Mol Biol. 1990;215: 403–410.

6. Brademan DR, Riley NM, Kwiecien NW, Coon JJ. Interactive Peptide Spectral Annotator: A Versatile Web-based Tool for Proteomic Applications. Mol Cell Proteomics. 2019;18: S193–S201.

7. Ma B, Zhang K, Hendrie C, Liang C, Li M, Doherty-Kirby A, et al. PEAKS: powerful

software for peptide de novo sequencing by tandem mass spectrometry. Rapid Commun Mass Spectrom. 2003;17: 2337–2342.
