# Supplementary material for: Human and animal skin identified by palaeoproteomics in Scythian leather objects from Ukraine
Source: PLoS One. 2023 Dec 13;18(12):e0294129. doi: 10.1371/journal.pone.0294129 (PMC10718408; doi:10.1371/journal.pone.0294129)
Supplement: S2 File — (PDF) [file pone.0294129.s002.pdf]

SUPPORTING INFORMATION 2

Human and animal skin identified by palaeoproteomics in Scythian leather objects from Ukraine

Authors

Luise Ø. Brandt[1], Meaghan Mackie[1, 2], Marina Daragan[3], Matthew, J. Collins[1,4], Margarita Gleba[5]

Affiliations

[1] The Globe Institute, University of Copenhagen, Øster Farimagsgade 5, Bygning 7, 1353 Copenhagen K, Denmark

[2] Novo Nordisk Foundation Center for Protein Research, University of Copenhagen, Blegdamsvej 3b, 2200 Copenhagen N, Denmark

[3] Institute of Archaeology of the National Academy of Sciences of Ukraine, Volodymyr Ivasyuk Avenue 12, 04210 Kiev, Ukraine

[4] University of Cambridge, McDonald Institute for Archaeological Research, 2.8 Henry Wellcome Building, Fitzwilliam St, Cambridge CB2 1QH, UK

[5] Dipartimento dei Beni Culturali, Università degli Studi di Padova, Piazza Capitaniato 7, 35139 Padova, Italy

LC-MS/MS Spectra relevant for Species Identification

Lower case letters indicate post translational modifications (n/q is deamidation; m/p is mono-oxidation or hydroxyproline; c is trioxidation or carbamidomethylation; e is pyroglutamic acid). Spectra were visualised using the Interactive Peptide Spectral Annotator (Brademan et al., 2019).

A. ***Sample F10 (a-aa)***

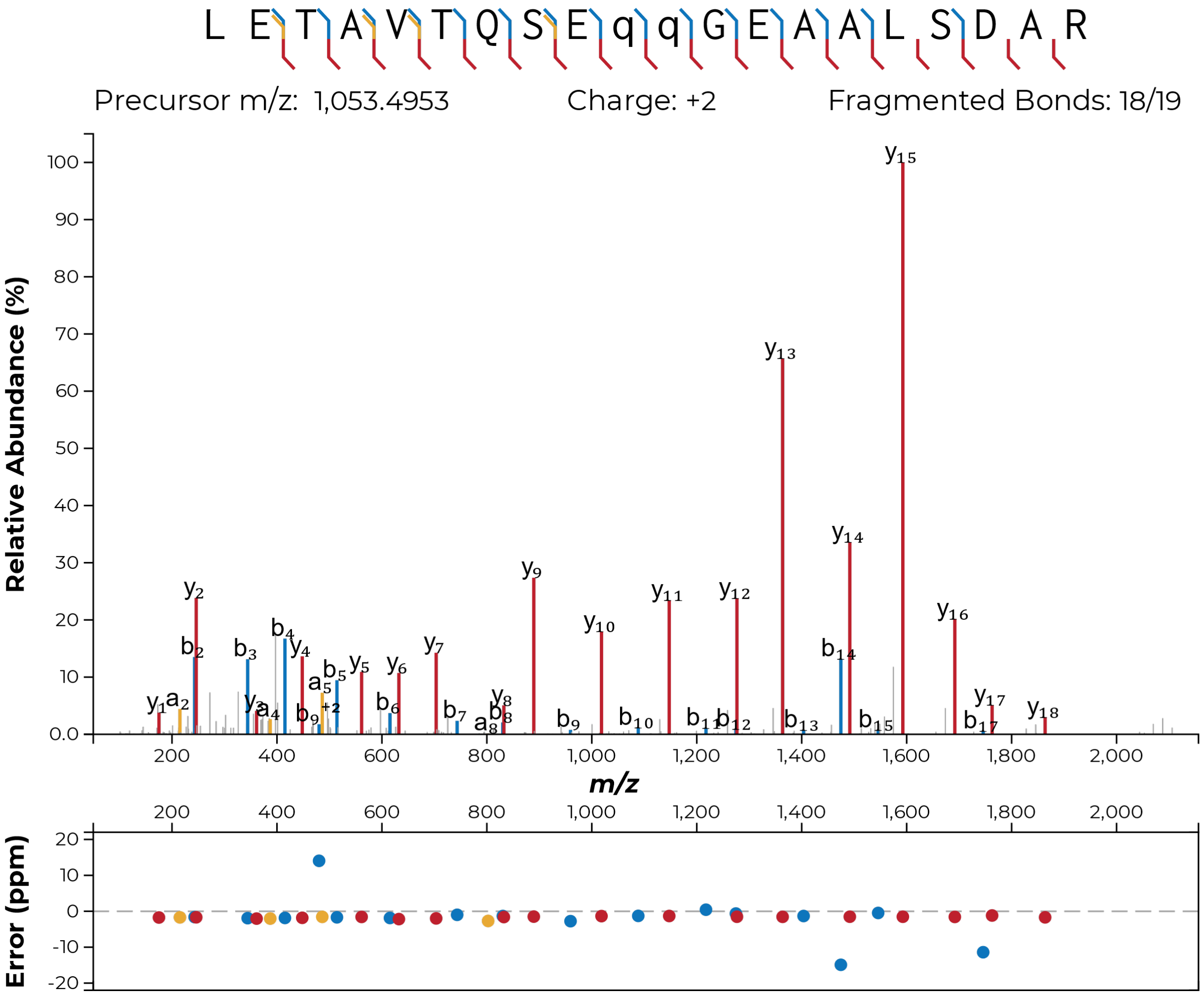

a MBZ3879049 LETAVTQSEQQGEAALSDAR scan 16718 score 447; Sciurdae and Feliformia

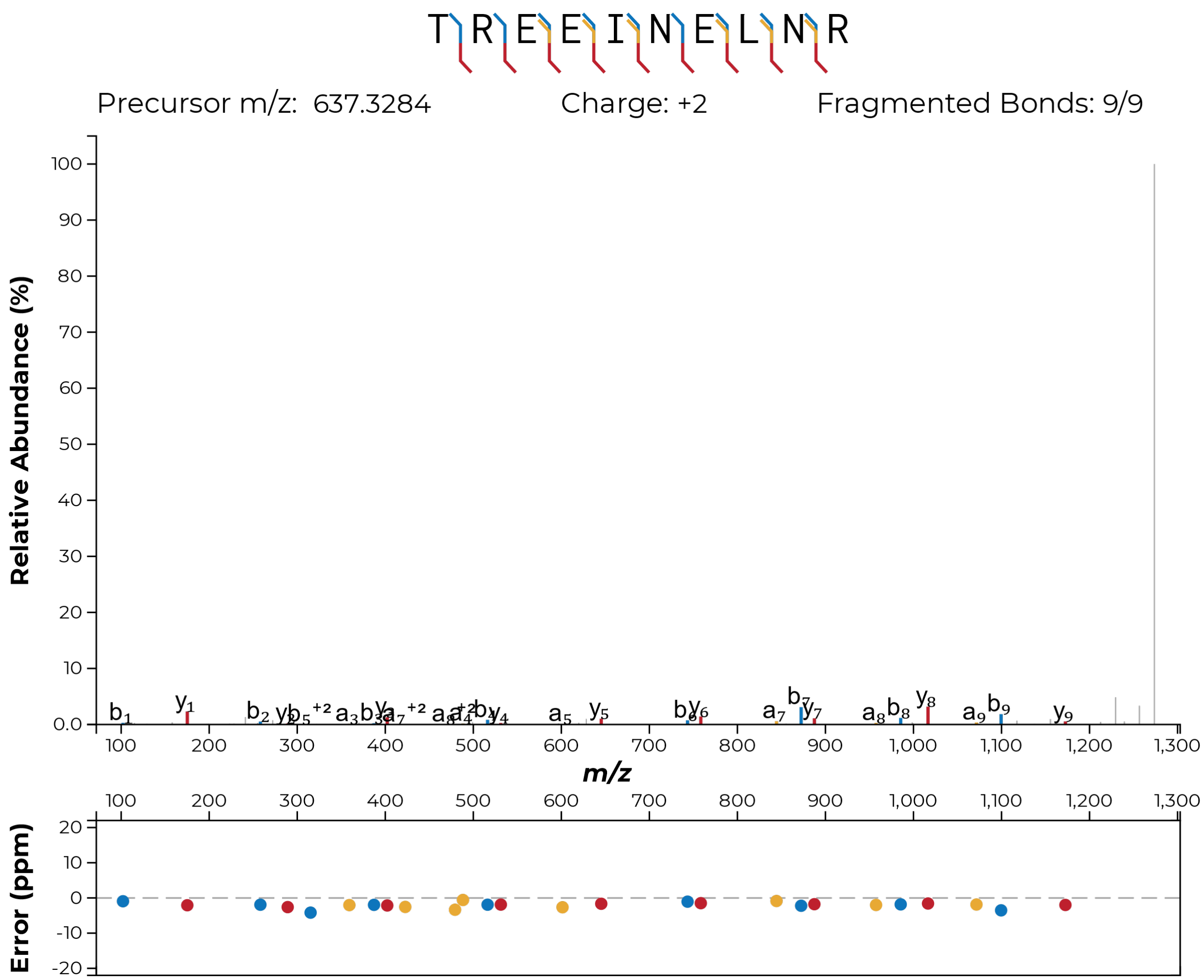

b MBZ3879049 TREEINELNR scan 7170 score 300; Rodentia + Ovis aries, Globicephala melas, and Loxodonta africana

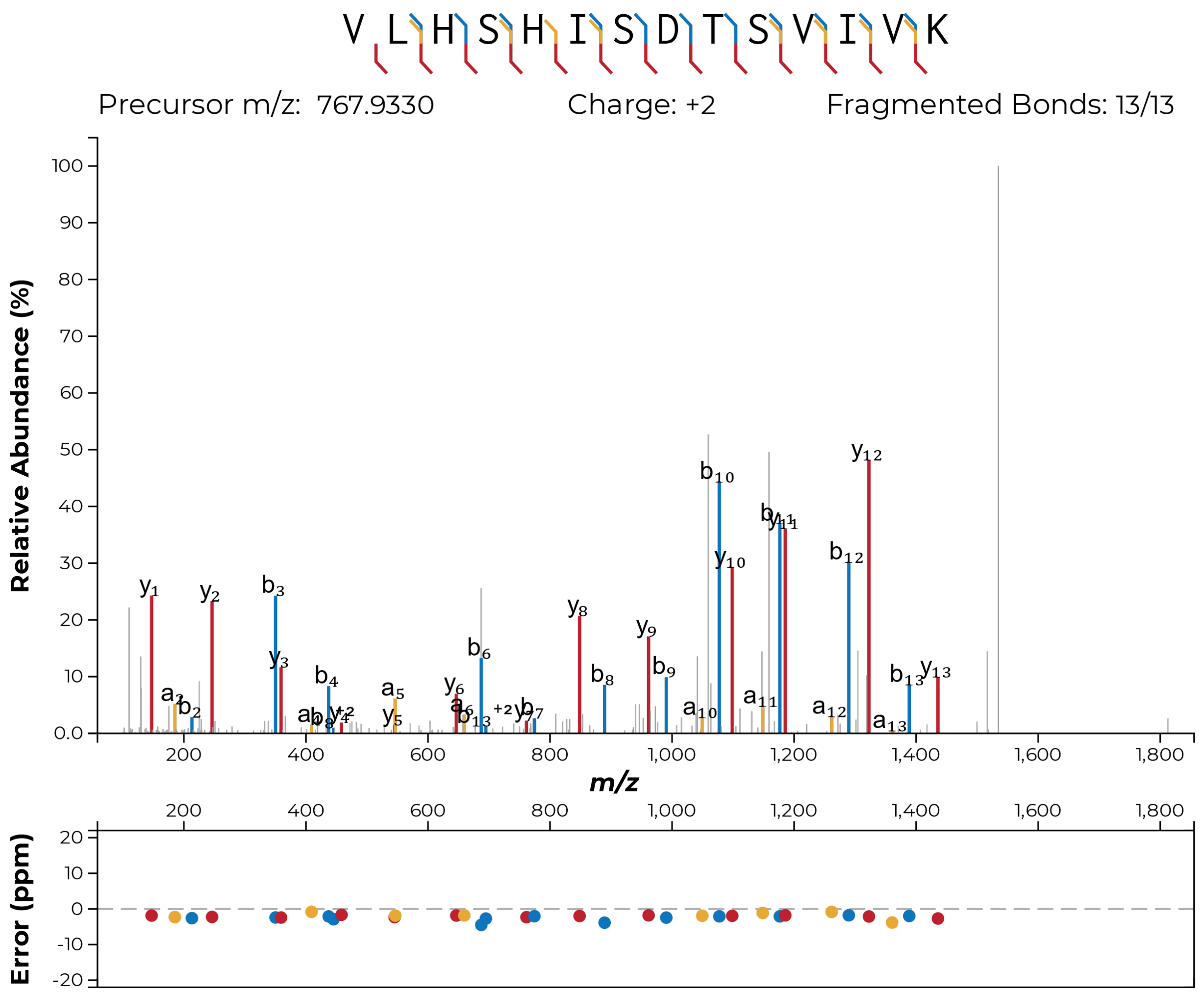

c *MBZ3879049 VLHSHISDTSVIVK scan 12109 score 275; Neosciurus carolinensis, Dasypus novemcinctus, Choloepus didactylus, Chrysochloris asiatica, Elephantulus edwardii, Echinops telfairi, and Trichechus manatus latirostris*

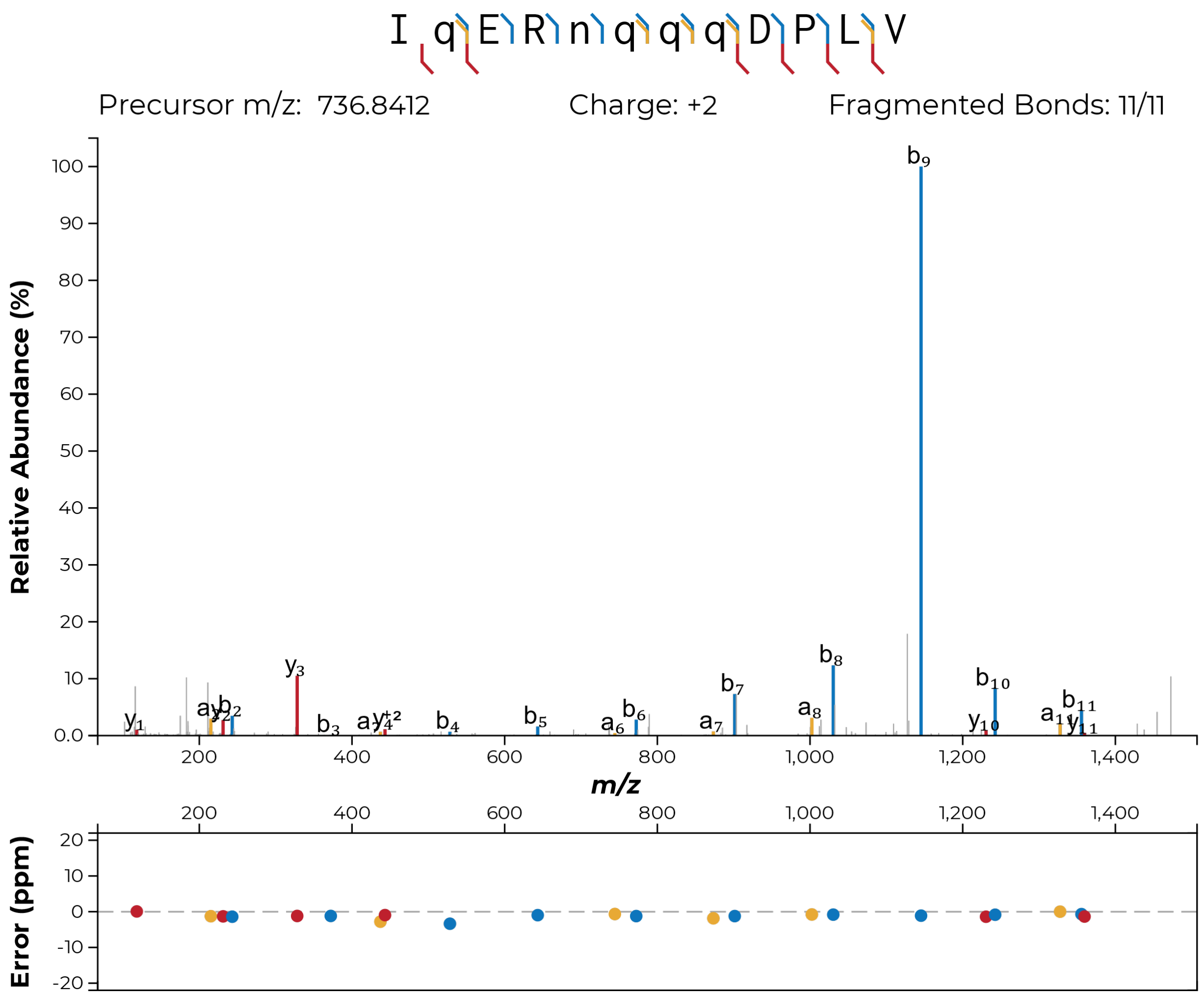

d A0A5E4CA63 IQERNQQQDPLV scan 12523 score 143; Rodentia

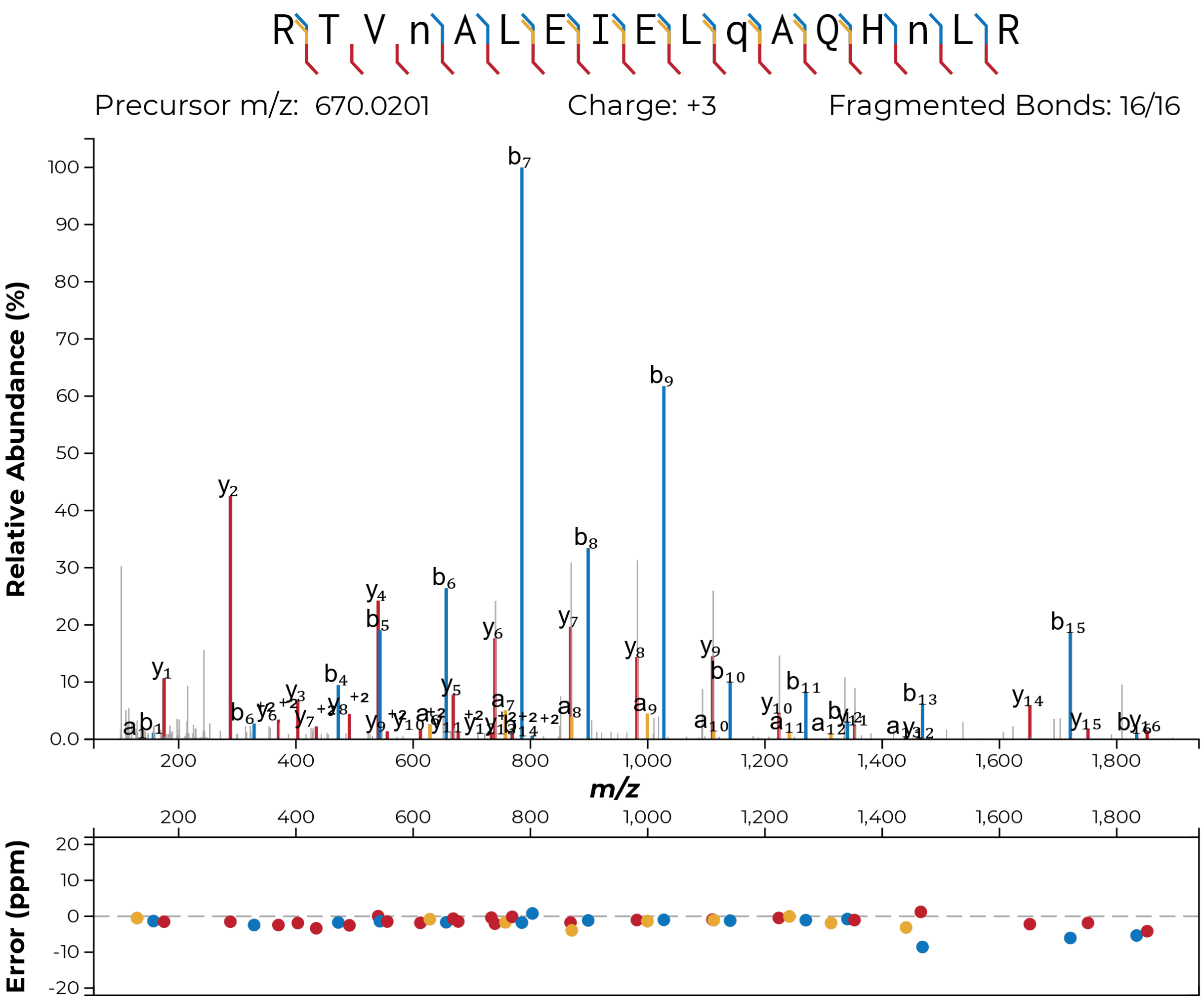

e A0A5E4CA63 RTVNALEIELQAQHNLR scan 20535 score 282; various but not Ornithorhynchus anatinus

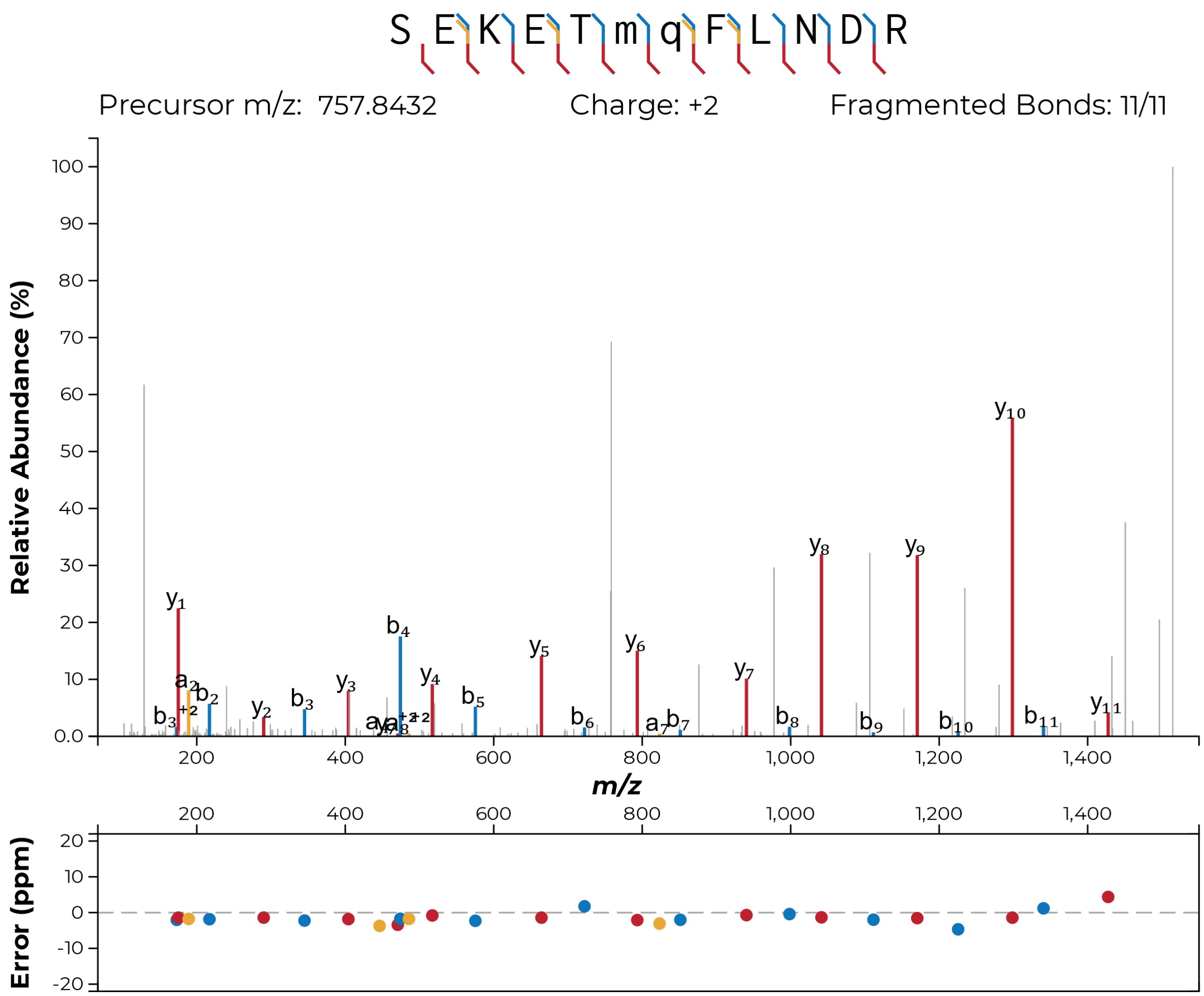

f A0A5E4CA63 SEKETMQFLNDR scan 9430 score 210; various but not Cricetinae nor Ornithorhynchus anatinus

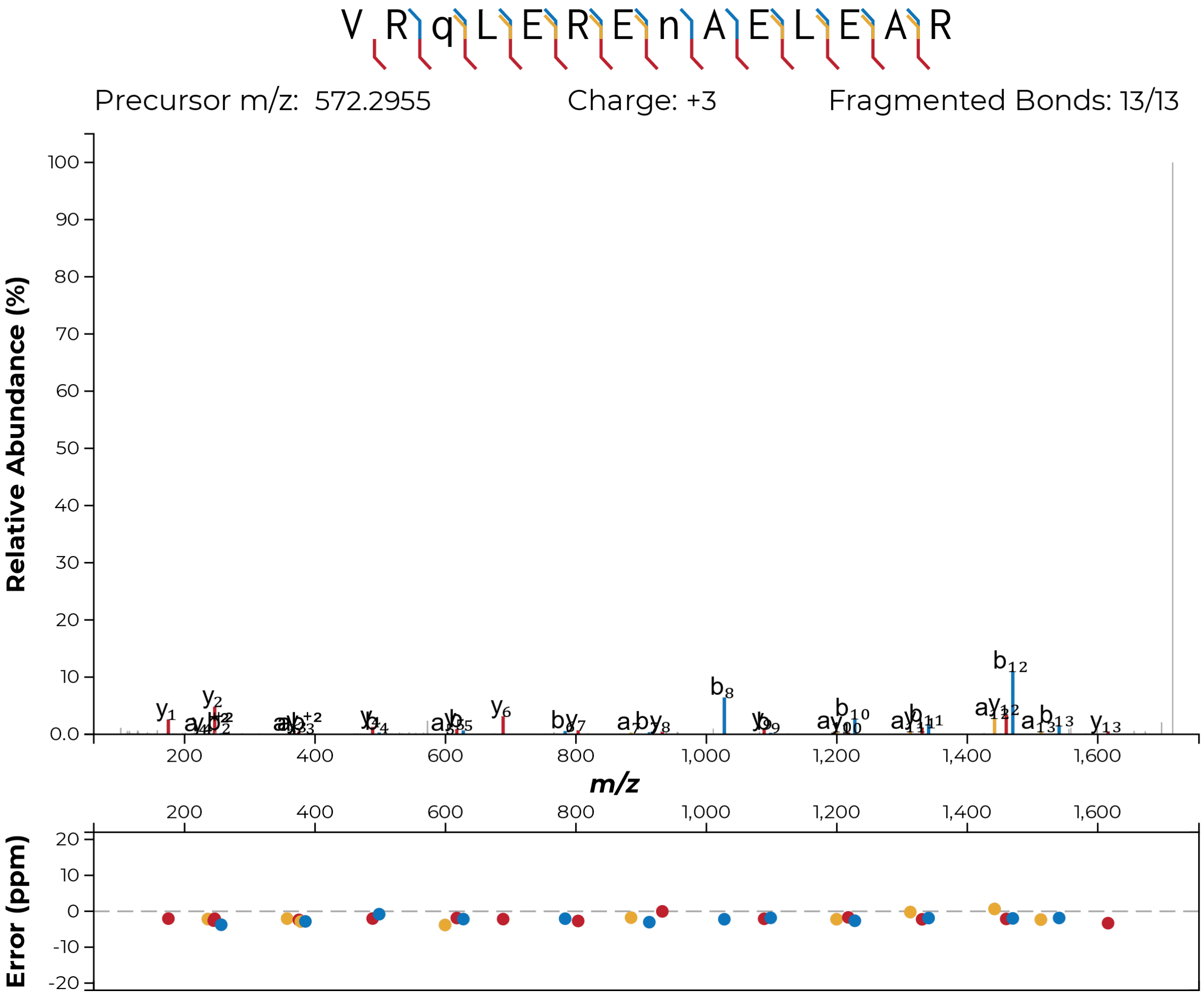

g A0A5E4CA63 VRQLERENAELEAR scan 10340 score 218; Marmotini, Cricetinae, Ornithorhynchus anatinus

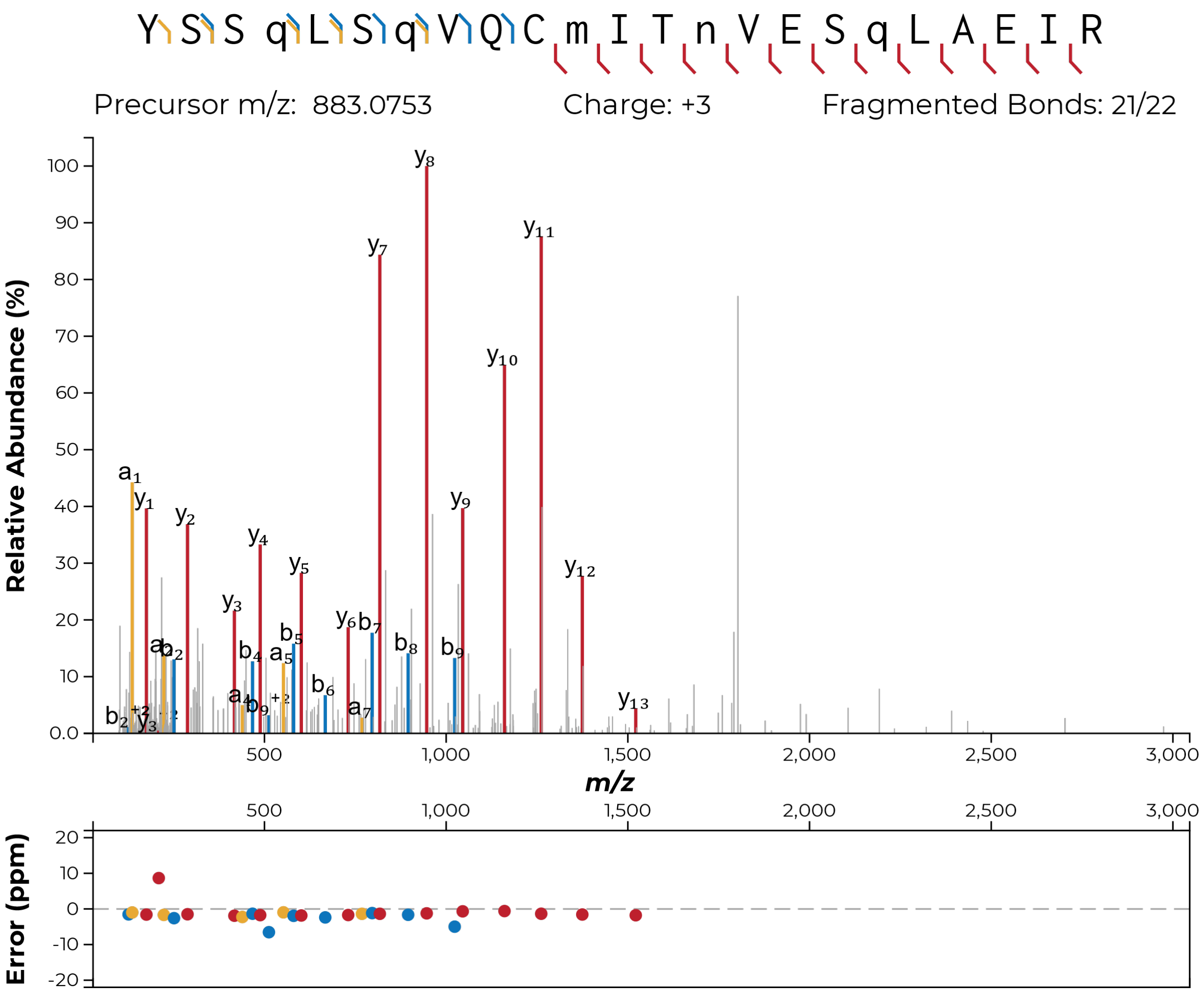

h A0A5E4CA63 YSSQLSQVQCMITNVESQLAEIR scan 25068 score 156; Eutheria (only rodents are Marmota sp. and Ictidomys tridecemlineatus; not Ornithorhynchus anatinus)

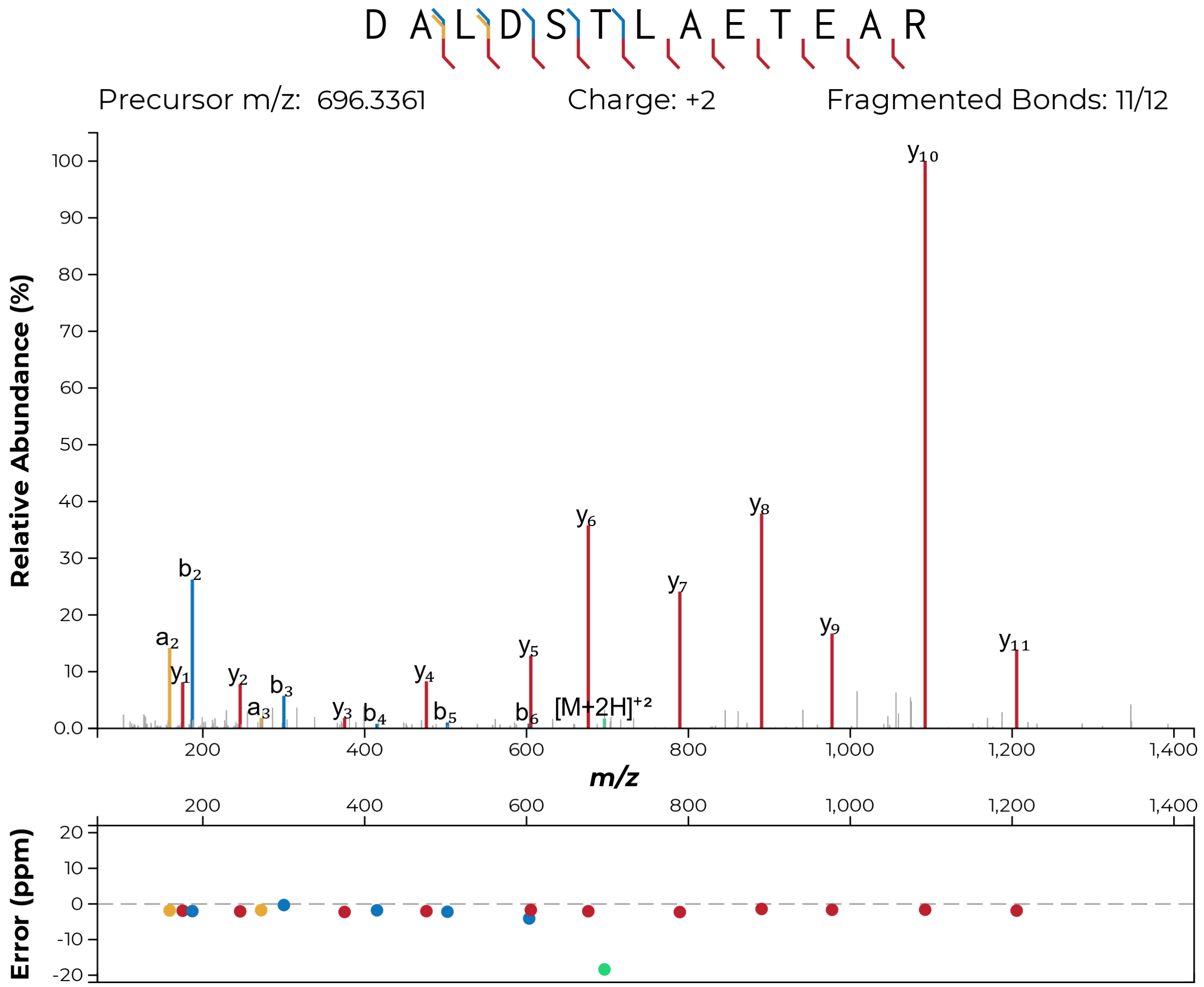

i XP_040124725 DALDSTLAETEAR scan 12583 score 167; Rodentia + Loxodonta Africana

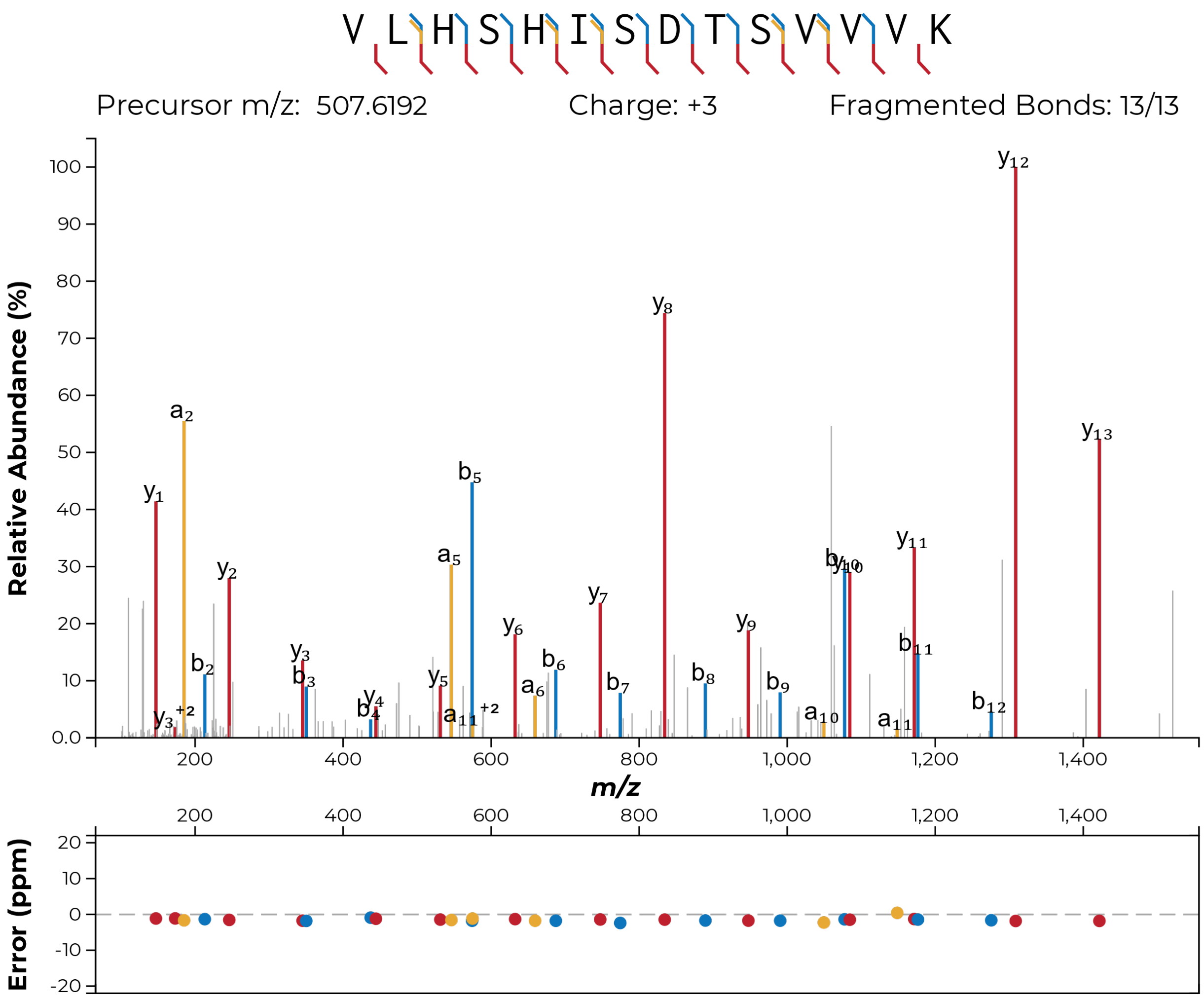

j MBZ3879004 VLHSHISDTSVVVK scan 10678 score 234; Neosciurus carolinensis, Erinaceus europaeus, Pteropus giganteus, and Chrysochloris asiatica, Trichechus manatus latirostris

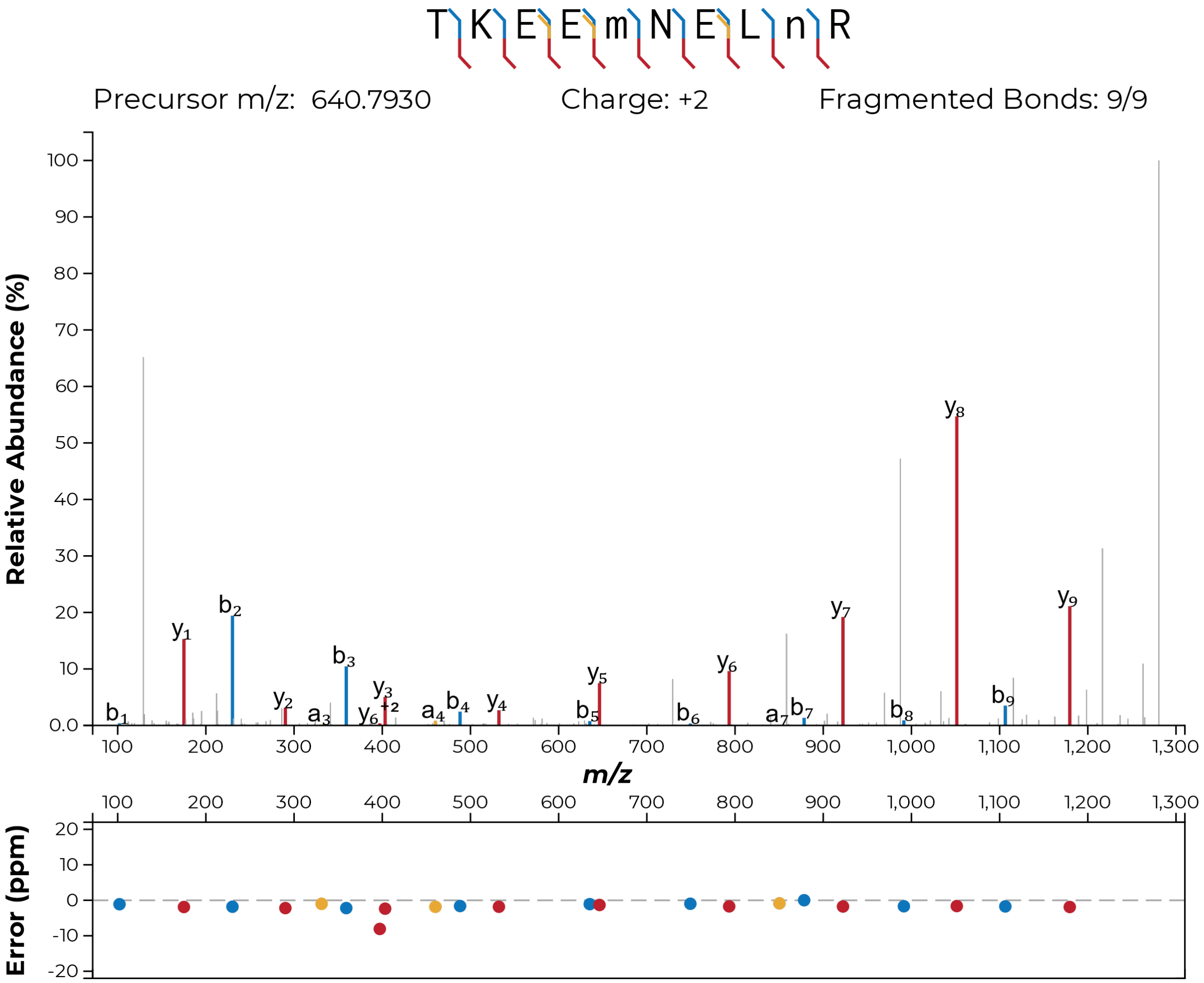

k MBZ3879004 TKEEMNELNR scan 3638 score 237; Theria (but NOT Erinaceus europaeus, Pteropus giganteus, nor Chrysochloris asiatica, Trichechus manatus latirostris)

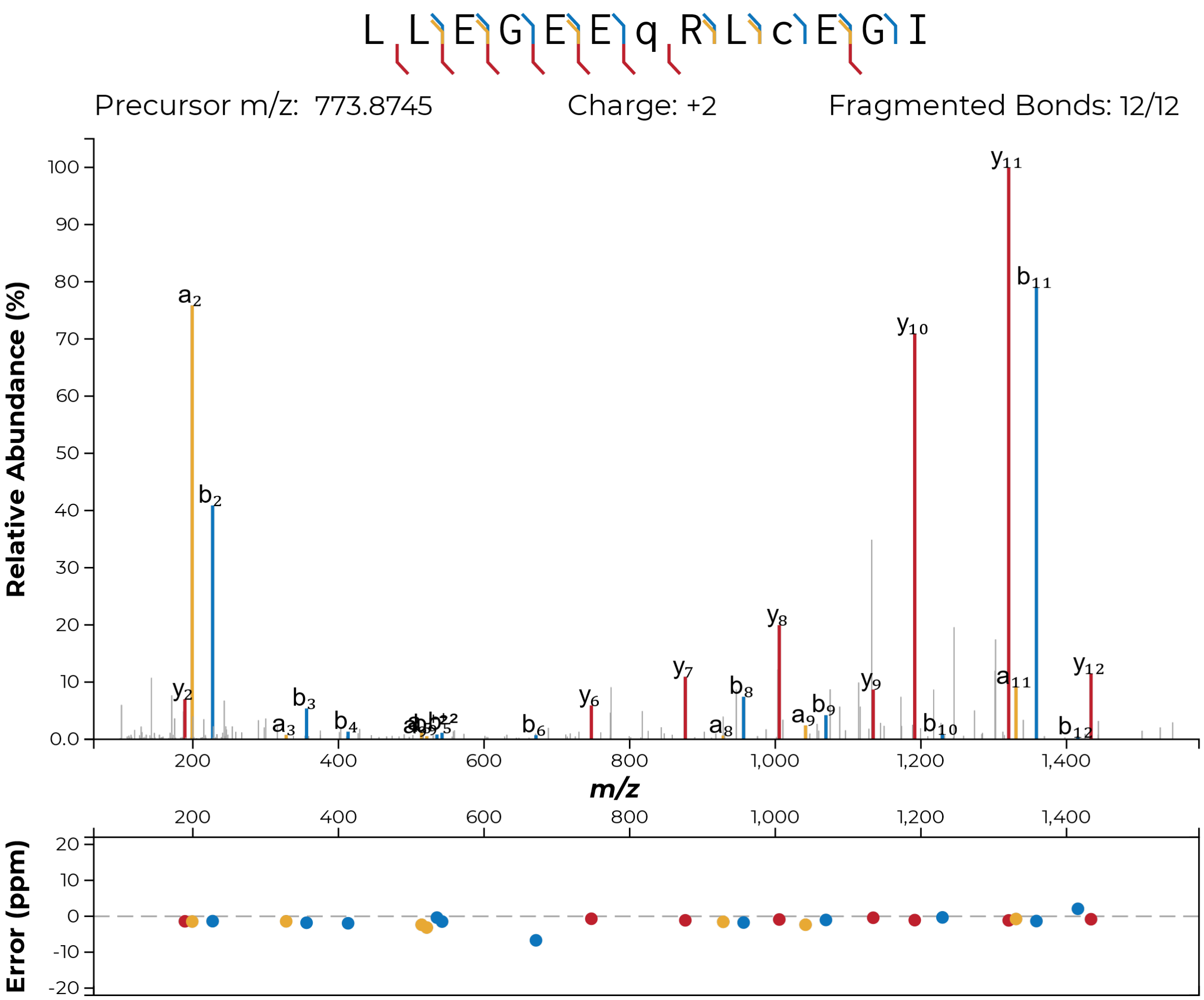

l MBZ3879004 LLEGEEQRLCEGI scan 18070 score 139; Theria (but NOT Erinaceus europaeus, Pteropus giganteus, nor Chrysochloris asiatica, Trichechus manatus latirostris)

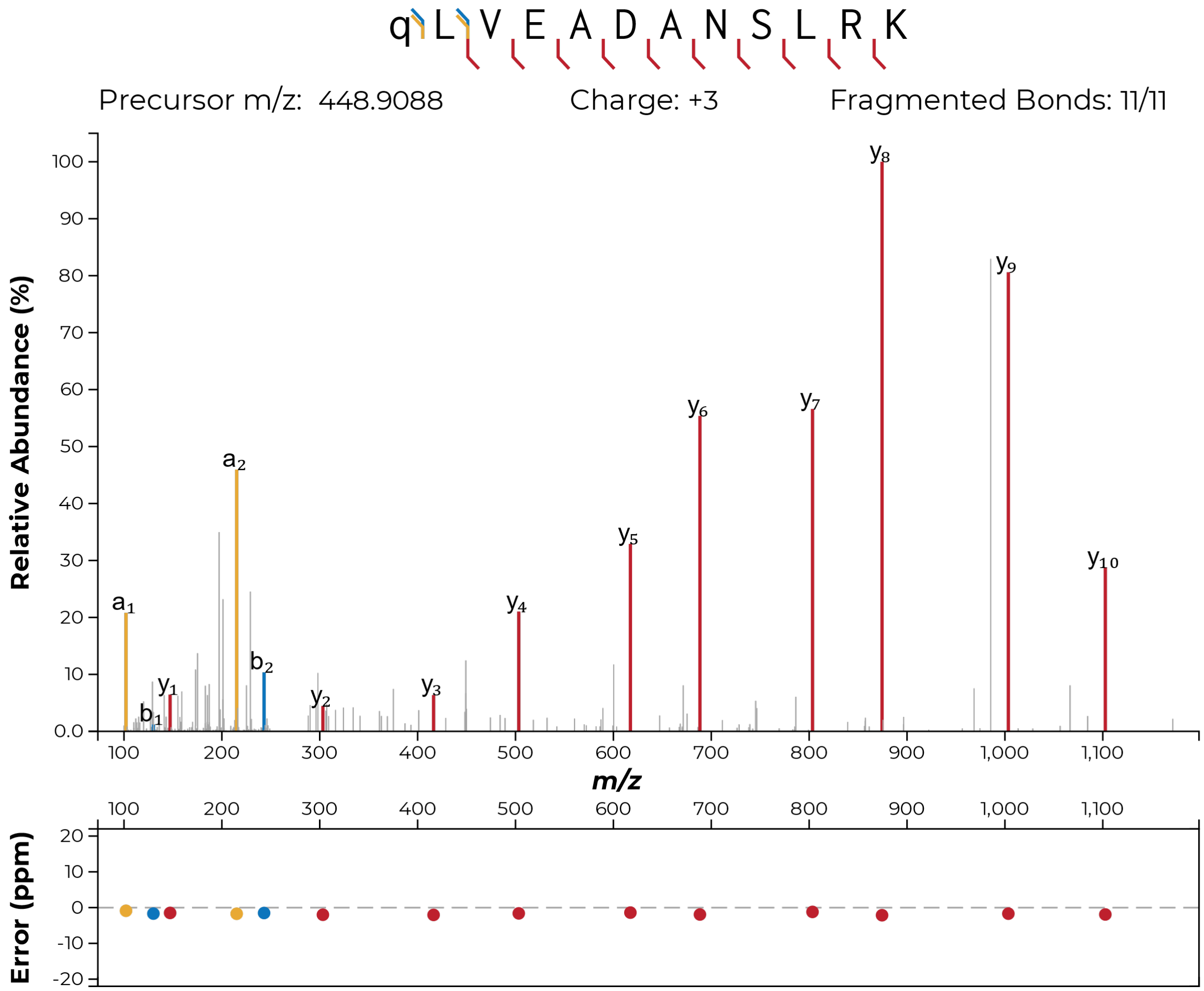

m MBZ3886840 QLVEADANSLRK scan 9499 score 110; Neosciurus carolinensis + Marmotini

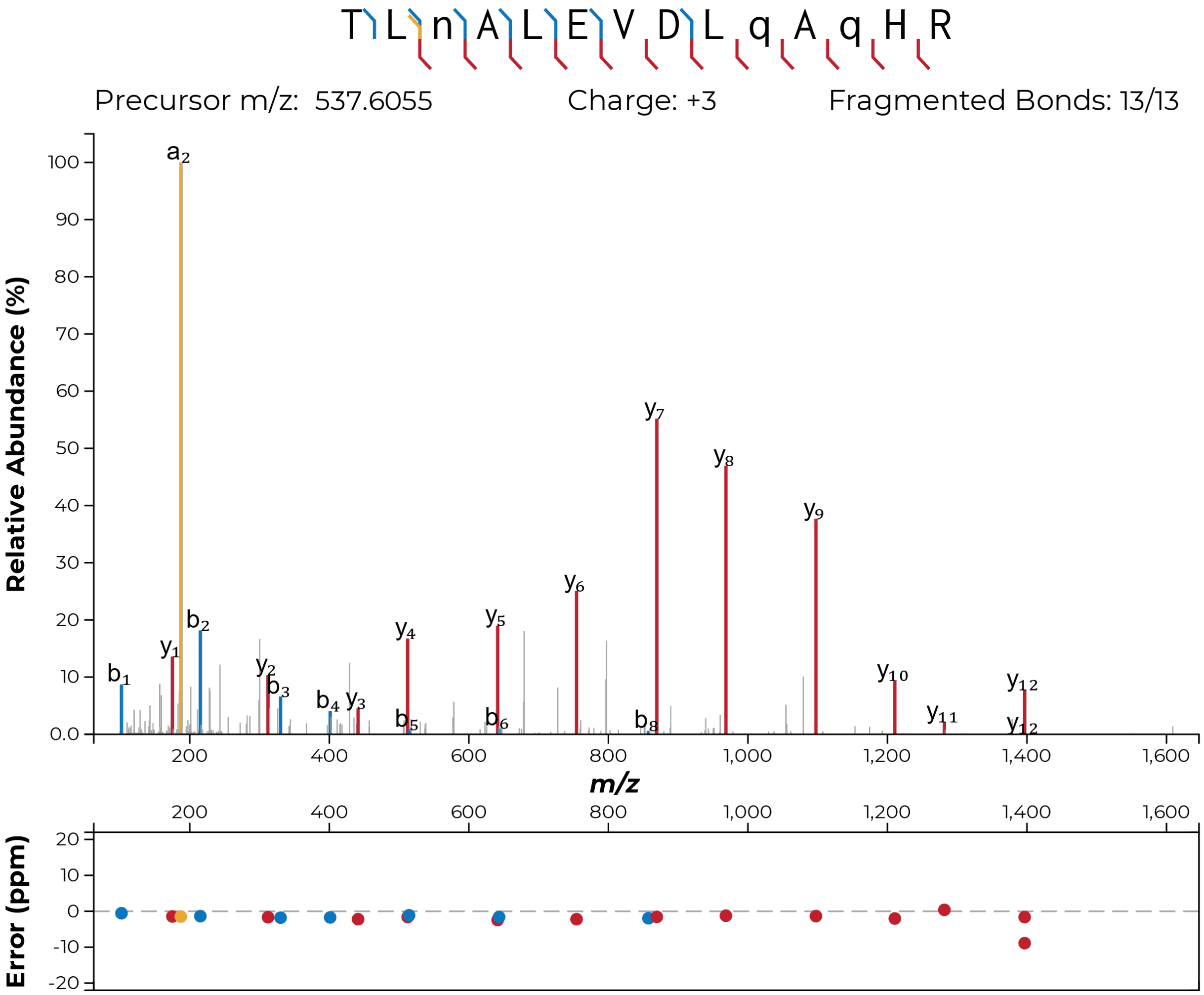

n MBZ3886840 TLNALEVDLQAQHR scan 16942 score 137; Neosciurus carolinensis

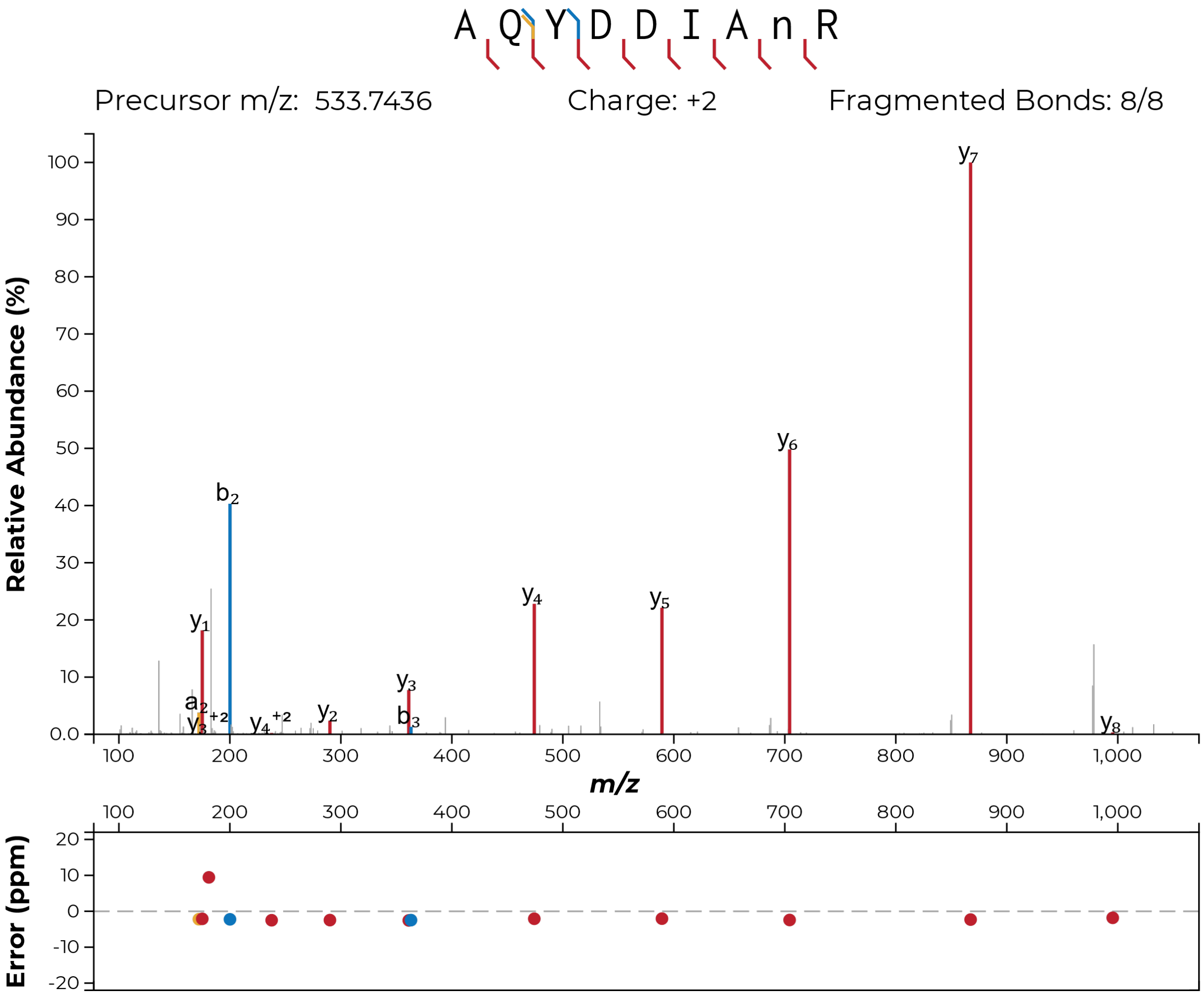

o XP_015348878 AQYDDIANR scan 7194 score 207; Marmotini, Equus sp., Erinaceus europaeus, Odobenus rosmarus divergens, and Trichechus manatus latirostris

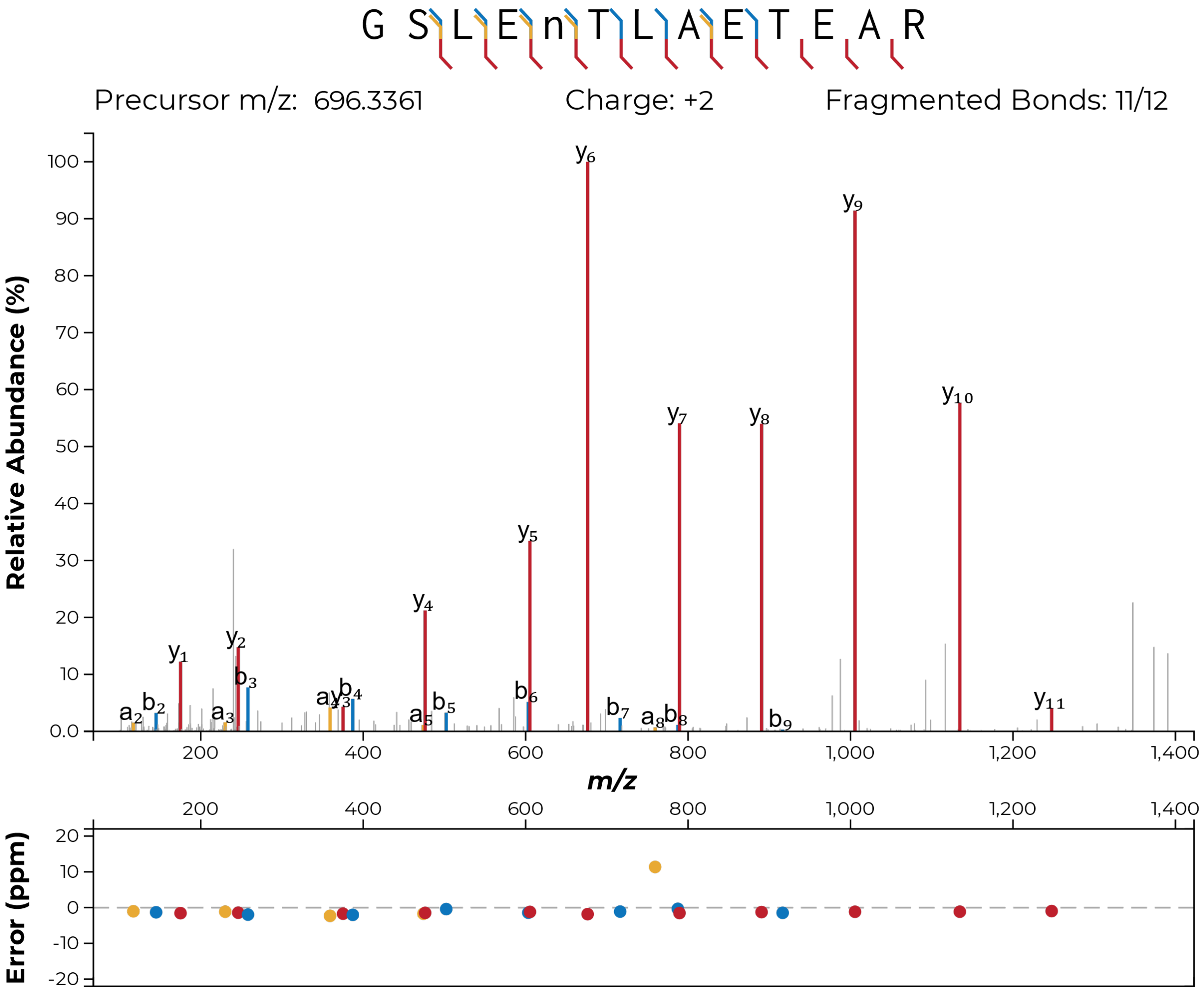

p XP_005321900 GSLENTLAETEAR scan 14842 score 248; Marmotini, Lemur catta, and Microcebus murinus

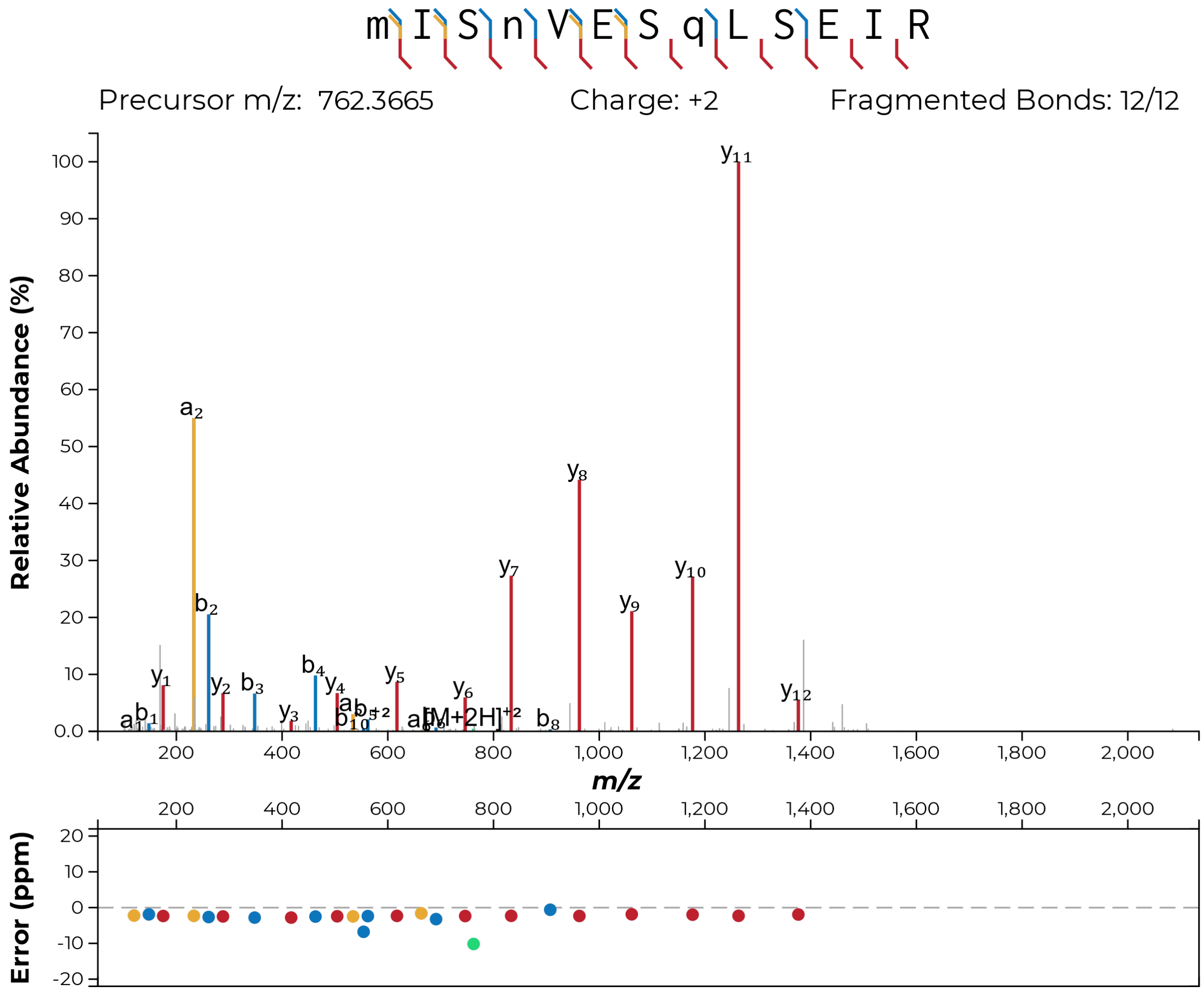

q red squirrel suspected peptide MISNVESQLSEIR scan 18321 score 199

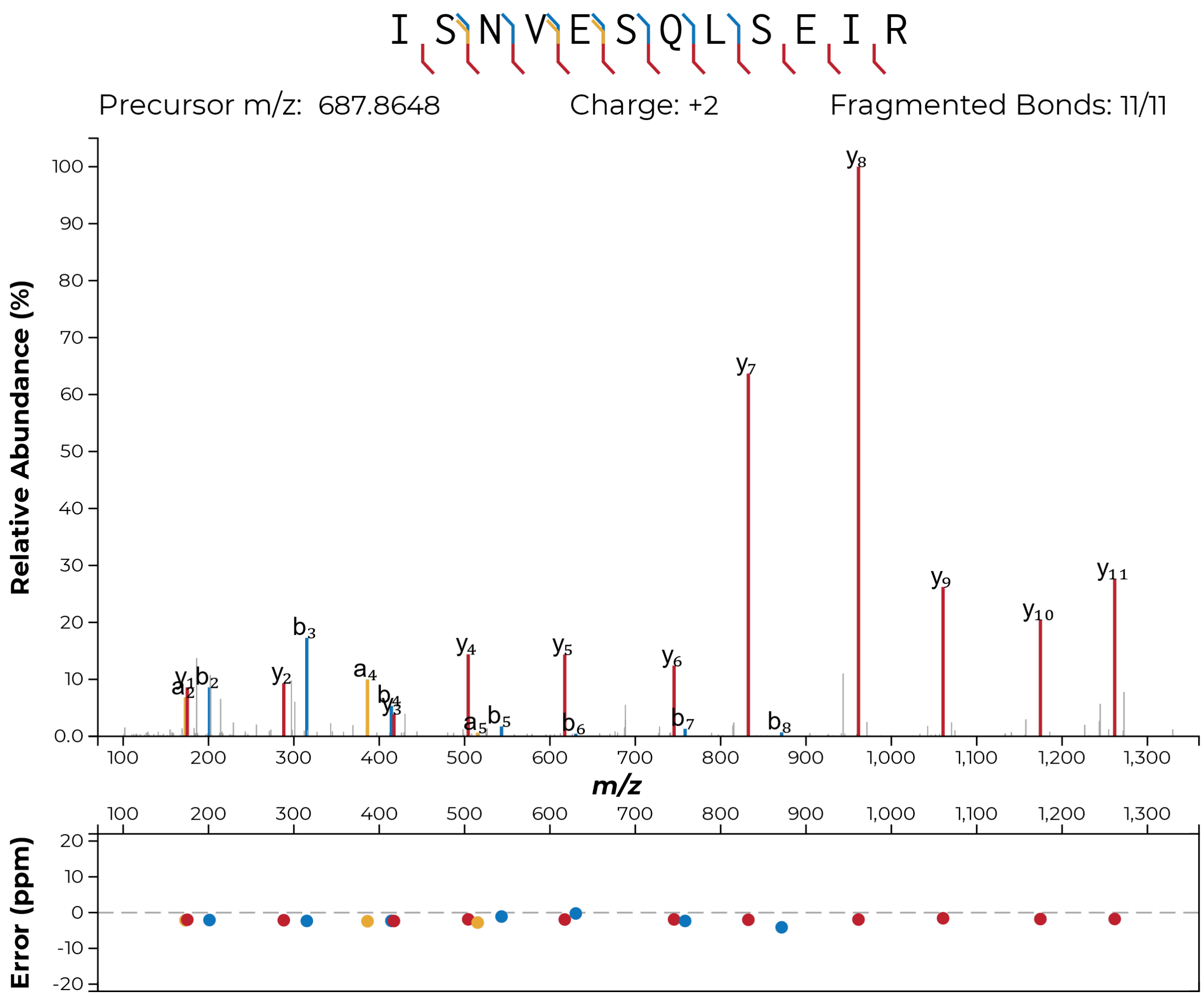

r red squirrel suspected peptide ISNVESQLSEIR scan 14881 score 286

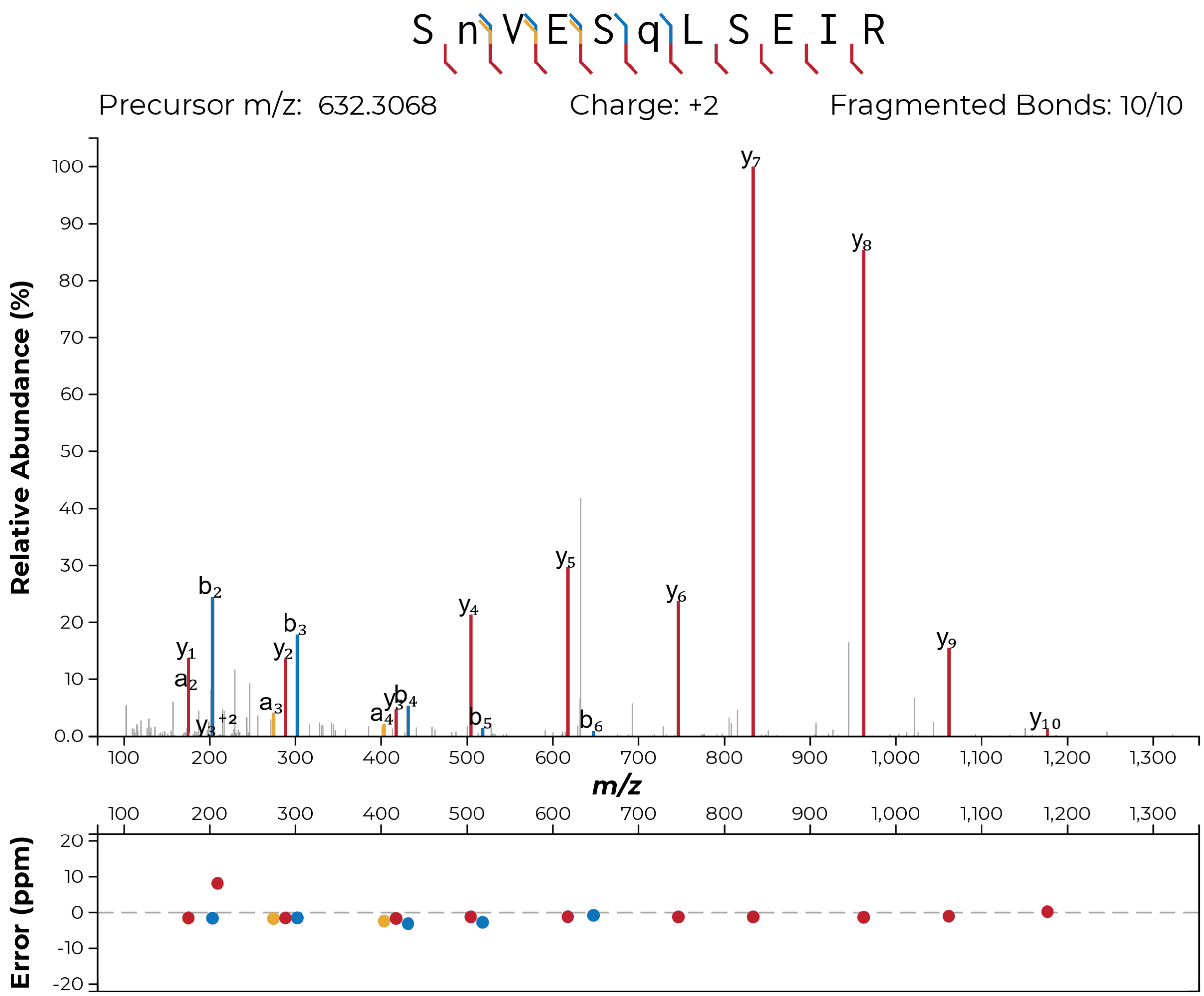

s red squirrel suspected peptide SNVESQLSEIR scan 15439 score 169

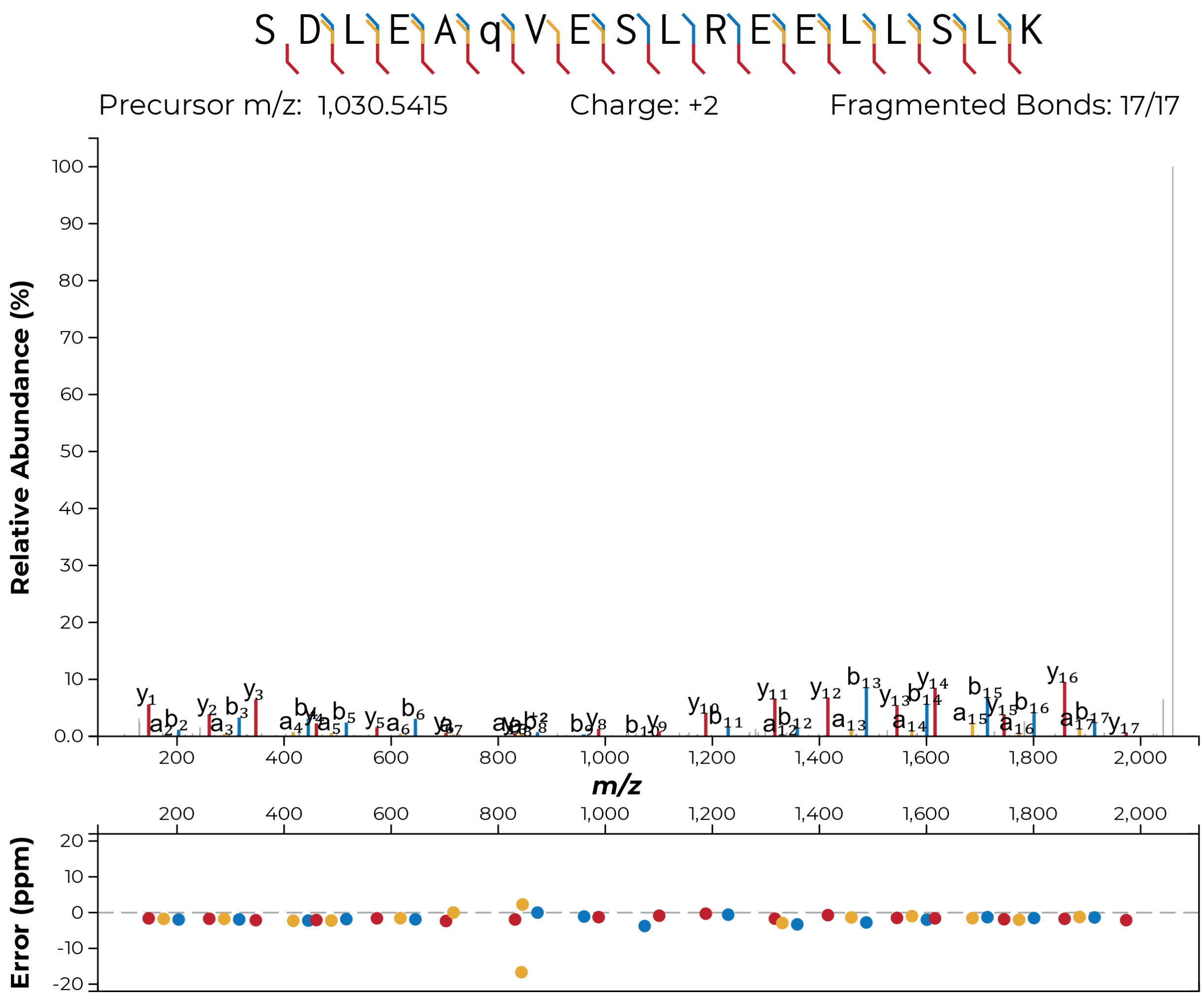

t red squirrel suspected peptide SDLEAQVESLREELLSLK scan 24673 score 401

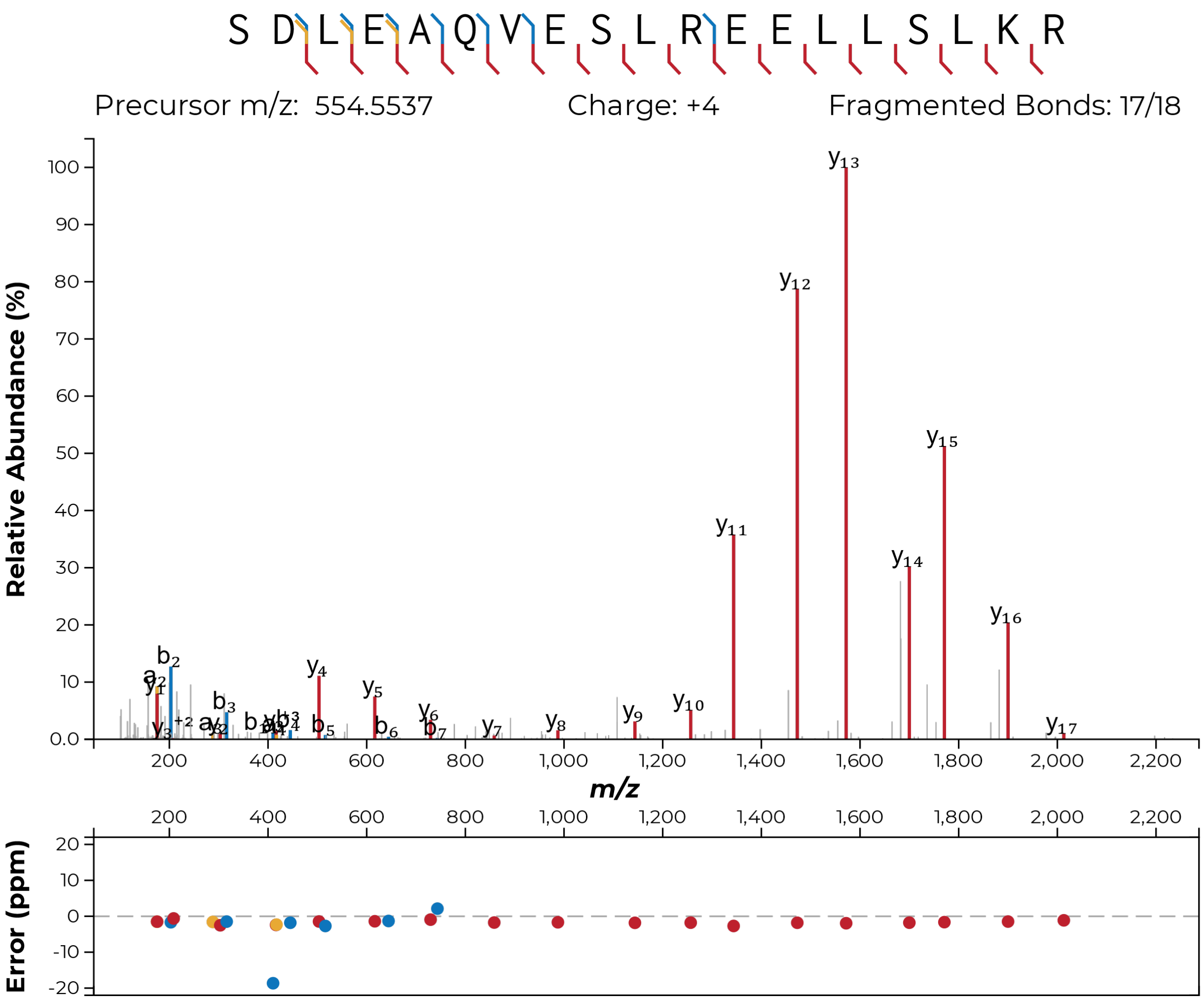

u red squirrel suspected peptide SDLEAQVESLREELLSLKR scan 23259 score 165

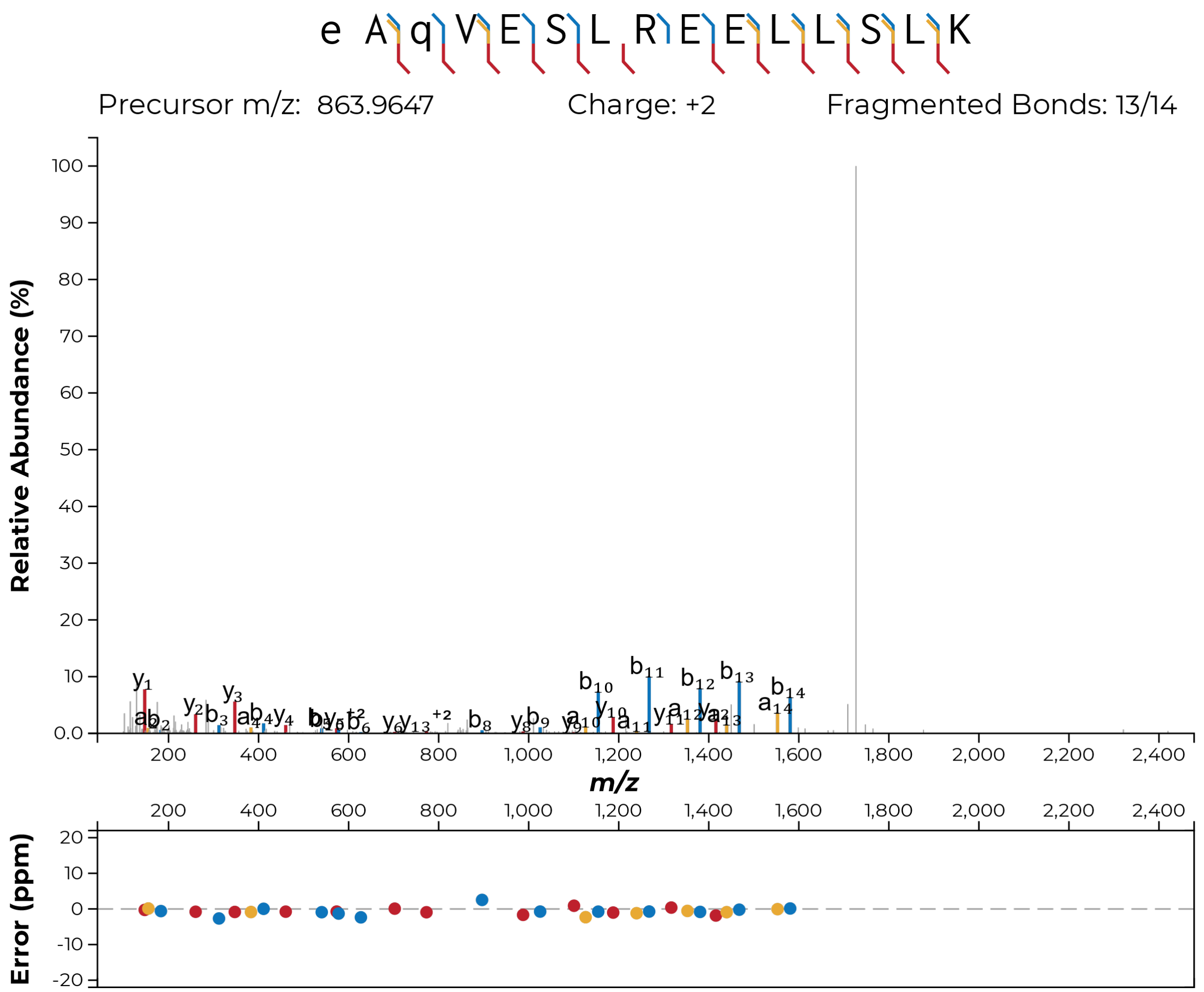

v red squirrel suspected peptide EAQVESLREELLSLK scan 23344 score 109

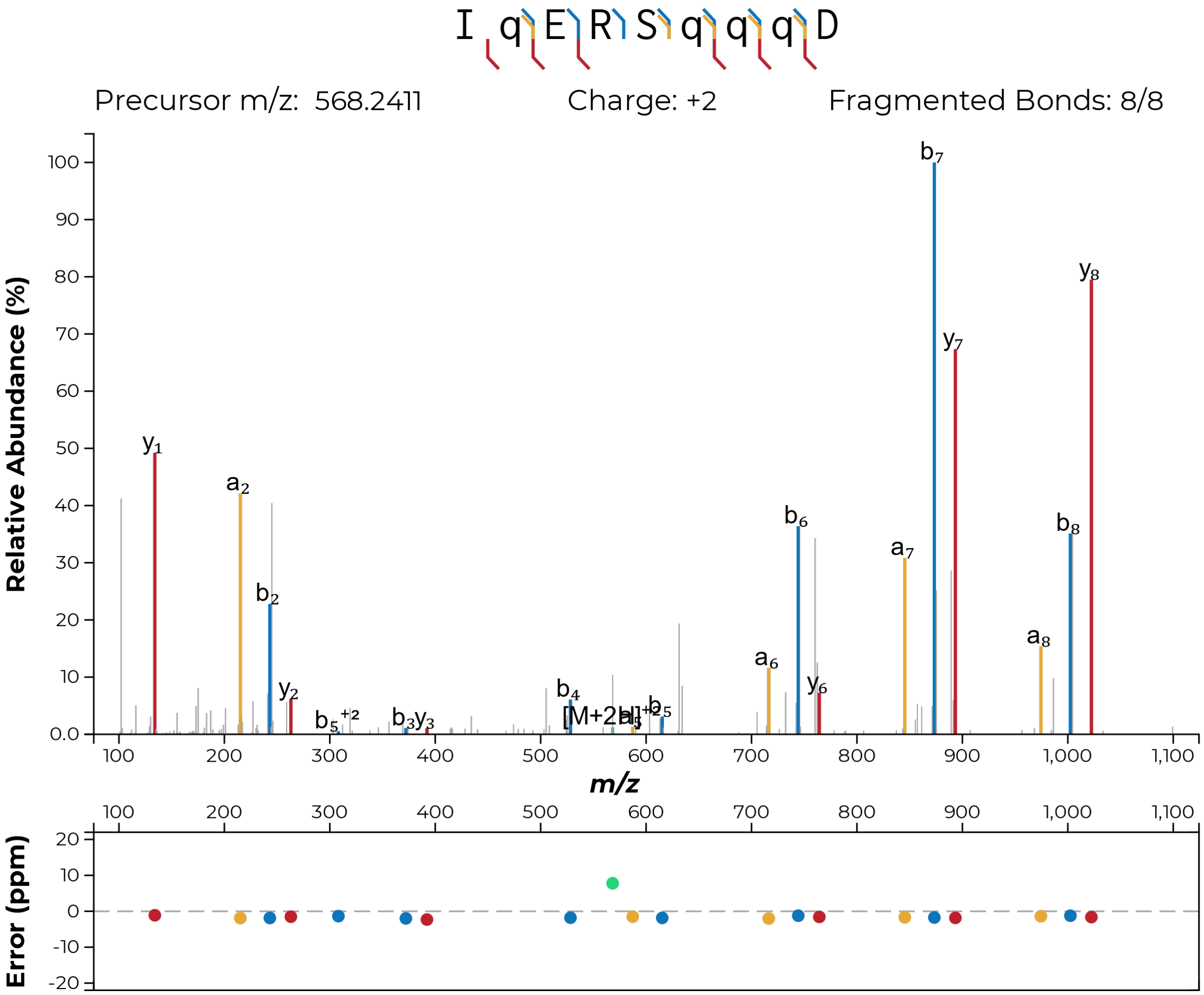

w red squirrel suspected peptide IQERSQQQD scan 2987 score 127

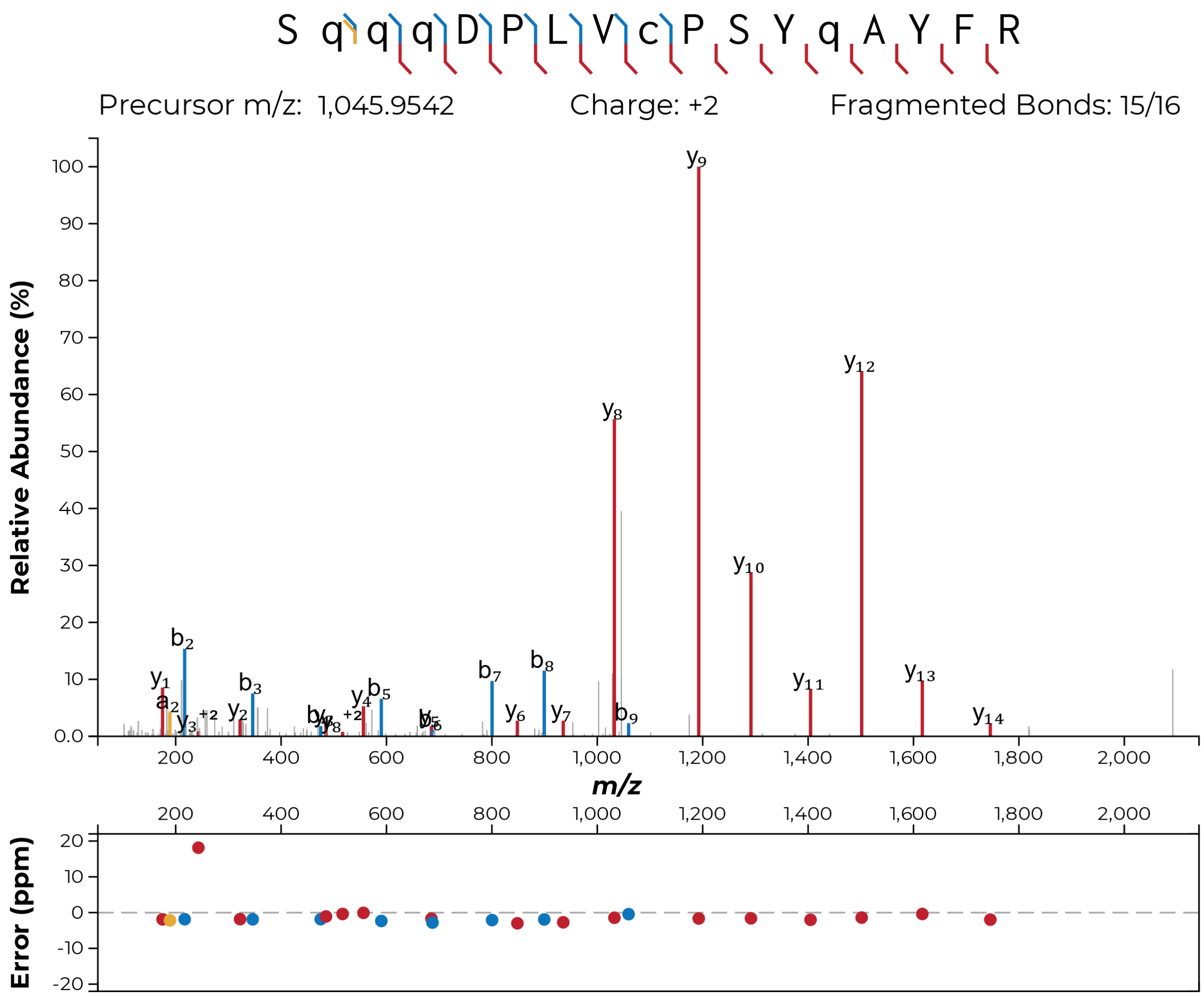

x red squirrel suspected peptide SQQQDPLVCPSYQAYFR scan 21008 score 161

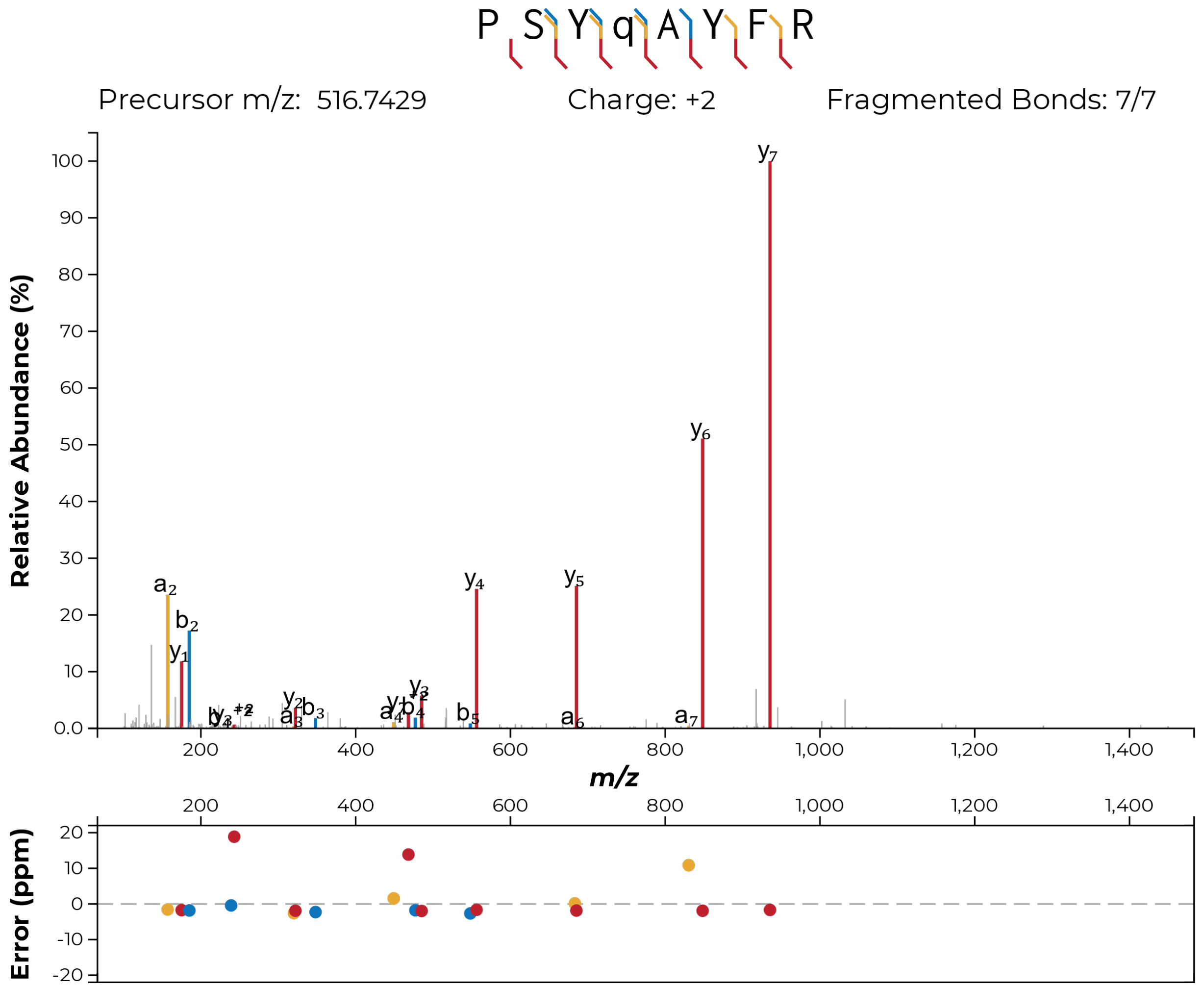

y red squirrel suspected peptide PSYQAYFR scan 14097 score 168

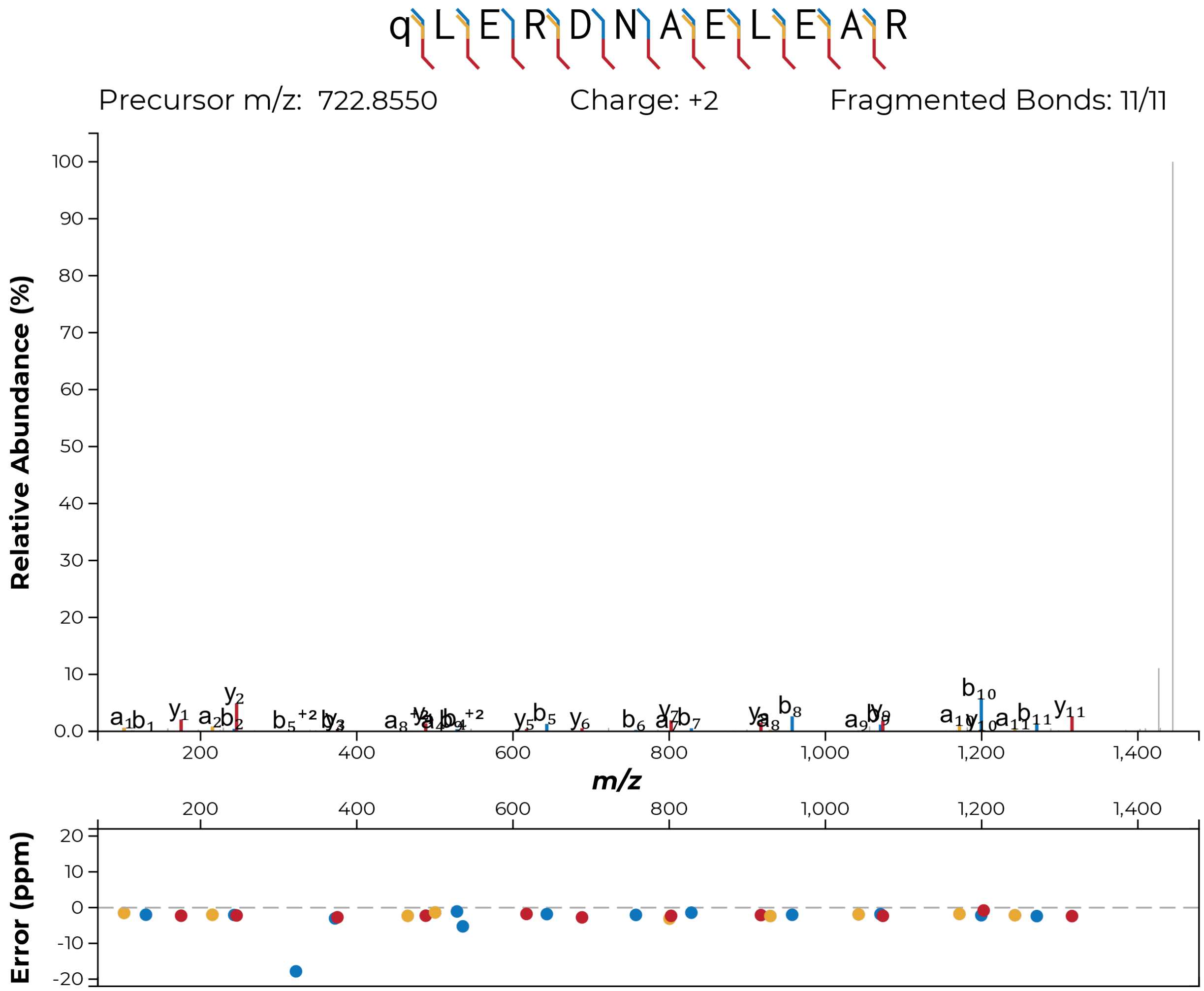

z red squirrel suspected peptide QLERDNAELEAR scan 7486 score 381

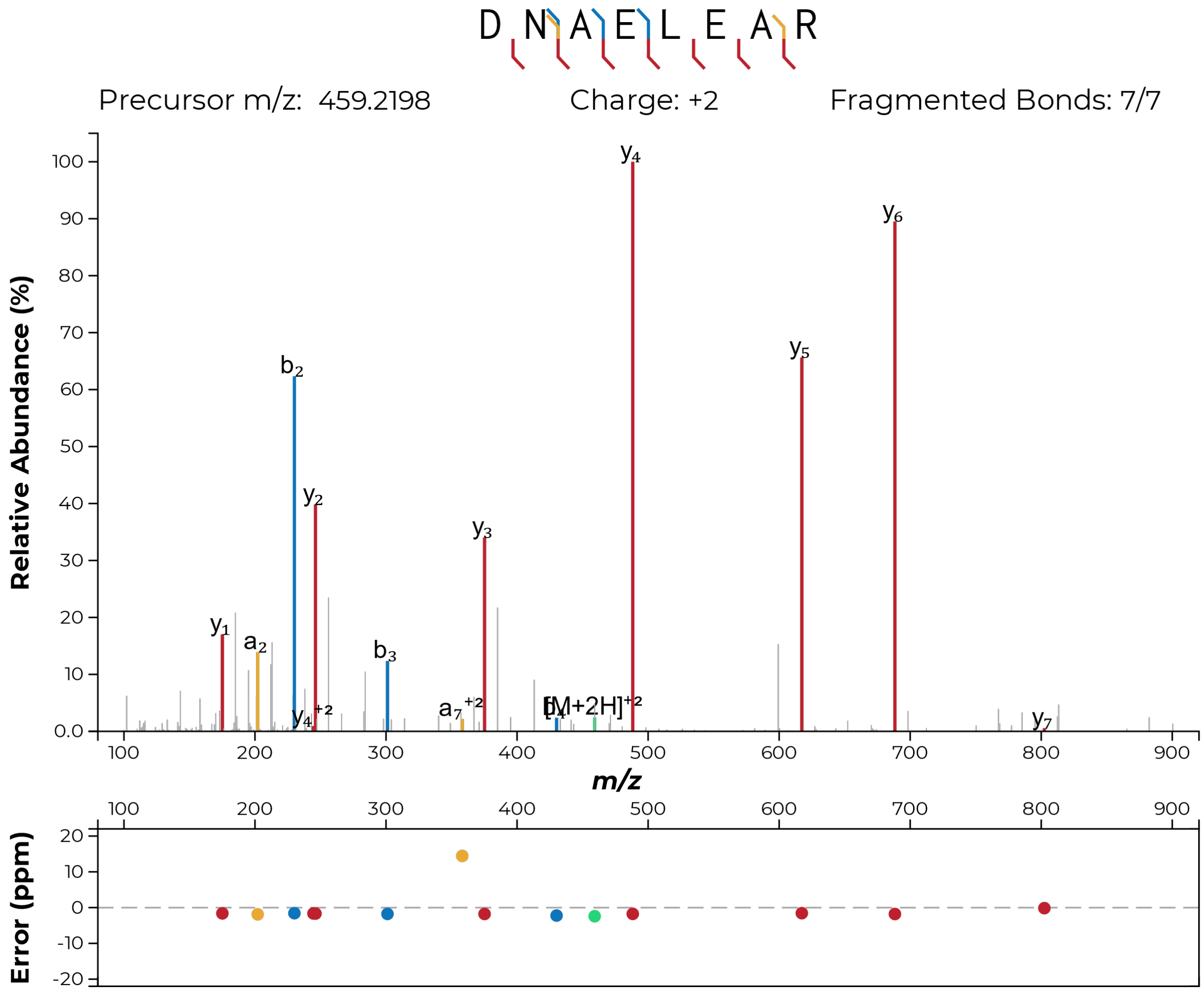

aa red squirrel suspected peptide DNAELEAR scan 5189 score 208

B. Sample F11 (bb-tt)

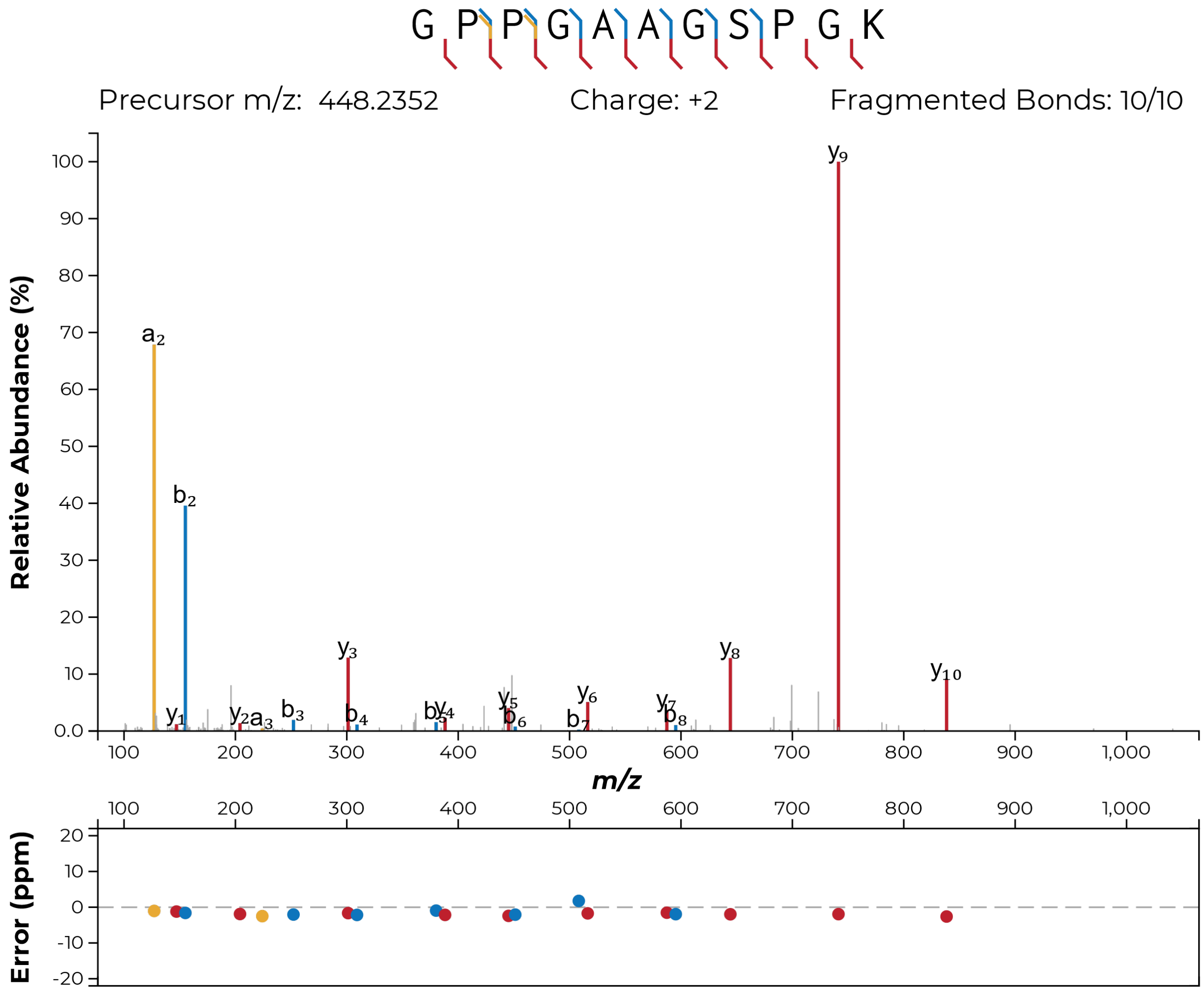

ø XP_029784577.1 GPPGAAGSPGK scan 3411 score 131; Feliformia

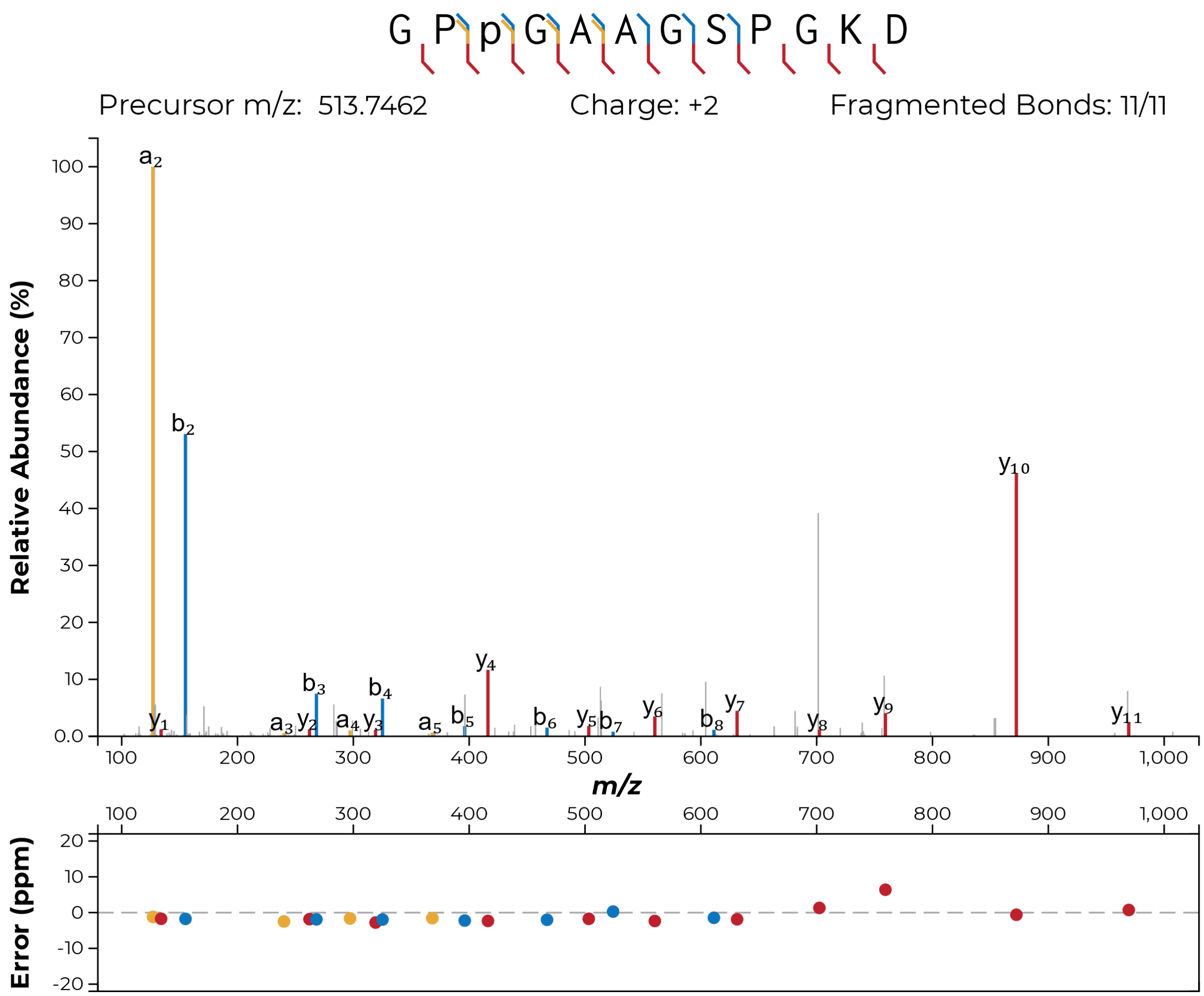

cc XP_040328585.1 GPPGAAGSPGKD scan 2826 score 117; Feliformia

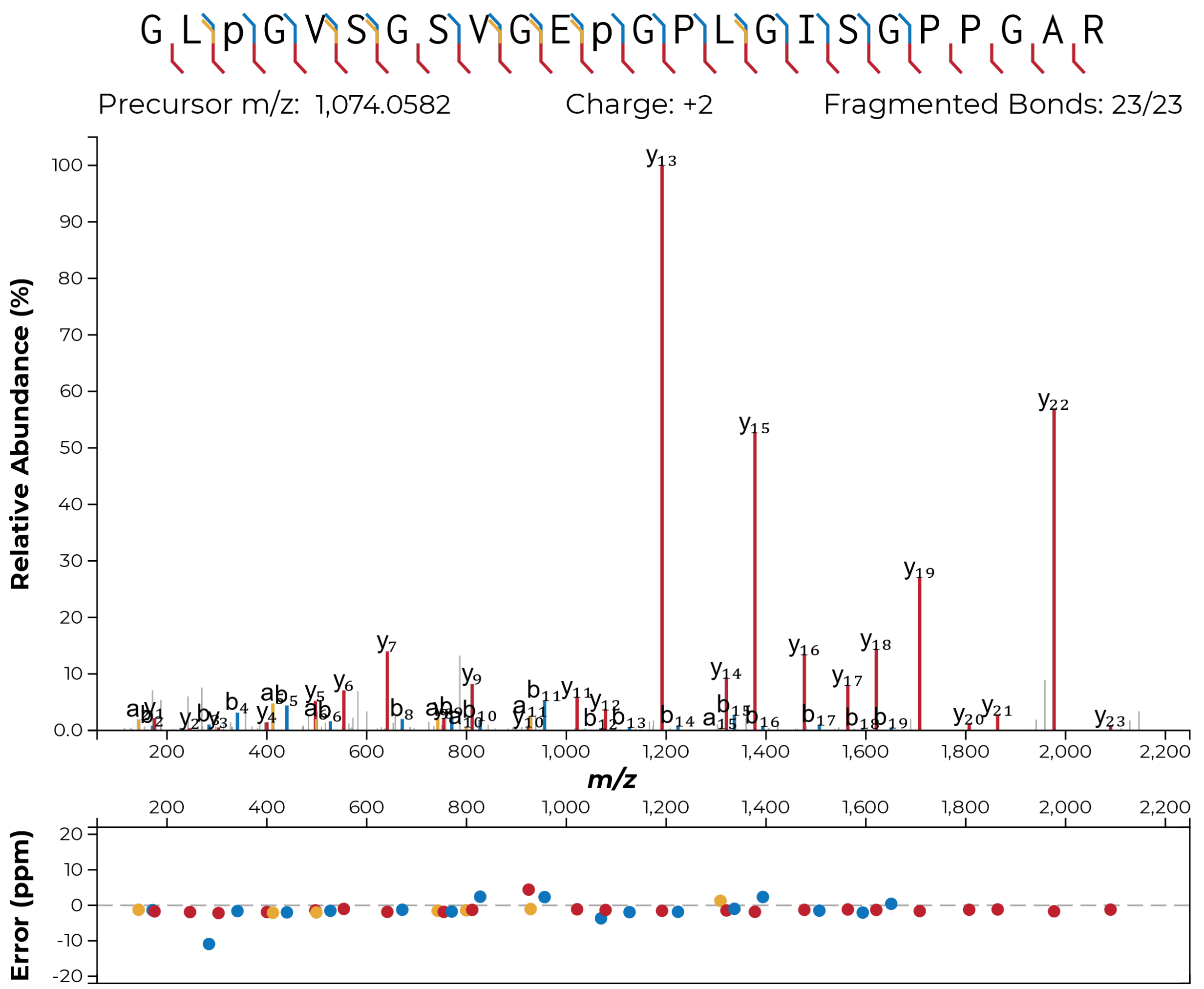

dd XP_040328585.1 GLPGVSGSVGEPGPLGISGPPGAR scan 18558 score 412; Laurasiatheria ("Old World" felids: Felis catus, Lynx pardinus, Acinonyx jubatus)

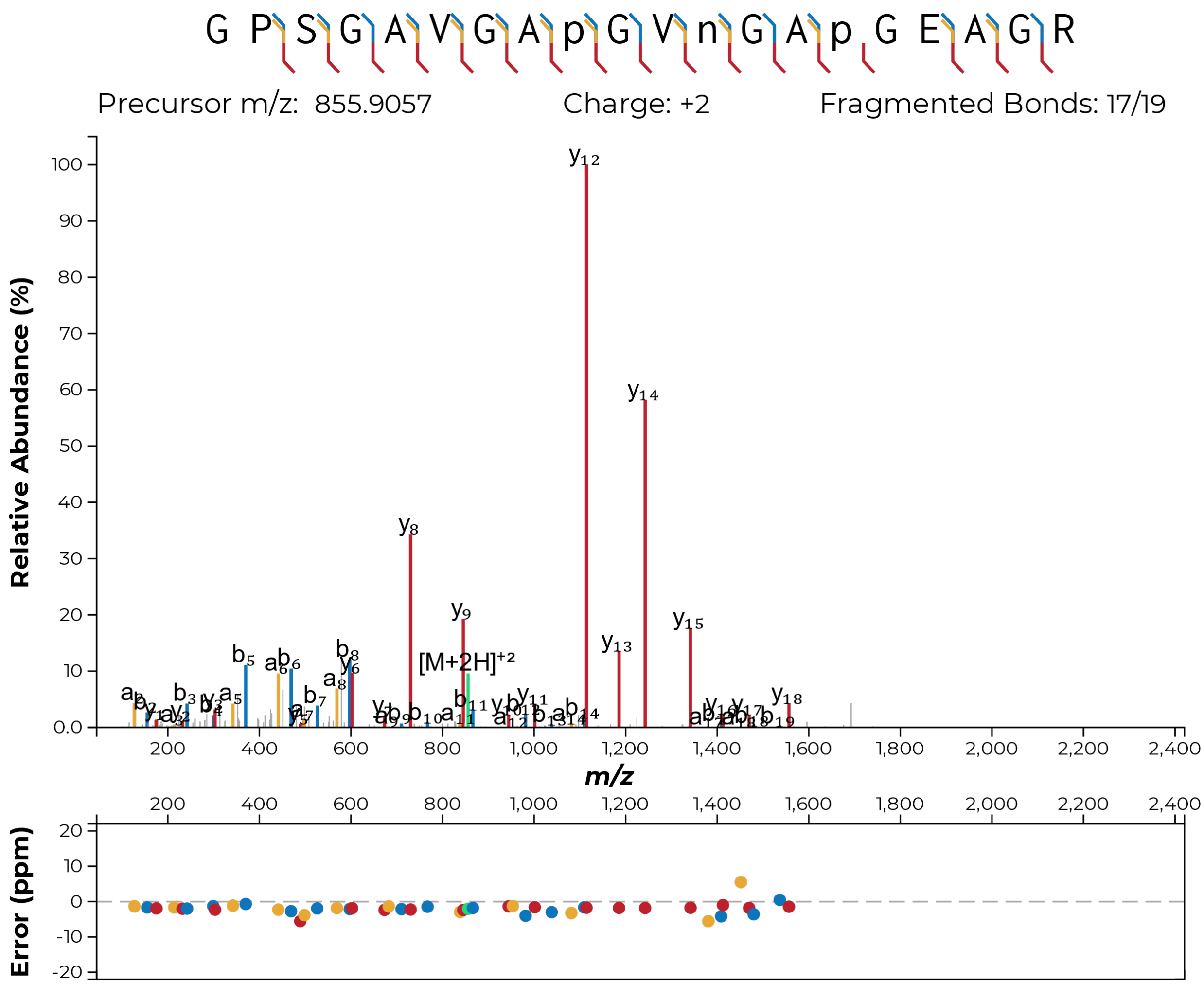

ee XP_040328585.1 GPSGAVGAPGVNGAPGEAGR scan 8121 score 231; Felidae ("Old World": Felis catus, Prionailurus bengalensis/viverrinus, Lynx pardinus, Acinonyx jubatus, Panthera tigris/leo/pardus)

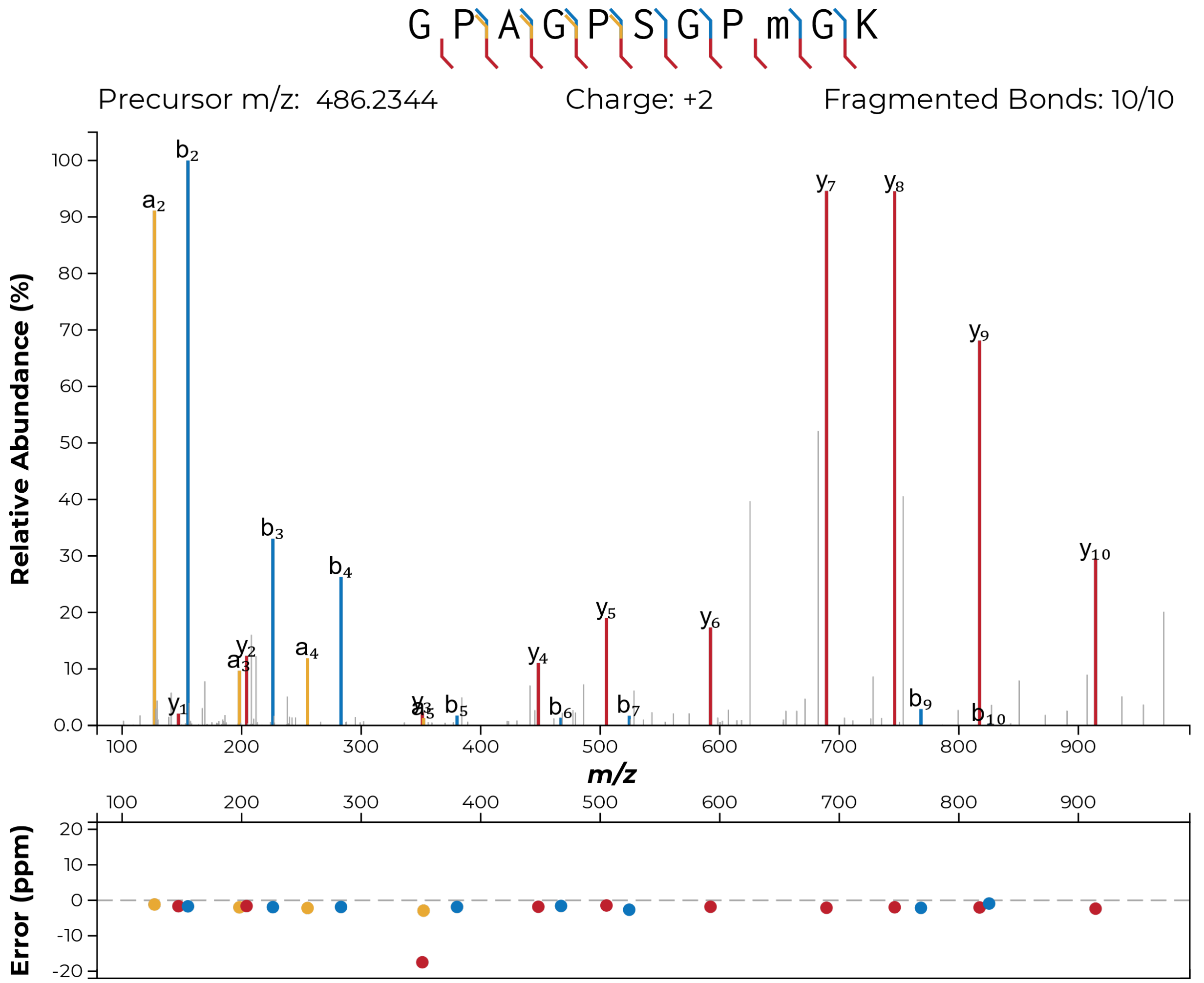

ff XP_040328585.1 GPAGPSGPMGK scan 3609 score 130; Felidae ("Old World": Felis catus, Prionailurus bengalensis/viverrinus, Lynx pardinus, Acinonyx jubatus, Panthera tigris/leo/pardus)

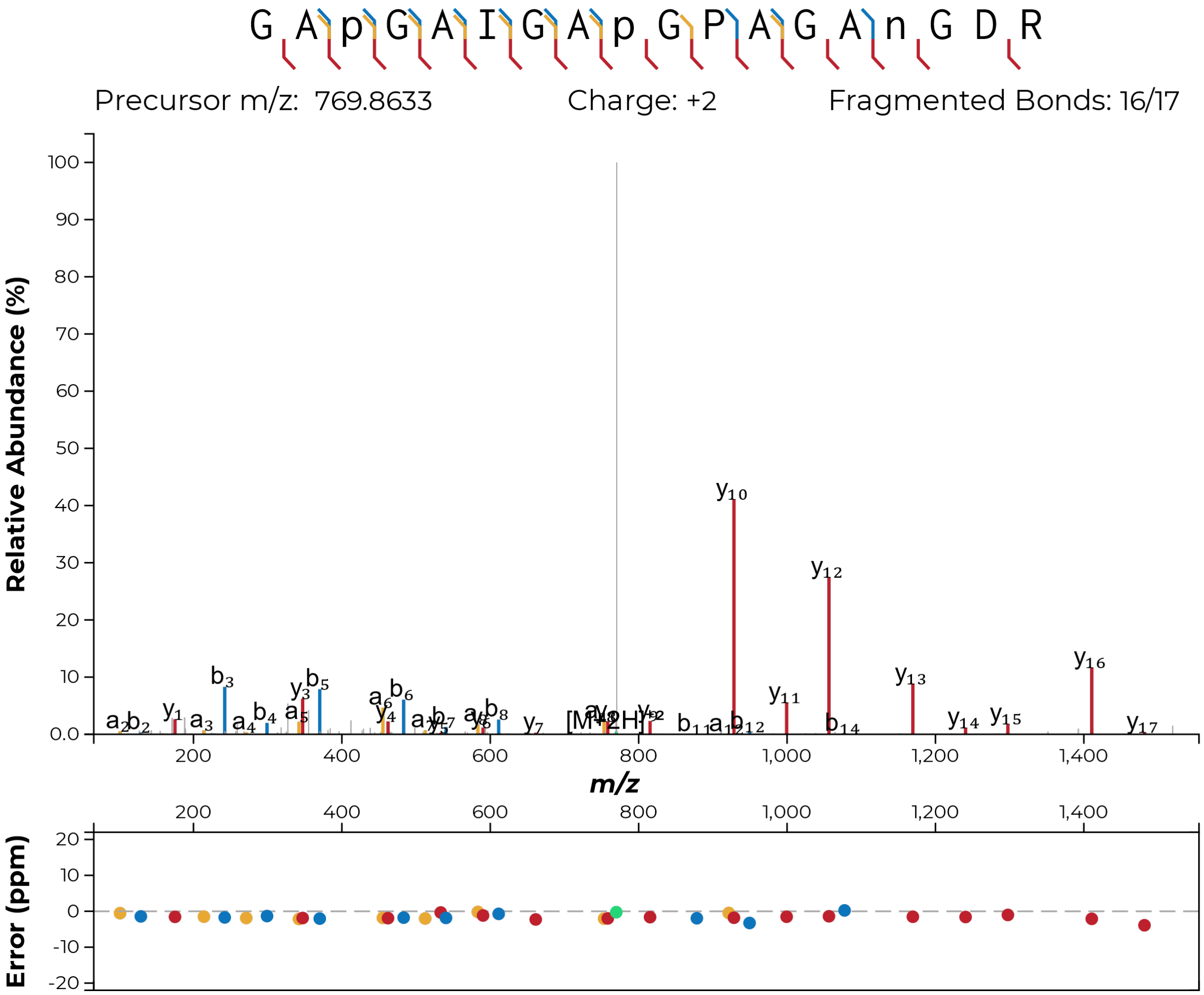

gg P02465 GAPGAIGAPGPAGANGDR scan 7672 score 190; Bos sp., Bison bison bison, Toxodon sp.

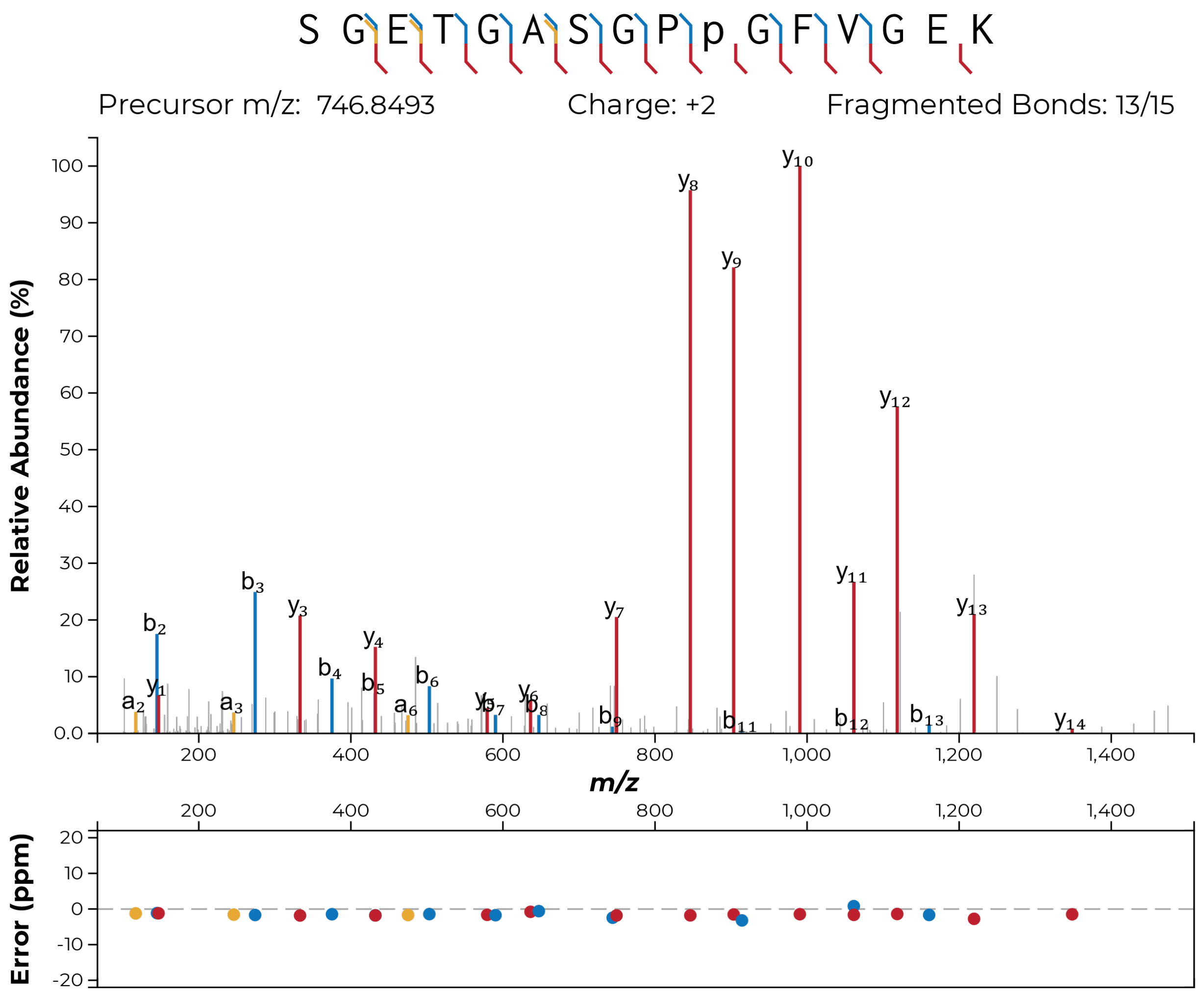

hh P02465 SGETGASGPPGFVGEK scan 9759 score 241; Bos sp., Bison bison bison, Balaenoptera acutorostrata scammoni, Mus musculus

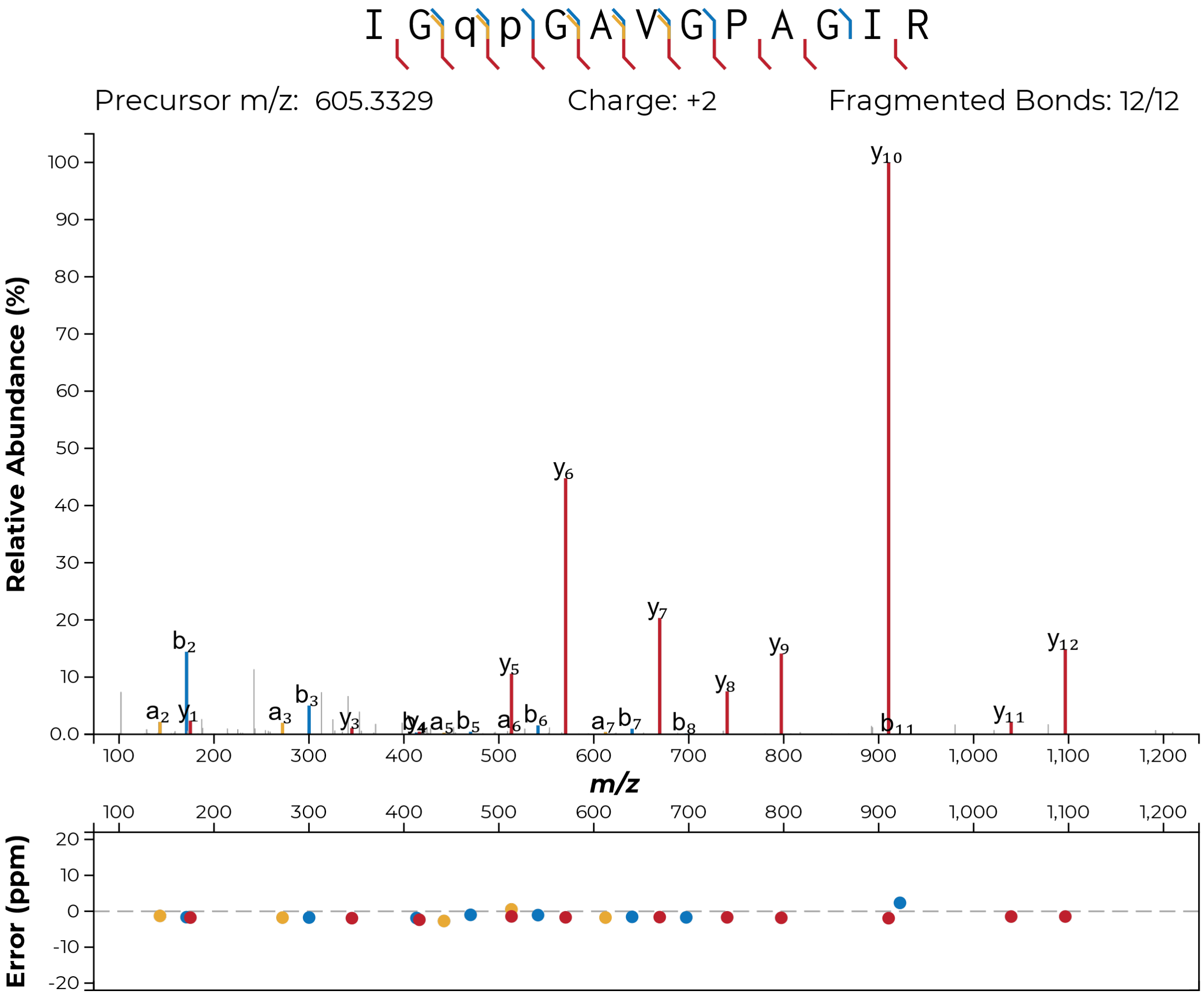

ii P02465 IGQPGAVGPAGIR scan 11233 score 192; Bovinae

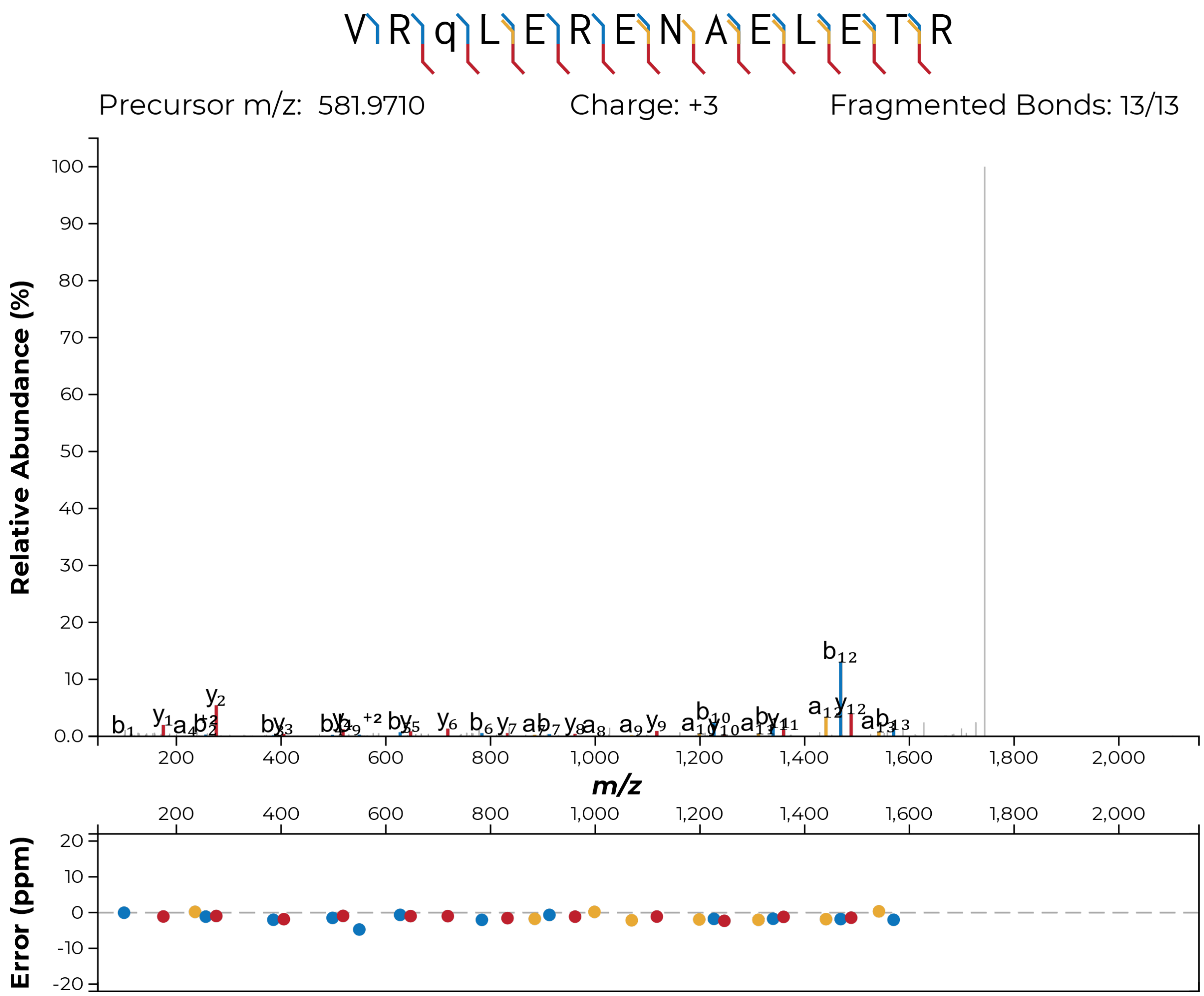

jj A0A485MV79 VRQLERENAELETR scan 8953 score 196; Felidae + Molossus molossus

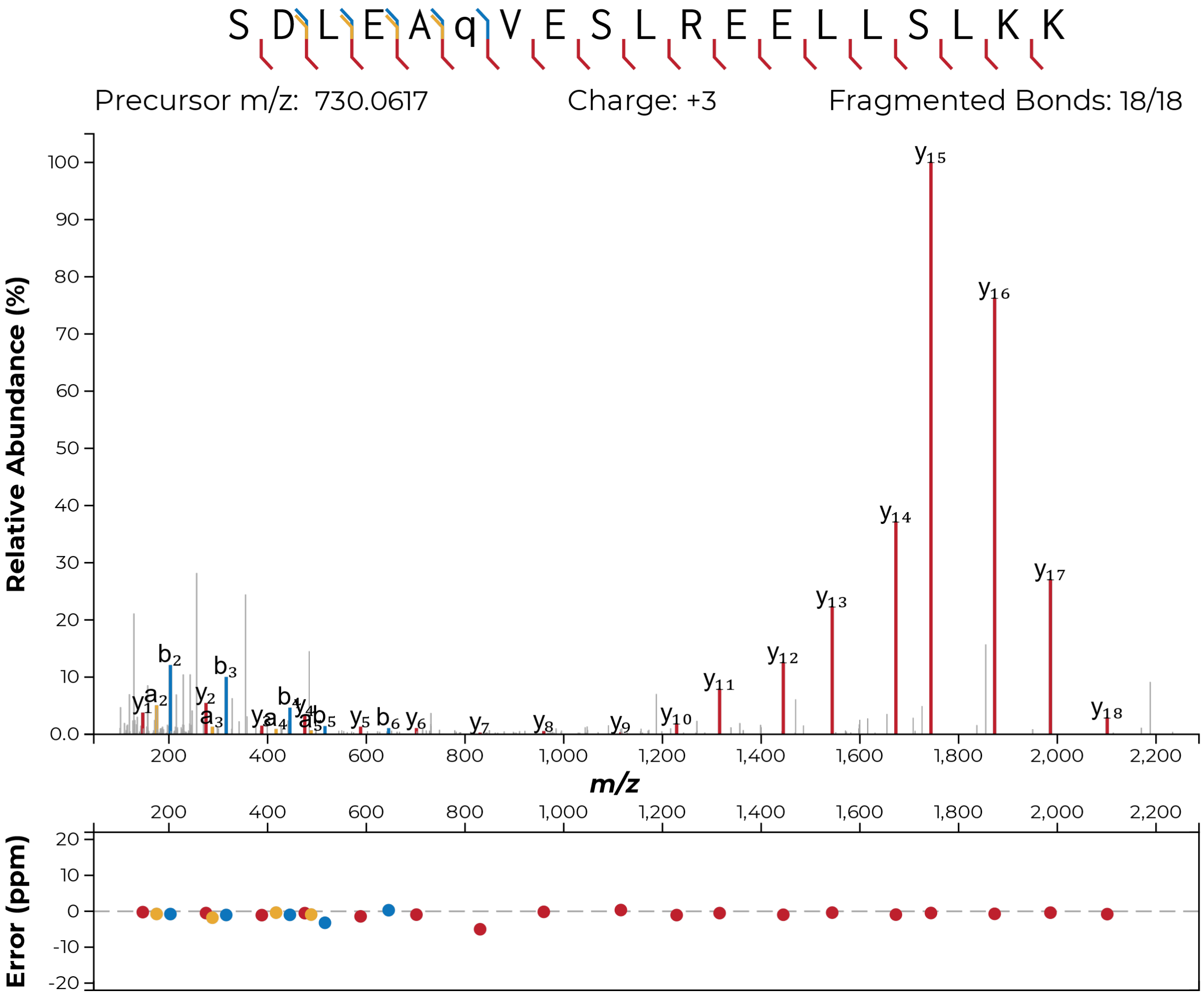

kk A0A485MV79 SDLEAQVESLREELLSLKK scan 24258 score 85; Feliformia + Chinchilla lanigera, Ochotona curzoniae, Oryctolagus cuniculus, Octodon degus

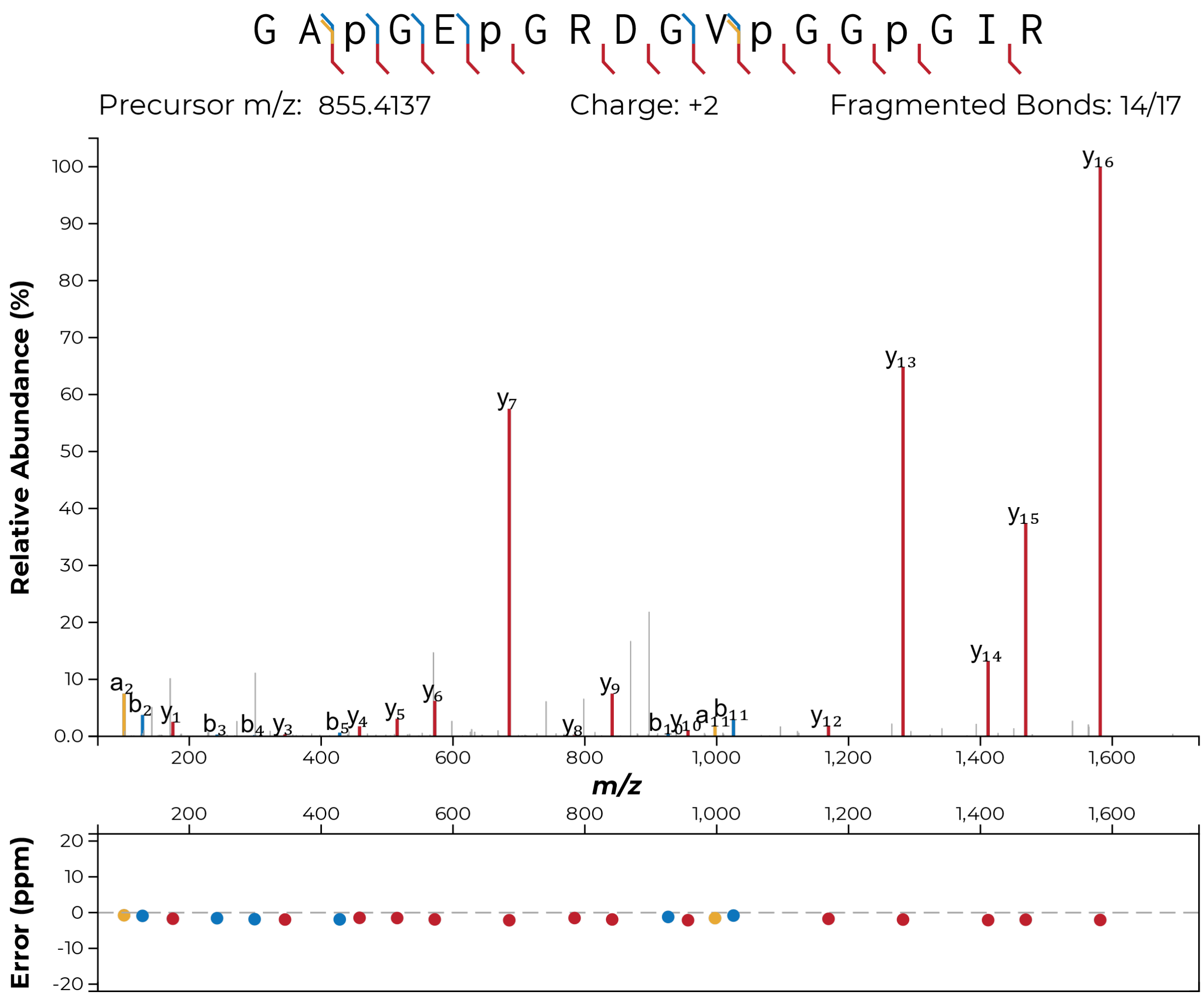

ll JACGTZ010000853_7a GAPGEPGRDGVPGGPGIR scan 6447 score 129; Feliformia

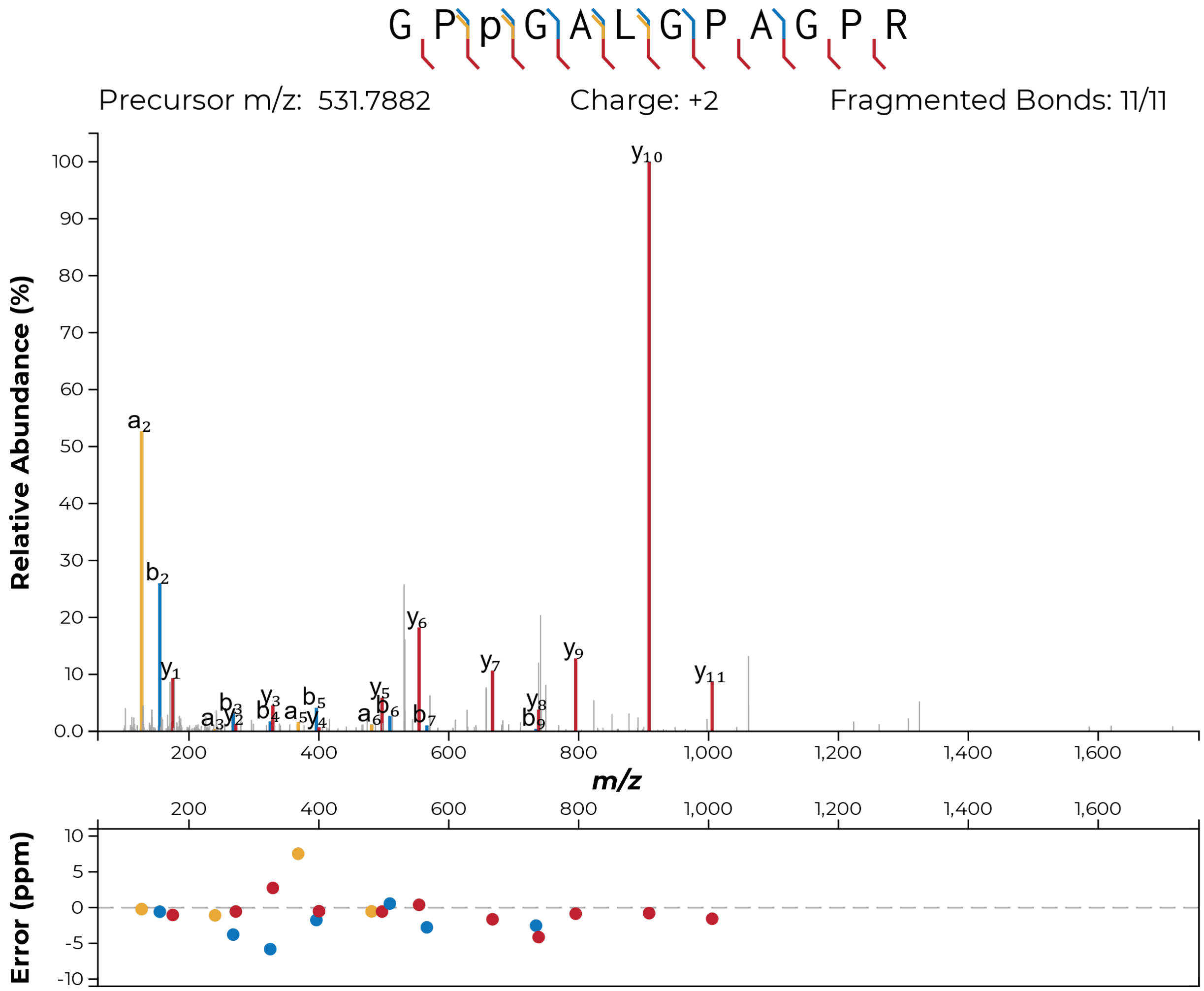

mm JACGTZ010000853_7a GPPGALGPAGPR scan 8237 score 135; Feliformia (not Panthera sp.)

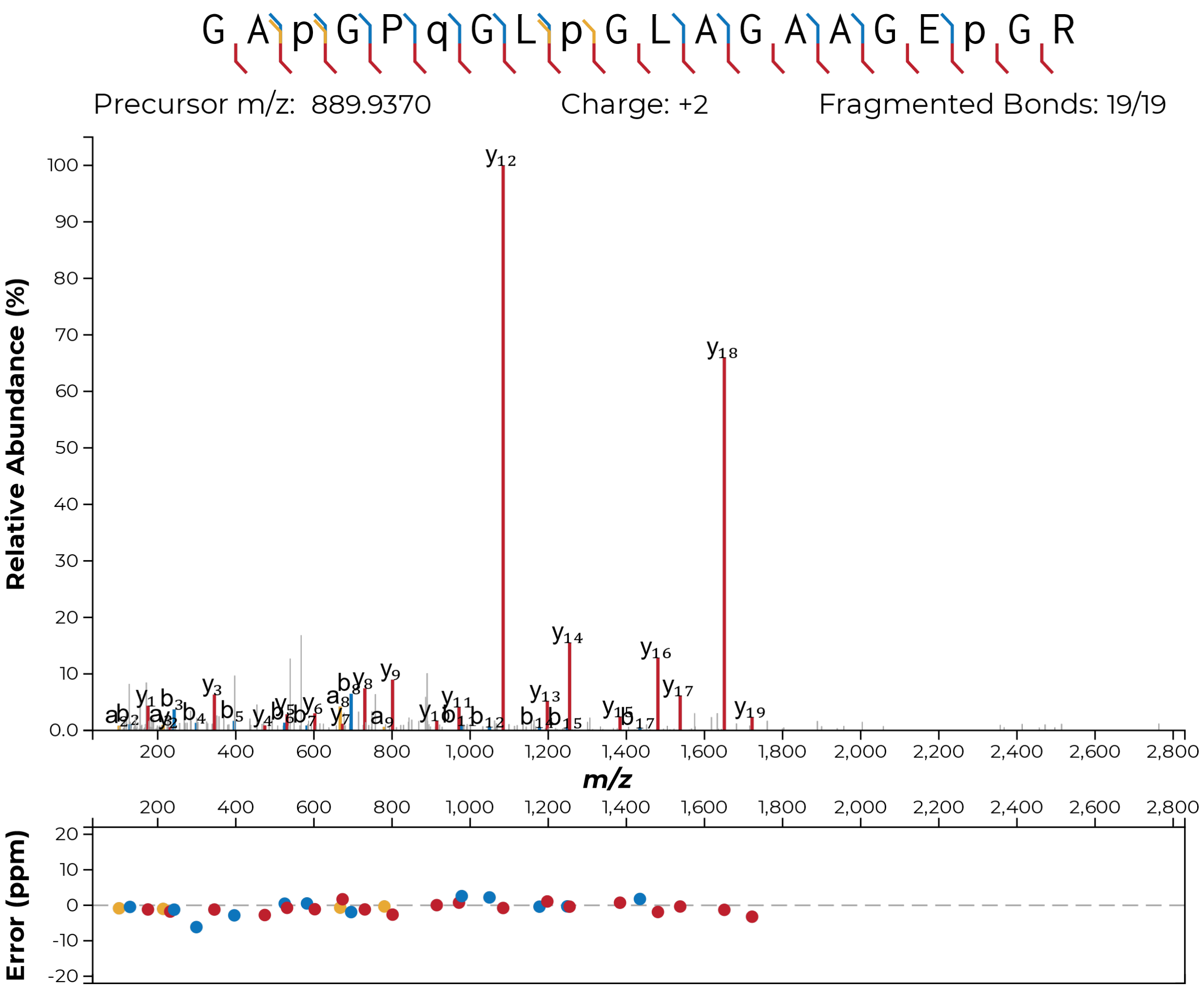

nn JACGTZ010000853_7a GAPGPQGLPGLAGAAGEPGR scan 14334 score 111; Feliformia + Chinchilla lanigera, Ochotona curzoniae, Oryctolagus cuniculus, Octodon degus

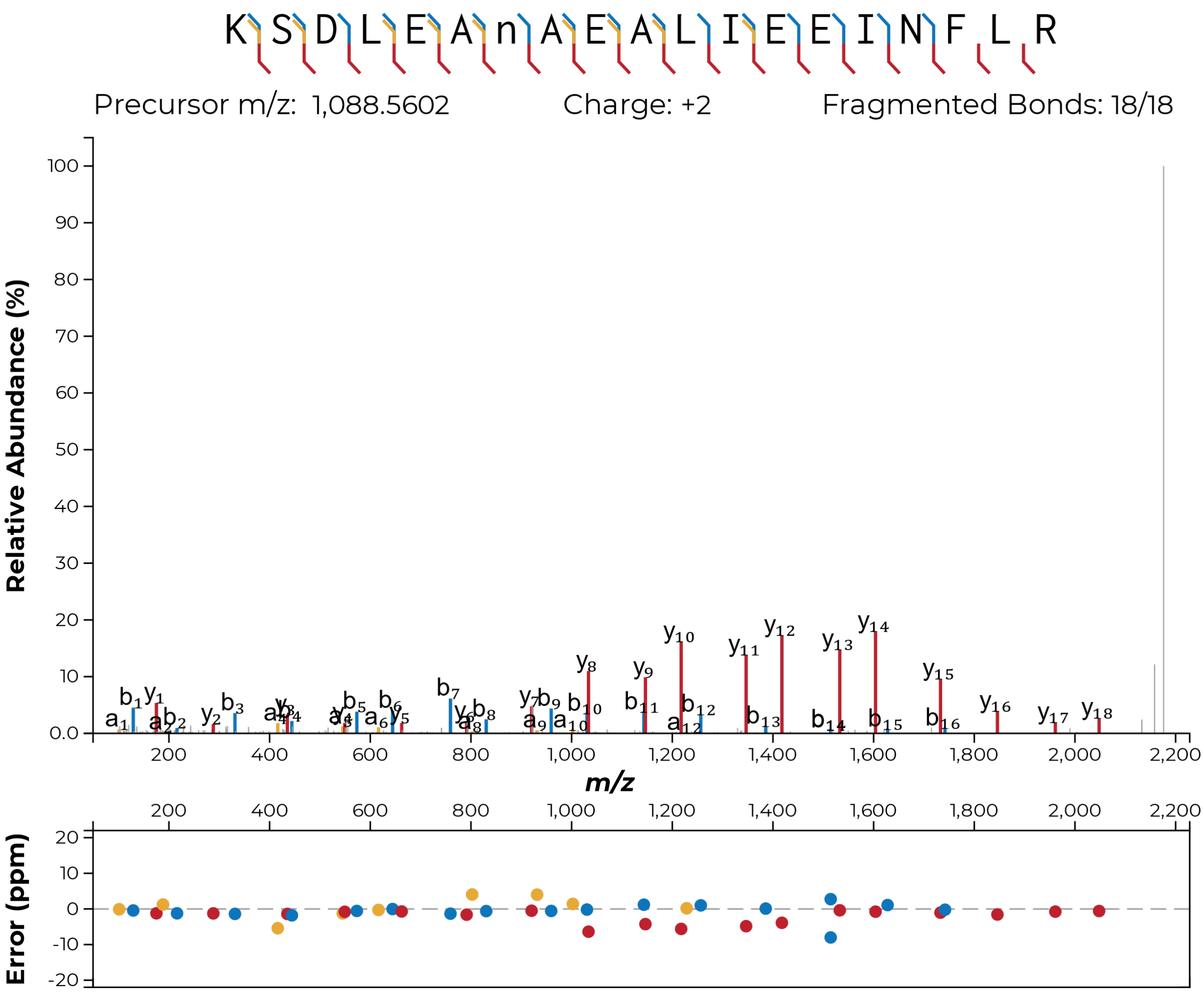

oo XP_046922113.1 KSDLEANAEALIEEINFLR scan 26184 score 370; Felidae ("Old World": Lynx pardinus, Felis catus, Prionailurus bengalensis/viverrinus, Panthera tigris) + Gracilinanus agilis

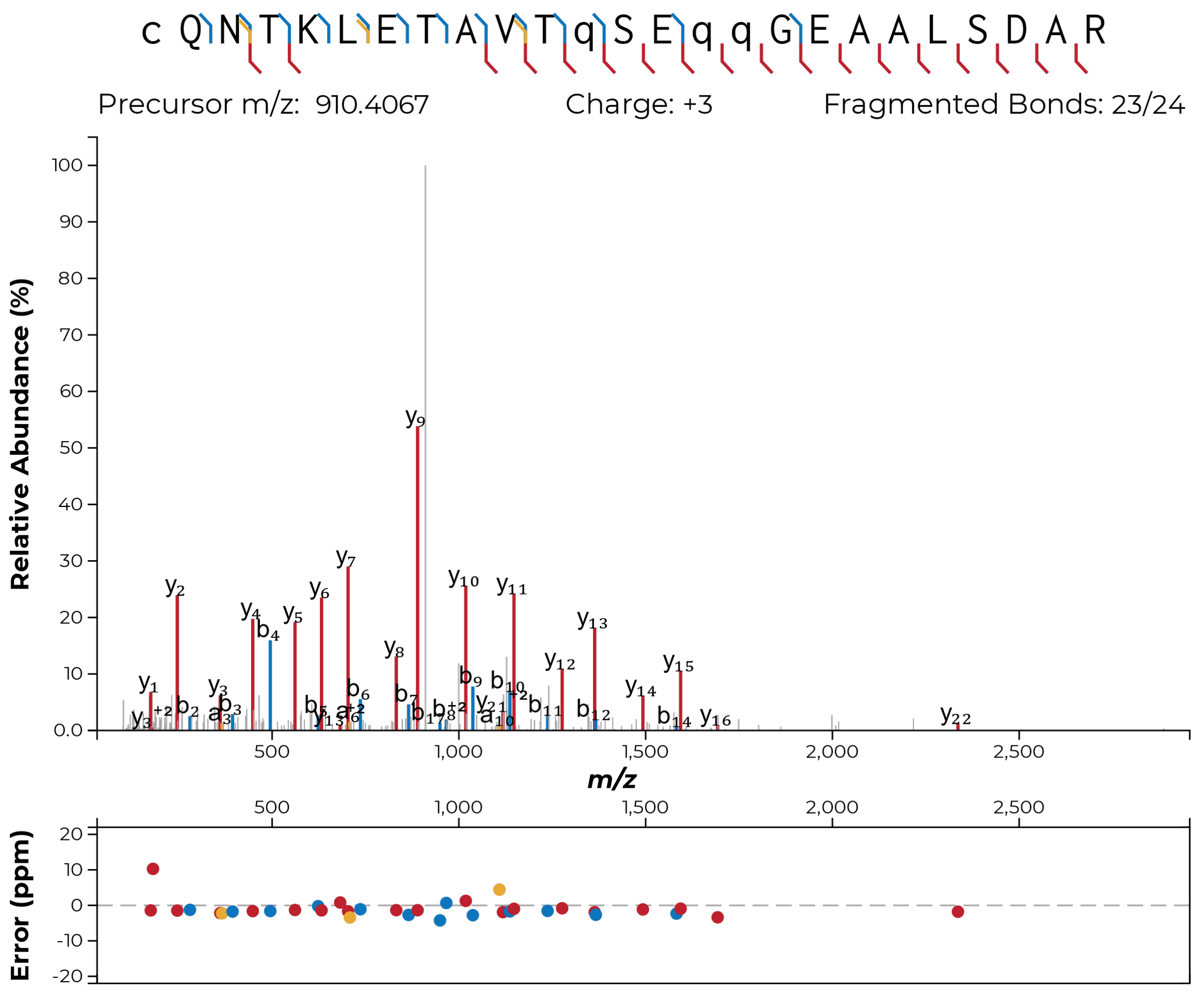

pp XP_046922113.1 CQNTKLETAVTQSEQQGEAALSDAR scan 22530 score 186; Feliformia ("Old World": Lynx pardinus, Felis catus, Prionailurus bengalensis/viverrinus, Acinonyx jubatus, Hyaena hyaena) + Sciuridae

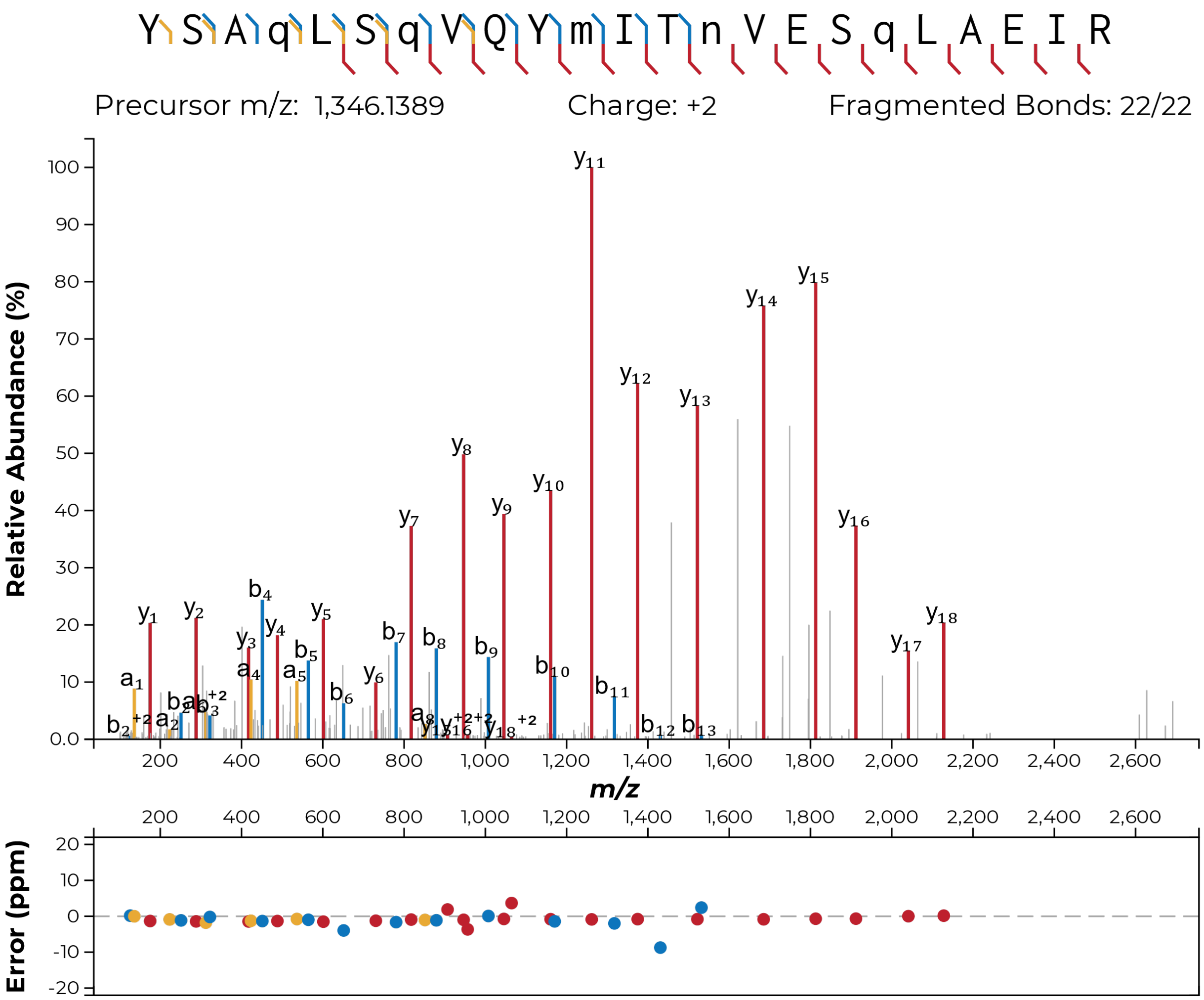

qq XP_026904859.1 YSAQLSQVQYMITNVESQLAEIR scan 25461 score 358; Acinonyx jubatus, Loxodonta africana

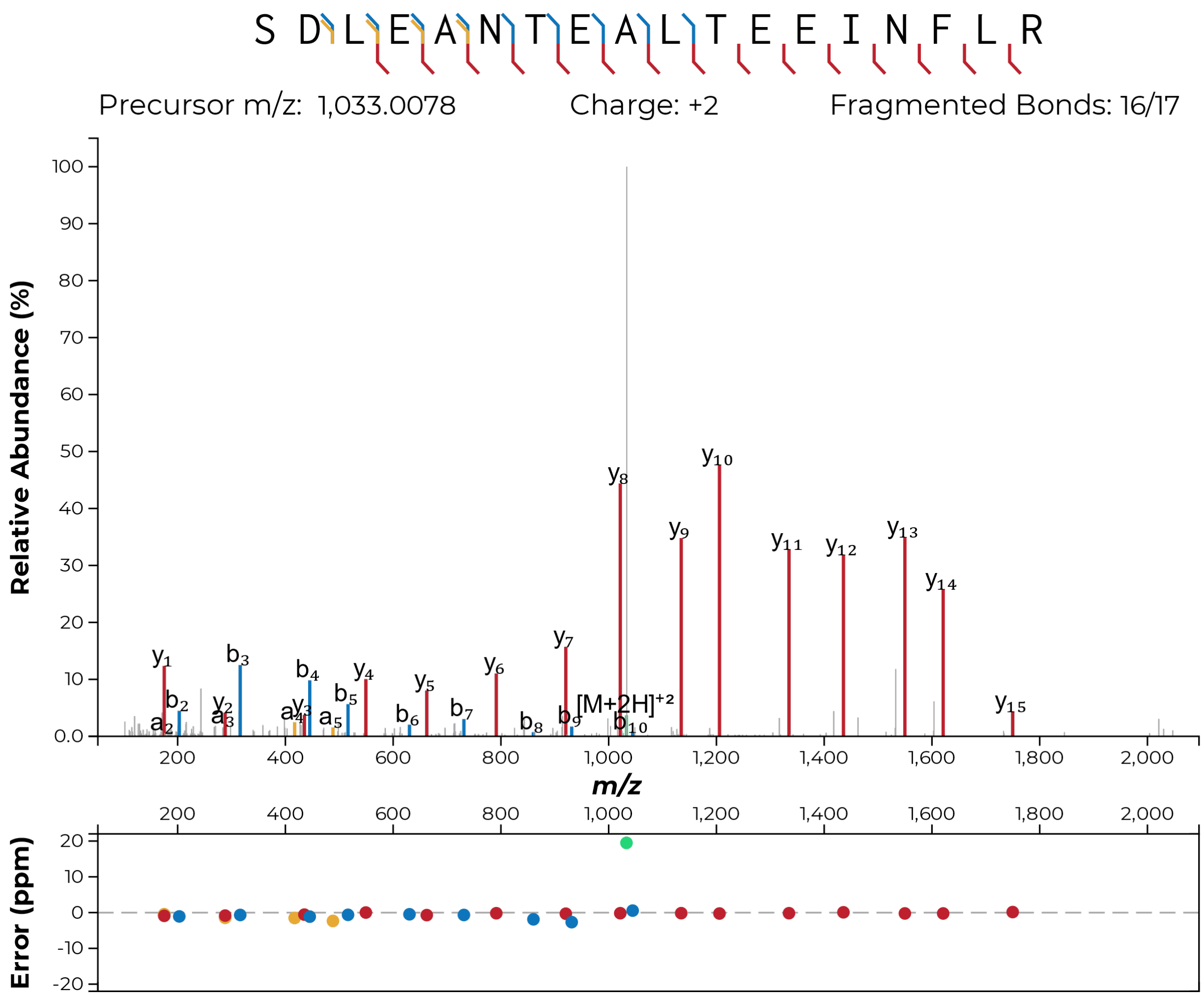

rr A0A6J1XHR0 SDLEANTEALTEEINFLR scan 24216 score 356; Acinonyx jubatus

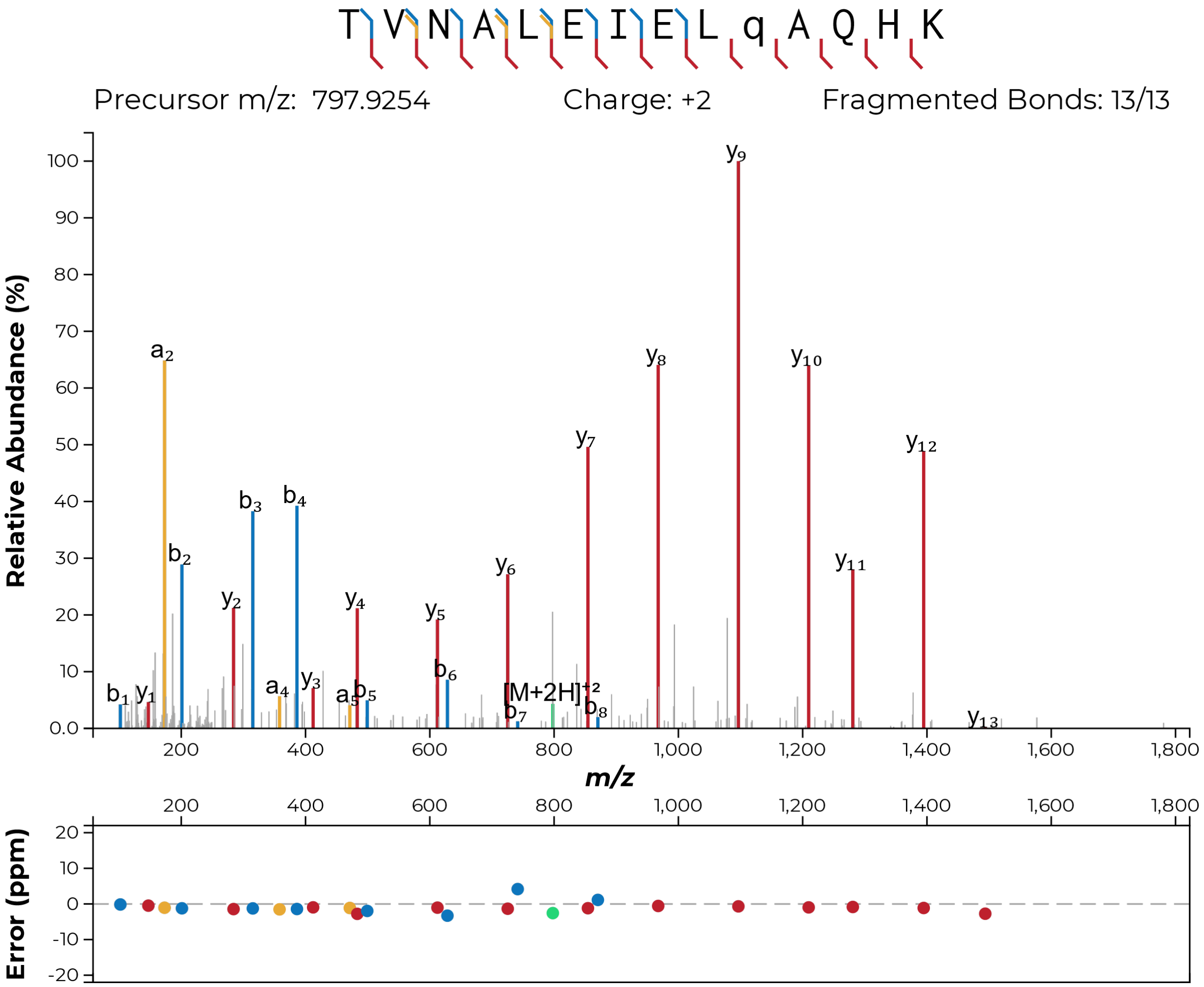

ss A0A6I9ZLE5 TVNALEIELQAQHK scan 16013 score 194; Acinonyx jubatus, Trichechus manatus latirostris, Ornithorhynchus anatinus, Tachyglossus aculeatus

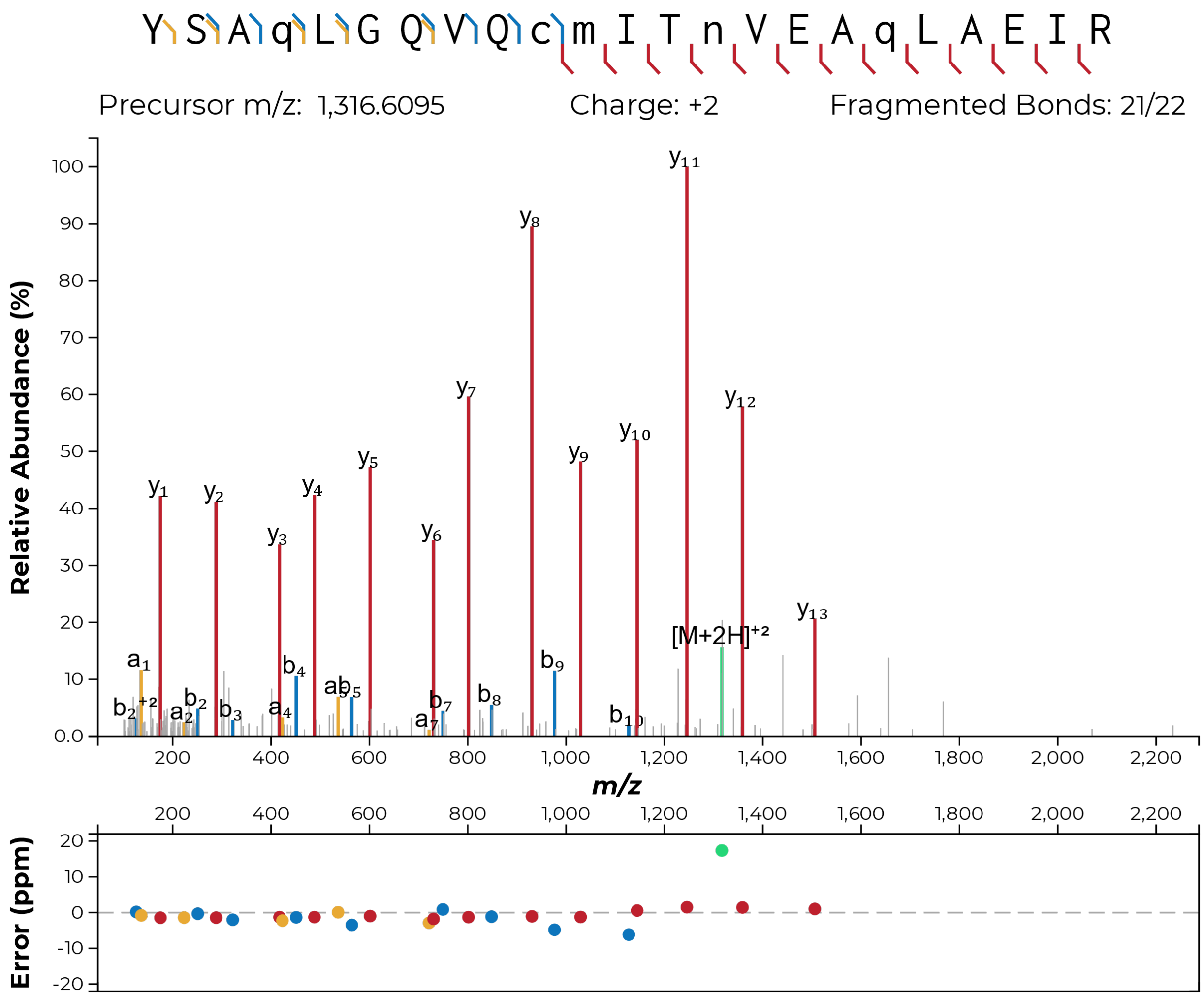

tt A0A6I9ZLE5 YSAQLGQVQCMITNVEAQLAEIR scan 25930 score 138; Acinonyx jubatus

C. ***Sample MG21 (uu-ddd)***

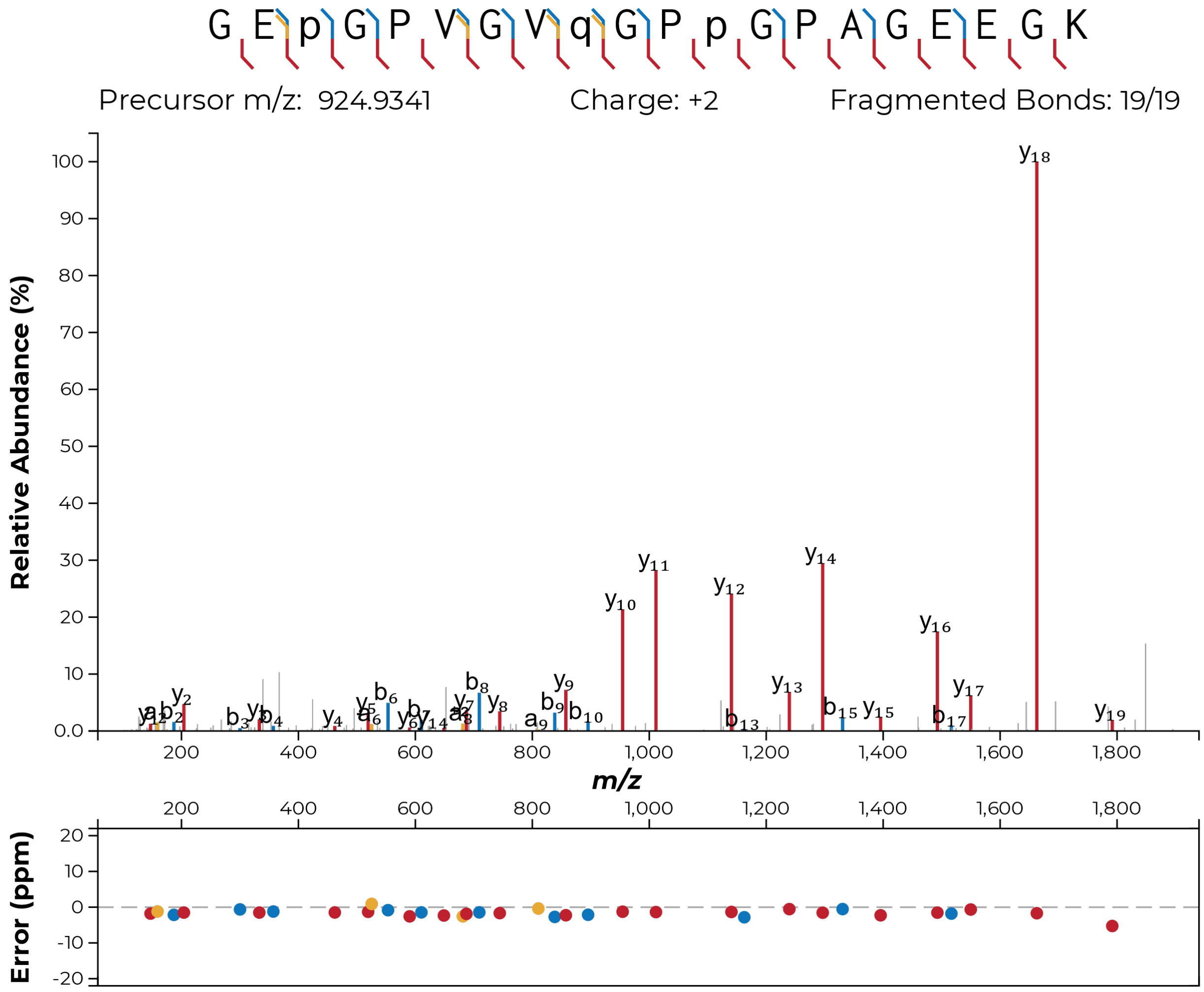

uu P02452 GEPGPVGVQGPPGPAGEEGK scan 9239 score 208; Mammalia but not Mus sp.

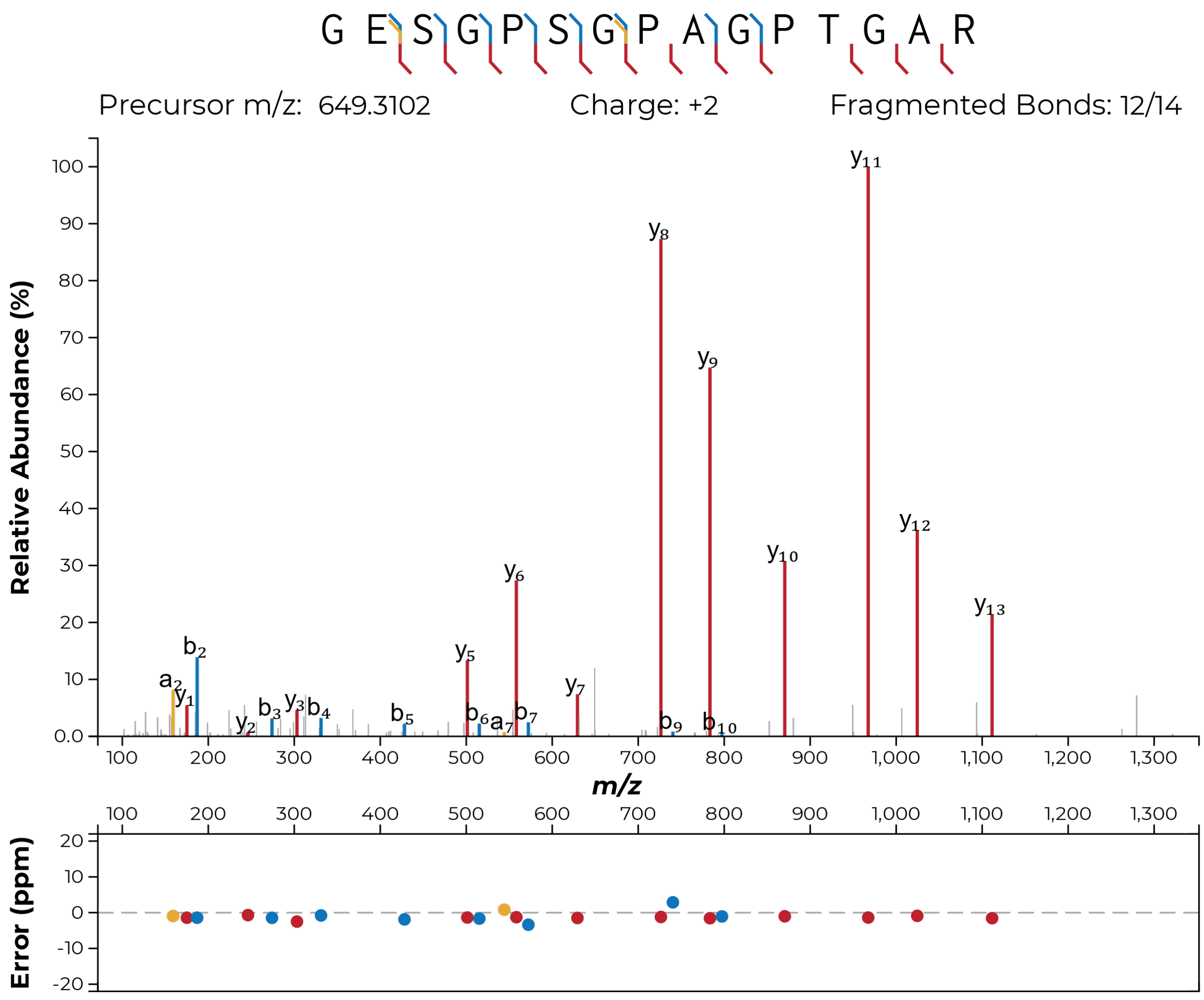

vv P02452 GESGPSGPAGPTGAR scan 4500 score 262; Amniota but not Mus sp. (only Hominoidea is Homininae)

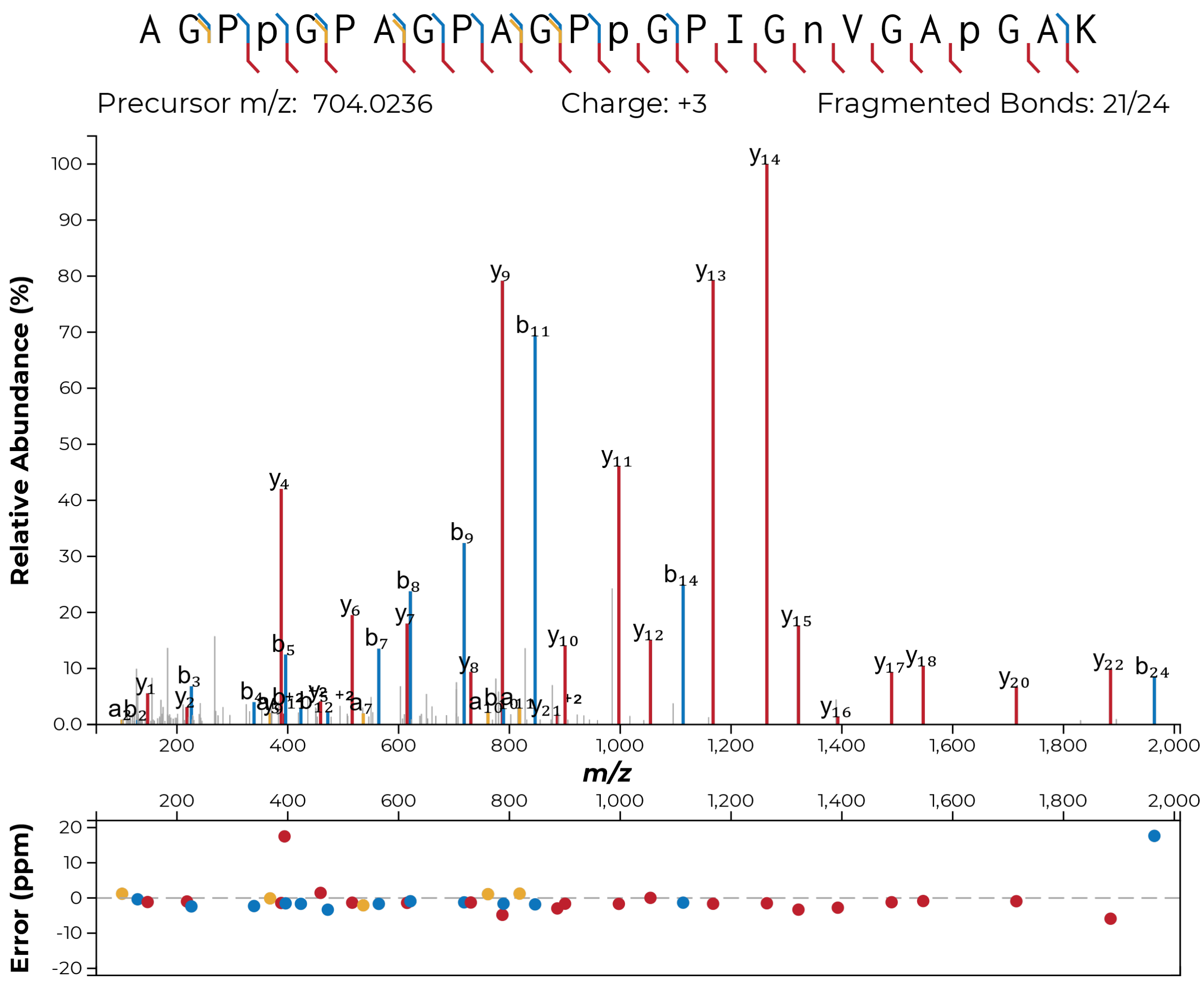

ww *P02452 AGPPGPAGPAGPPGPIGNVGAPGAK scan 11766 score 155; Hominoidea + Steptomyces sp. + Mus sp.*

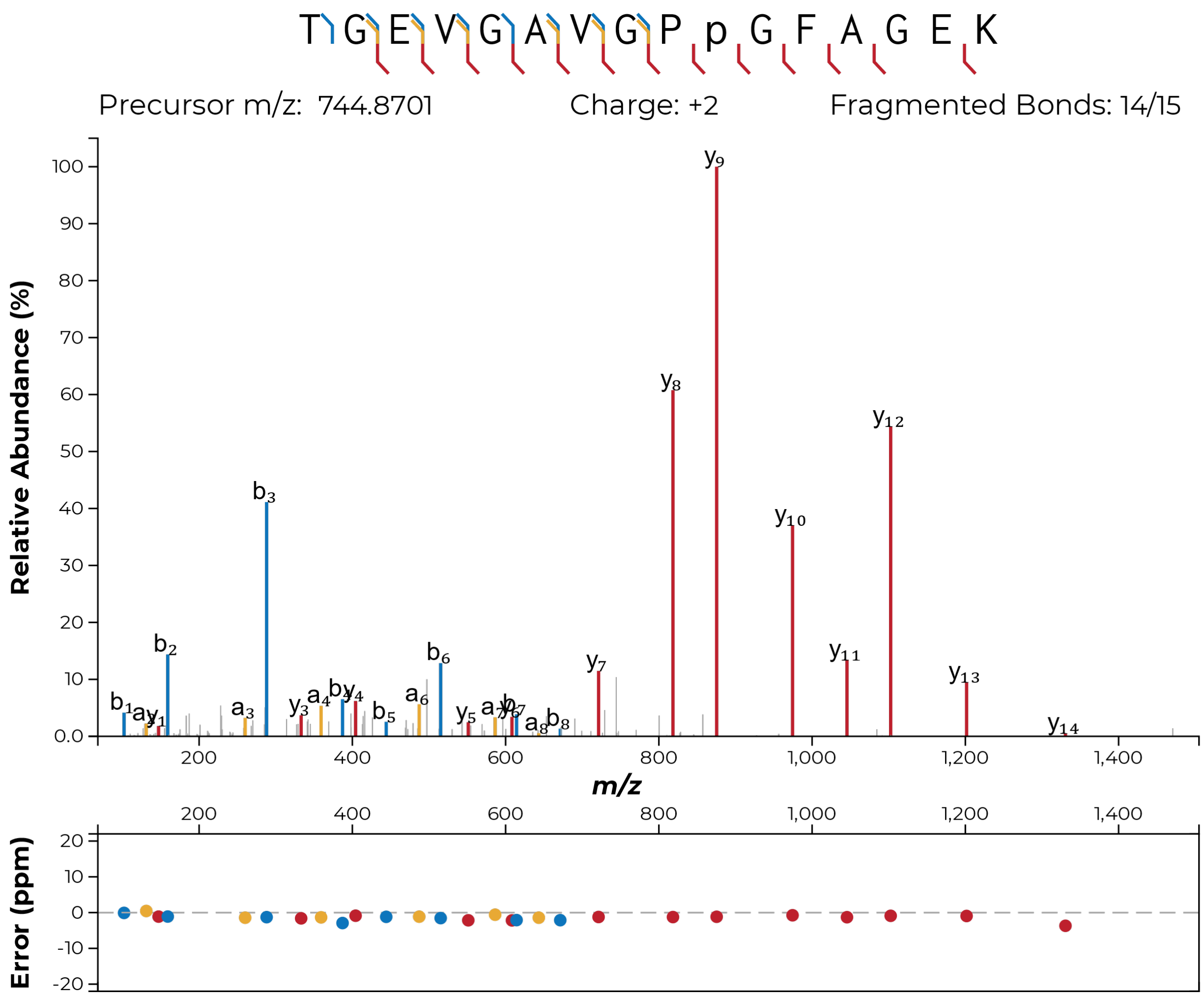

xx A0A087WTA8 TGEVGAVGPPGFAGEK scan 11269 score 158; Catarrhini

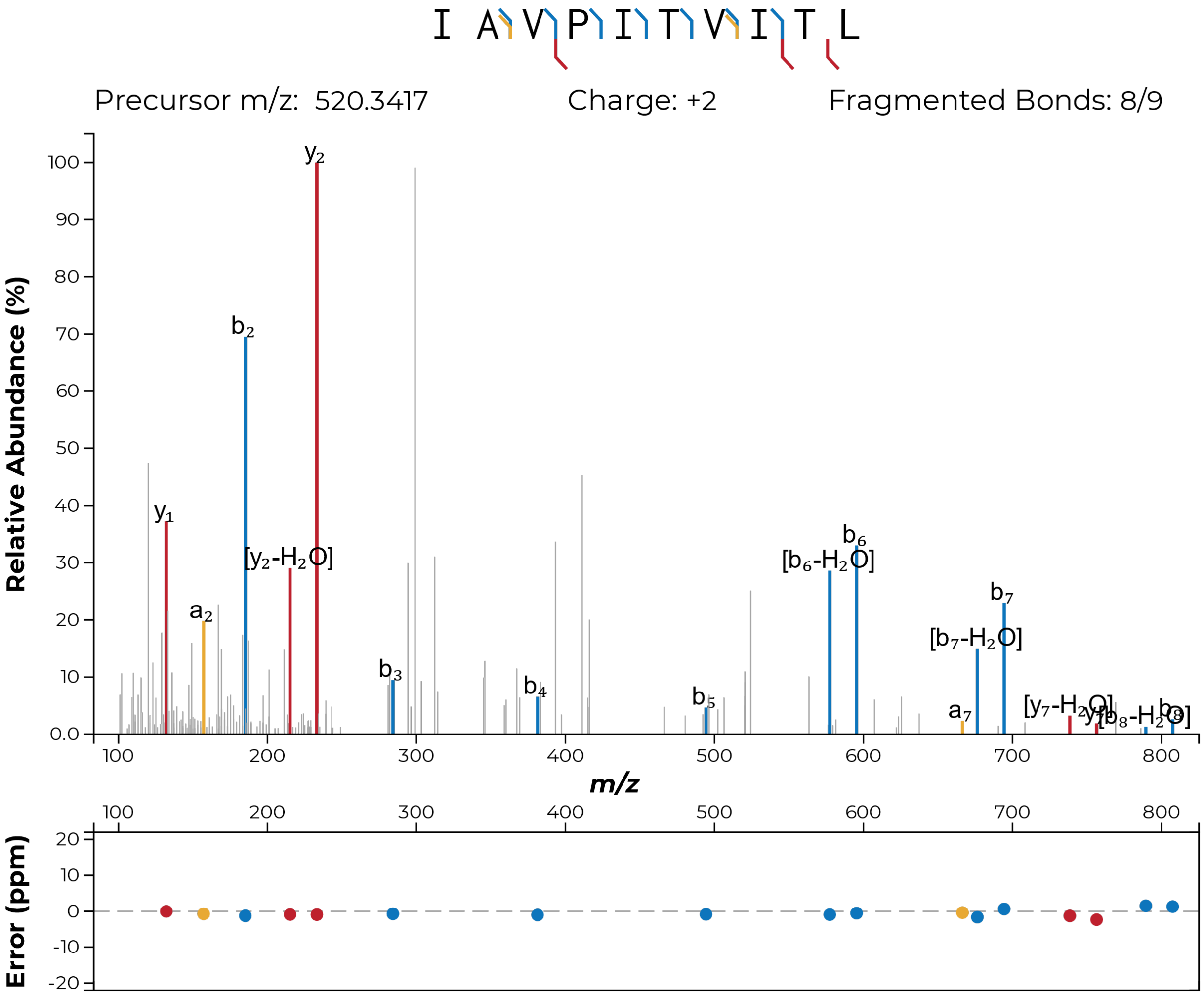

yy A0A0A0MTS7 IAVPITVITL scan 23826 score 116; Hominidae

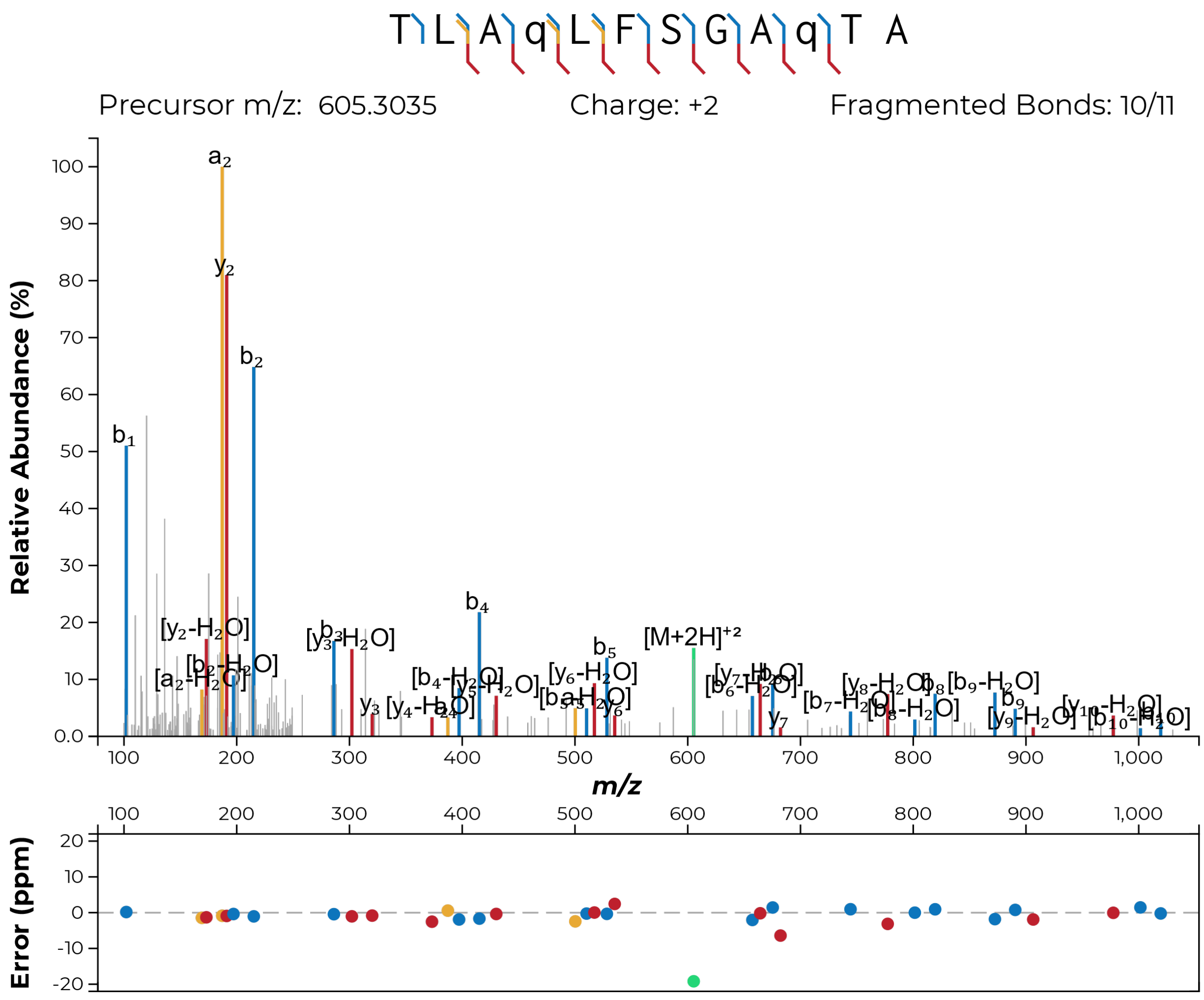

zz Q9UKX2 TLAQLFSGAQTA scan 21524 score 118; Homo sapiens, Ailuropoda melanoleuca

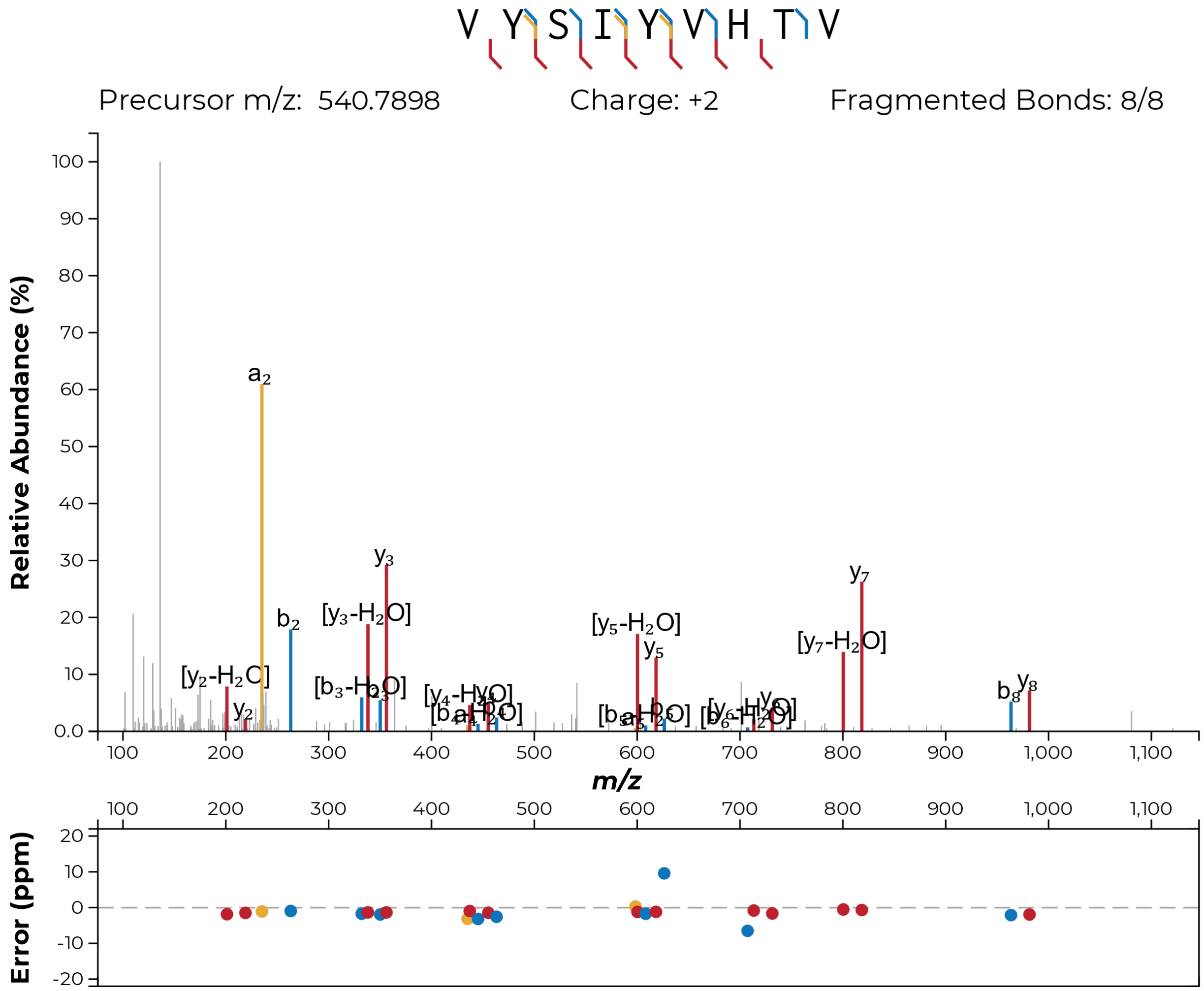

xx E9PCT5 VYSIYVHTV scan 14267 score 155; Catarrhini

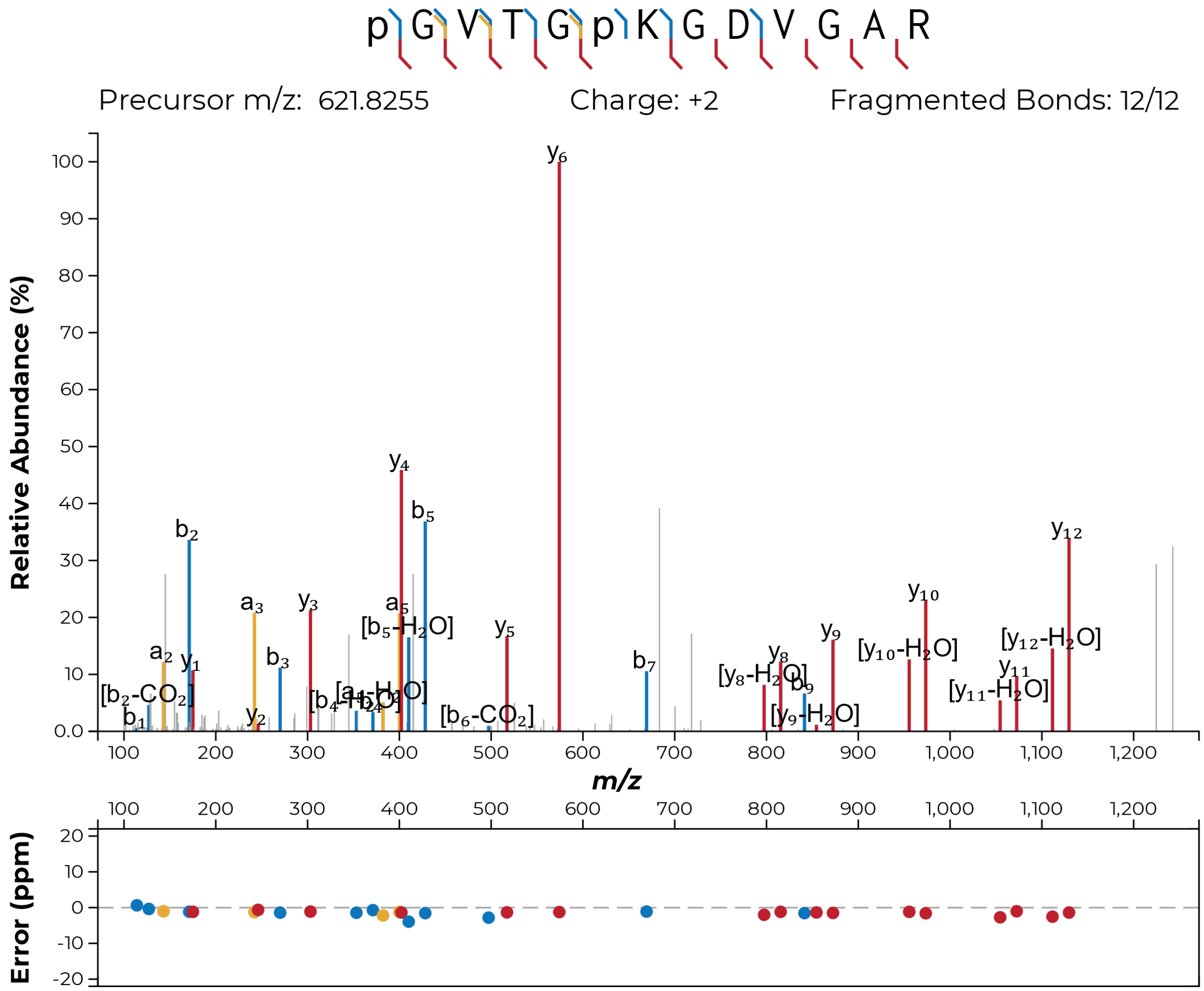

bbb A0A3B3ITQ8 PGVTGPKGDVGAR scan 4102 score 150; Homo sapiens, Sturnira hondurensis

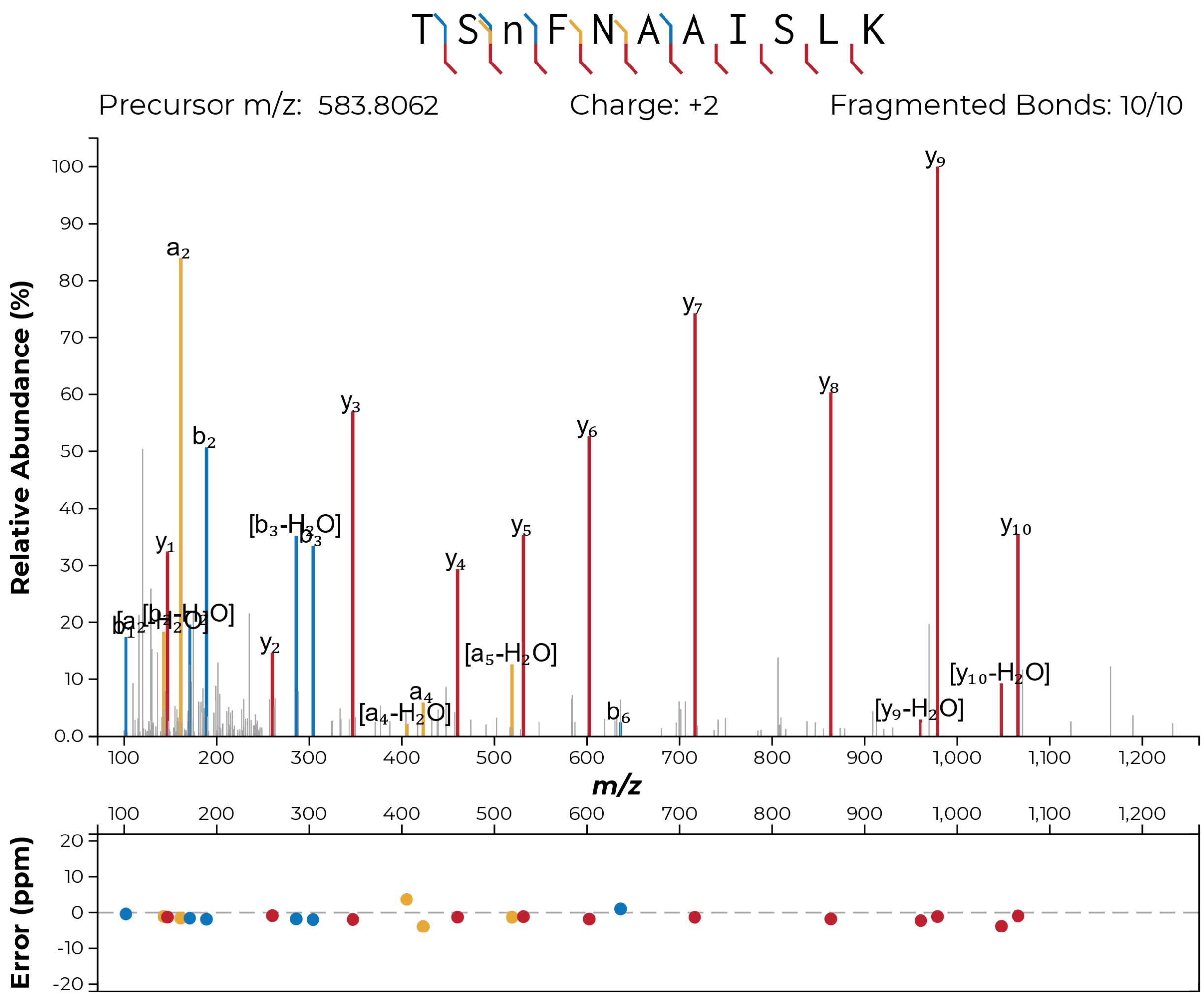

ccc P02748 TSNFNAAISLK scan 13988 score 133; Homininae

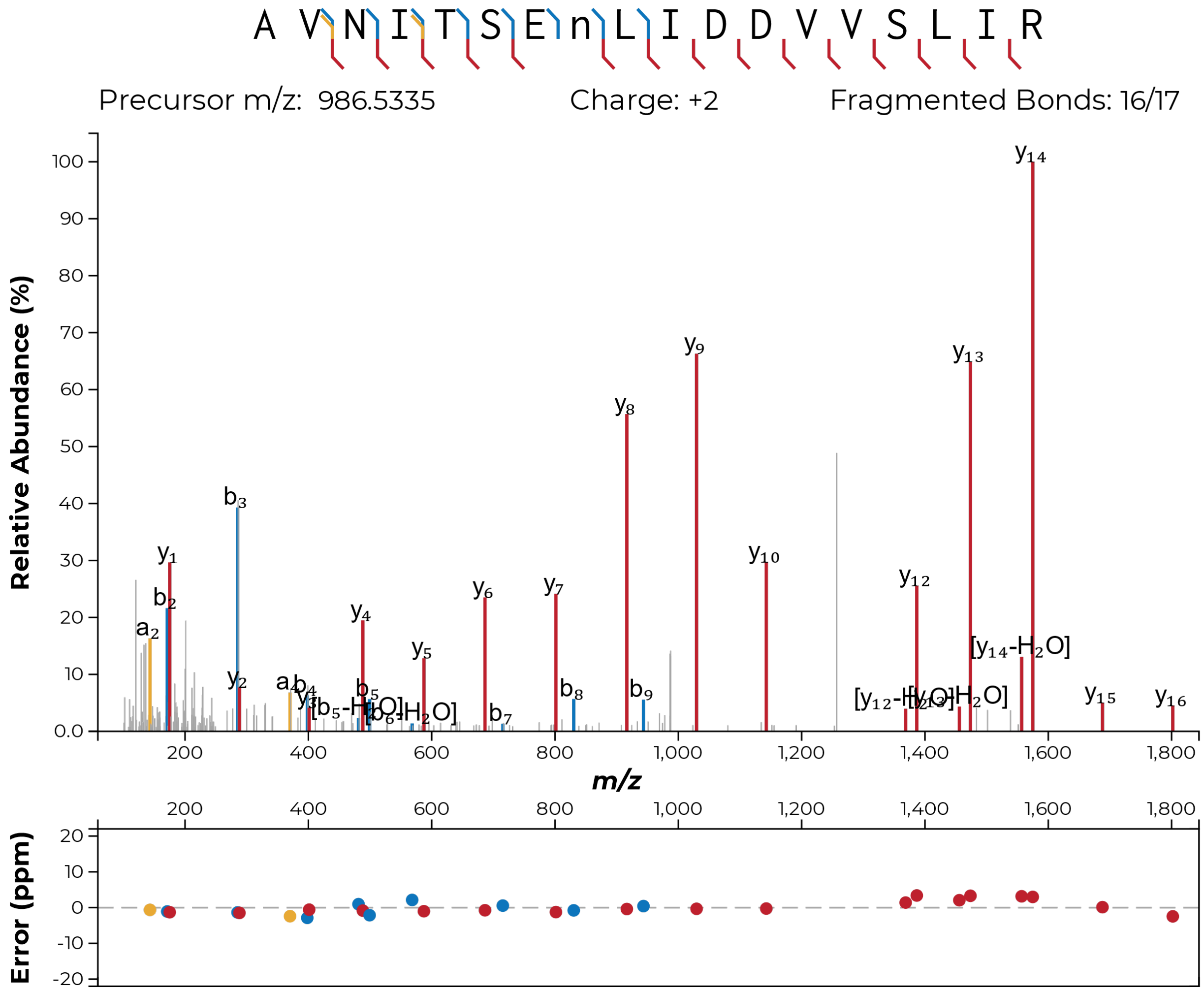

ddd P02748 AVNITSENLIDDVVSLIR scan 24855 score 156; Homininae
