# Supplementary material for: Human and animal skin identified by palaeoproteomics in Scythian leather objects from Ukraine
Source: PLoS One. 2023 Dec 13;18(12):e0294129. doi: 10.1371/journal.pone.0294129 (PMC10718408; doi:10.1371/journal.pone.0294129)
Supplement: Attachment — Submitted filename: Reply to reviewers.docx [file pone.0294129.s004.docx]

**Human and animal skin identified by palaeoproteomics in Scythian leather objects from Ukraine**

**Authors**

Luise Ø. Brandt^1^, Meaghan Mackie^1, 2^, Marina Daragan^3^, Matthew, J. Collins^1,4^, Margarita Gleba^5^

**Author responses to comments from the editors and reviewers**

**Editors’ comments**

1. Please ensure that your manuscript meets PLOS ONE's style requirements, including those for file naming. The PLOS ONE style templates can be found at
https://journals.plos.org/plosone/s/file?id=wjVg/PLOSOne_formatting_sample_main_body.pdf and
<https://journals.plos.org/plosone/s/file?id=ba62/PLOSOne_formatting_sample_title_authors_affiliations.pdf>

**Response**: we have changed to double line spacing and added supporting information captions

2. In your manuscript, please provide additional information regarding the specimens used in your study. Ensure that you have reported human remain specimen numbers and complete repository information, including museum name and geographic location.

If permits were required, please ensure that you have provided details for all permits that were obtained, including the full name of the issuing authority, and add the following statement:

'All necessary permits were obtained for the described study, which complied with all relevant regulations.'

If no permits were required, please include the following statement:

'No permits were required for the described study, which complied with all relevant regulations.'

For more information on PLOS ONE's requirements for paleontology and archeology research, see <https://journals.plos.org/plosone/s/submission-guidelines#loc-paleontology-and-archaeology-research>.

**Response**: We added the following statement in the materials section: 'No permits were required for the described study, which complied with all relevant regulations.'

The names and geographic locations of the museums where the objects analysed were kept at the time sampling were added in Table 1 and in S1. Please note that due to the ongoing war in Ukraine some of the objects may have been moved or destroyed. We added a sentence in Acknowledgements to acknowledge this.

3. Please note that funding information should not appear in any section or other areas of your manuscript. We will only publish funding information present in the Funding Statement section of the online submission form. Please remove any funding-related text from the manuscript.

**Response**: We have removed funding information from Acknowledgements.

4. We note that the grant information you provided in the ‘Funding Information’ and ‘Financial Disclosure’ sections do not match.

When you resubmit, please ensure that you provide the correct grant numbers for the awards you received for your study in the ‘Funding Information’ section.

**Response**: Thank you for noticing this. We will add: Philip Schwartz Initiative by the Alexander von Humboldt Foundation (project “Preserving Scythian Heritage for the Future”; Daragan), to the Financial Disclosure.

5. Thank you for stating the following financial disclosure:
 "This work was supported by the Alexander von Humboldt Stiftung and Gerda Henkel Stiftung (project “The production technology of Scythian Archery equipment: bow, arrows and quivers”; Daragan), and the Danish National Research Foundation (grant DNRF128 PROTEIOS)."

Please state what role the funders took in the study. If the funders had no role, please state: "The funders had no role in study design, data collection and analysis, decision to publish, or preparation of the manuscript."
If this statement is not correct you must amend it as needed.
Please include this amended Role of Funder statement in your cover letter; we will change the online submission form on your behalf.

**Response**: We added the funders role in the cover letter.

6. We note that you have stated that you will provide repository information for your data at acceptance. Should your manuscript be accepted for publication, we will hold it until you provide the relevant accession numbers or DOIs necessary to access your data. If you wish to make changes to your Data Availability statement, please describe these changes in your cover letter and we will update your Data Availability statement to reflect the information you provide.

**Response**: We agree to this and will provide the relevant information as soon as the manuscript is accepted for publication.

7. Please include your full ethics statement in the ‘Methods’ section of your manuscript file. In your statement, please include the full name of the IRB or ethics committee who approved or waived your study, as well as whether or not you obtained informed written or verbal consent. If consent was waived for your study, please include this information in your statement as well.

**Response**: We added the following statement in the Methods section: Since we did not anticipate to identify human remains, no ethics committee approval was required prior to this study. As human remains identified are 2400 years old, consent is not relevant to this study.

8. We note that Figure 1 in your submission contain map/satellite images which may be copyrighted. All PLOS content is published under the Creative Commons Attribution License (CC BY 4.0), which means that the manuscript, images, and Supporting Information files will be freely available online, and any third party is permitted to access, download, copy, distribute, and use these materials in any way, even commercially, with proper attribution. For these reasons, we cannot publish previously copyrighted maps or satellite images created using proprietary data, such as Google software (Google Maps, Street View, and Earth). For more information, see our copyright guidelines: http://journals.plos.org/plosone/s/licenses-and-copyright.

We require you to either (a) present written permission from the copyright holder to publish these figures specifically under the CC BY 4.0 license, or (b) remove the figures from your submission:

**Response**: The figure has been amended and the relevant sources clearly stated in the caption: Image: M. Daragan, based on a DTM STRM Map services and data available from U.S. Geological Survey, National Geospatial Program; Incert - DAI Eurasia Abteilung: <https://www.usgs.gov/centers/eros/science/usgs-eros-archive-digital-elevation-shuttle-radar-topography-mission-srtm-non>

9. Please include captions for your Supporting Information files at the end of your manuscript, and update any in-text citations to match accordingly. Please see our Supporting Information guidelines for more information: http://journals.plos.org/plosone/s/supporting-information.

**Response**: This has been added.

10. Please review your reference list to ensure that it is complete and correct. If you have cited papers that have been retracted, please include the rationale for doing so in the manuscript text, or remove these references and replace them with relevant current references. Any changes to the reference list should be mentioned in the rebuttal letter that accompanies your revised manuscript. If you need to cite a retracted article, indicate the article’s retracted status in the References list and also include a citation and full reference for the retraction notice.

**Response**: a few references have been added as a reply to a reviewer comment and the list of references has been updated accordingly.

**Reviewers' comments**:

**Reviewer #1**: The manuscript “Human and animal skin identified by palaeoproteomics in Scythian leather objects from Ukraine” is an interesting application of ZooMS and LC-MS/MS for leather identification. The archaeological context, objects and interpretation of the data are well described. The results are however rushed and the paper would benefit from further details. More specifically:

There is a microscopy paragraph in Methods. The paragraph describes results rather than the method employed. Either this paragraph should be moved to results and detailed or removed entirely.

**Response**: We thank the reviewer for this comment. We moved part of the paragraph to the introduction since the attempts to use microscopy preceded this study and led towards the application of palaeoproteomics, so this constitutes the background of the present study.

In LC-MS/MS methods, you mention searching the samples against a Swissprot database, and later that “iterative searches were made to narrow down the database to the relevant proteins from the relevant species.” This was repeated in Supplementary but no further detail is provided. The accession numbers in Supplementary for samples 48 and 49 appeared to be from NCBI. Please provide more precision on which databases were searched.

**Response**: We thank the reviewer for their interest in the searching strategy. More detail of the this and the specific database source and content has been added to the supplementary:

“The databases used to narrow down identifications after indications from Swissprot varied between samples. For sample MG21 (21 from Bulhakovo, kurgan 5, burial 2), after the determination of hominin origin from the Swissprot search, the human proteome from Uniprot (downloaded 25/11/20) was searched. In an attempt to improve identifications, a database was made solely of the protein hits from this search (downloaded 05/01/2022) and searched again for final identifications.

For the apparent non-hominin samples (F10/F11), a database of mammalian collagens 1-3 and keratins was made from both Uniprot and NCBI (downloaded 31/05/21) after the Swissprot searches. The hits from this database led to further narrowing of the databases. For sample F10 (48 from Ilyinka, kurgan 4 burial 3), a database was composed first of Rodentia collagen 1-3 and keratin sequences from both Uniprot and NCBI (downloaded 09/08/2021), which indicated Scuridae origin. A database of Scuridae collagen 1-3 and keratin sequences from both Uniprot and NCBI (downloaded 09/11/2021), with additional predicted keratin sequence fragments from *S. vulgaris* [(Brandt et al. 2022)](https://paperpile.com/c/3SZ0Of/7Qzn) was then used for final identification[.](https://paperpile.com/c/3SZ0Of/7Qzn) For sample F11 (Vil’na Ukraina 4, kurgan 22 burial 1), a database was composed first of Carnivora collagen 1-3 and keratin sequences from both Uniprot and NCBI (downloaded 07/02/2022), which indicated Felidae origin. A database of Felidae collagen 1-3 and keratin sequences from both Uniprot and NCBI (downloaded 12/07/2022), with additional hypothetical keratin and collagen sequences created from tBLASTn searching of the genomes from *Felis chaus* (jungle cat), *Felis nigripes* (Black-footed cat) and *Caracal caracal* (caracal) based on the proteins recovered, was used for final identification.”

I could not find Figures S2… I only have access to S1.pdf and S1.dataset

**Response**: This is true and we are very sorry. We have added it to the new submission.

The results paragraph is very short and some information provided in Supplementary could be moved there to offer more insights into the results.

In the species identification paragraph, it would be helpful if you could indicate the sample number when you describe specific samples, especially for the wild carnivores.

**Response**: Sample numbers have been added to the carnivores, the two horse samples and the cattle sample.

I would also indicate somewhere the common name of species alongside the Latin names (in Supplementary tables as well).

**Response**: Common names have been added in Table 2. Since the supplementary data is primarily used by specialists who are familiar with Latin species nomenclature, we left only Latin names in Supplementary tables.

It is unfortunate that only one human sample was analyzed by LC-MS/MS. The sample was chosen due to low resolution by ZooMS. It would have been interesting to compare the results on both samples by LC-MS/MS to understand why one was of lower quality.

**Response**: We definitely agree that this would be very interesting to do in the future. For the purpose of species ID however, this was not required.

Furthermore, you have 26% of unidentified samples, which is quite high. Do you have any clues to explain the unidentified samples? Is this a problem with the method, the context in which objects were found, treatment of the leather or preservation issues?

**Response**: We thank the reviewer for this comment. Based on our experience with ZooMS on archaeological leather, the success rate varies quite a lot from site to site, suggesting that the specific preservation conditions have a big influence on collagen preservation. In this study, the samples come from multiple sites at which the conditions may vary a lot. For archaeological leather, the tanning process is also supposed to influence collagen preservation and those two factors probably explain why we are seeing lower success rates in archaeological leather compared to for instance observed for parchment. We have clarified this with two references:

Thirty-three of 45 samples analysed with ZooMS were identified, which is within the expected success rate for archaeological leather [(Brandt et al. 2020; Tarasov et al. 2022)](https://paperpile.com/c/HTQa7r/gkHi+oTmJ).

Tables S7 and S8 have the wrong descriptions and one is missing sample name.
**Response**: Thank you for noticing our mistake that must have happened when we copied tables into the document. We have switched the table descriptions between S7 and S8 so that they now reflect the true contents. In addition, we have added the missing sample number “49” to the description of S8 in the table of contents at the beginning of the supplementary.

Table 3: “Number of proteins and corresponding proteins”. I suppose you mean corresponding peptides.

**Response**: We thank the reviewer for noticing this typo and it has been corrected.

In discussion, since you mostly talk about quivers, it would be useful to have a drawing showing the different parts of the quiver. While you might not know which part of the object each sample corresponds to, as much as possible, some discussion between the species identified and corresponding part of the object would help to put the results into light. Perhaps as highlighted in a drawing/figure.

**Response**: Thank you for this suggestion. We have added a new figure (Fig 3), which shows the approximate location of the samples within the quivers from various sites. We also added a section in The discussion called “Leather uses and functions” that deals with the use of different species for different items analysed in this study, with a particular focus on quivers.

**Reviewer #2**: This is a very interesting study on identifying the species of origin for Scythian leather artefacts using both PMF and LC-MS-based approaches. The study is methodologically sound and the manuscript is very well written with a clear, logical flow. However, there are some minor comments that I believe the authors should consider.

---------------

Figure 2:
Do the scale bars depict cm units? Maybe add a brief sentence to the figure caption for clarification.

**Response**: Yes, they are cm and we have now added this to the caption.

Results:
Inconsistency – Table 2 reads “Kurgan 4, burial 2” for sample 7 from Ilyinka which was identified as human, however the main text referring to this sample reads “kurgan 2, burial 4”. This is seen again in Figure 5 caption which also reads "kurgan 2, burial 4". Based on what the authors have presented in Table 1, should the site read "kurgan 4, burial 2" in all instances?

**Response**: Thank you for noting this inconsistency - this has been corrected in both instances.

Supplementary Information:
Reference is made in the Methods (section Liquid chromatography-tandem mass spectrometry) and Supplementary Information S1 to Supplementary Information S2 representing spectral annotations, however S2 doesn't seem to be attached to the current manuscript submission.

**Response**: This is true and we are very sorry. We have added it to the new submission.

Minor typographical corrections:

Orphaned round bracket “)” and extra square bracket “]” in the following Introduction sentence:
“For the purpose of the present study, Scythians are understood to be the nomads that occupied the steppes north of the Black Sea, and between the Danube and the Don Rivers, as defined by the ancient Greek ‘father of history’ Herodotus [17–19]])”

**Response**: Thank you for noticing. This has been changed.

Figure 3 caption: Random comma and space after image credit.

**Response**: Both of those have been removed.

Table 2 caption: Mustela should be italicised and capitalised as it is a genus name.

**Response**: Thank you for this comment. We have changed this.
